# Changing Landscape of Invasive Pneumococcal Disease Serotypes and Antimicrobial Resistance Following Pneumococcal Conjugate Vaccine Introduction in the Middle East and North Africa Region: A Systematic Review

**DOI:** 10.3390/vaccines13090923

**Published:** 2025-08-29

**Authors:** Zeinab El Zein, Mayse Nasser, Celina F. Boutros, Nadim Tfaily, Lina Reslan, Kawthar Faour, Sarah Merhi, Stephanie Damaj, Mohammad Bahij Moumneh, Tarek Bou Dargham, Nour Youssef, Magda Haj, Samer Bou Karroum, Sarah Khafaja, Aia Assaf Casals, Sarah Chamseddine, Layal Hneiny, Ghassan S. Dbaibo

**Affiliations:** 1 Center for Infectious Diseases Research (CIDR) and WHO Collaborating Center for Reference and Research on Bacterial Pathogens, Faculty of Medicine, American University of Beirut, Hamra, Beirut 1107, Lebanon; zeinab.elzein18@gmail.com (Z.E.Z.); maysenasser@gmail.com (M.N.); cb39@aub.edu.lb (C.F.B.); nt46@aub.edu.lb (N.T.); reslanlina@outlook.com (L.R.); kawtharfaour@gmail.com (K.F.); merhi223@gmail.com (S.M.); stephanie.damaj@hotmail.com (S.D.); mmm106@mail.aub.edu (M.B.M.); ny31@aub.edu.lb (N.Y.); maggiehaj93@gmail.com (M.H.); samerbk@outlook.com (S.B.K.); sk159@aub.edu.lb (S.K.); aassafcasals@gmail.com (A.A.C.); sera.ch13@gmail.com (S.C.); 2 Department of Pediatrics and Adolescent Medicine, American University of Beirut Medical Center, Hamra, Beirut 1107, Lebanon; 3 Pediatric Infectious Diseases Division, Department of Pediatrics and Adolescent Medicine, American University of Beirut Medical Center, Hamra, Beirut 1107, Lebanon; 4 Saab Medical Library, American University of Beirut, Hamra, Beirut 1107, Lebanon; layalhneiny@gmail.com

**Keywords:** *Streptococcus pneumoniae*, invasive pneumococcal disease (IPD), serotype, pneumococcal conjugate vaccine (PCV), antimicrobial resistance (AMR), non-susceptibility, Middle East and North Africa Region (MENA), review, vaccine type (VT), non-vaccine type (NVT)

## Abstract

**Background/Objectives:** Pneumococcal conjugate vaccines (PCVs) have significantly reduced invasive pneumococcal disease (IPD) globally. We conducted a systematic review to assess whether serotype and antimicrobial resistance trends in the Middle East and North Africa (MENA) reflect global patterns post-PCV introduction. **Methods:** We searched the CINAHL, MEDLINE, PUBMED, EMBASE, Global Health, Global Index Medicus, EBSCO, Scopus, and Cochrane databases for articles published from inception to 24 January 2024. Eligible studies were original articles in English or French, reporting IPD serotype distribution or antimicrobial susceptibility in the MENA region. Risk of bias was assessed using the STROBE checklist. **Results:** Eighty-nine studies from 18 countries were included. A decline in PCV7 serotypes was observed following the introduction of PCV10 or PCV13, which was more pronounced in PCV10-era studies. Serotype 3 increased post-PCV13 era, while 19A declined only after PCV10. An expansion in PCV20 serotypes and non-vaccine types (NVTs) was noted in PCV13-implementing countries. Antimicrobial resistance data were insufficient to provide a reliable trend. **Limitations:** There was limited AMR data and variable surveillance quality across countries. **Conclusions:** PCV introduction resulted in a modest decrease in PCV7 serotypes and a variable impact on PCV13 serotypes. This, along with the increase in PCV20 serotypes, indicates that higher-valency PCVs might provide better serotype coverage in the region. The study highlights the need for more robust surveillance across the region. **Registration:** CRD42018104529.

## 1. Introduction

Invasive pneumococcal disease (IPD), defined as an infection from *Streptococcus pneumoniae* isolated in a normally sterile body fluid or compartment, is a major global threat across age groups and, in particular, children, elderly, and immunocompromised individuals [1,2]. The World Health Organization (WHO) estimates that *S. pneumoniae* continues to kill more than 300,000 children under 5 years of age worldwide every year. It is a leading cause of complicated pneumonia, where global data analysis in 2016 highlighted its dominant role among pathogens contributing to lower respiratory tract infections and subsequent mortality [1,3].

The polysaccharide capsule of *S. pneumoniae* is the bacteria’s major virulence factor as it plays a role in colonization, adherence, and evasion of the immune system [4]. The capsular antigens of the common pathogenic serotypes were used to develop the original 23 valent pneumococcal polysaccharide vaccine (PPV23), which is not sufficiently immunogenic in children younger than 5 years of age [5,6]. The pneumococcal conjugate vaccines (PCVs) that were later developed are substantially more immunogenic in young children and target the most common disease-causing serotypes [1,2,7]. PCV7 was introduced in 2000 and included serotypes: 4, 6B, 9V, 14, 18C, 19F, and 23F. PCV10 and PCV13 were introduced in 2010 with the addition of 3 (serotypes 1, 5, and 7F) and 6 (serotypes 1, 3, 5, 6A, 7F, and 19A) serotypes, respectively [8].

These conjugate vaccines had an instrumental role in decreasing the burden of IPD in children, and consequently in adults, by conferring herd immunity [6]. In a recent systematic review, the rate of breakthrough infections with vaccine serotypes in children ≤ 5 years was low with the use of PCV10 and PCV13; confirming the high effectiveness of these vaccines [9]. Surveillance data over extended periods, mostly from USA and Europe, revealed a decrease in overall PCV7 serotypes with persistence of certain serotypes, an increase or persistence of serotypes 3 and 19A, and increase in NVTs [10,11,12,13,14,15].

In this regard, higher valency PCVs were recently approved to address persistent IPD caused by additional serotypes. Initially in June 2022, the Advisory Committee on Immunization Practices recommended the use of PCV15 in children; as USA surveillance data had shown that the two additional serotypes unique to PCV15 caused 15% and 23% of IPD in children <5 years and those between 5 and 18 years of age, respectively [16]. As for PCV20, later approved in 2023, the data from high income PCV13 countries revealed that this higher valency vaccine would cover an additional 38.2% of IPD serotypes [17].

Although IPD poses a major health challenge in the Middle East and North Africa (MENA) region, not all countries introduced PCVs into their Extended Program of Immunization (EPI). Moreover, data describing the pattern of serotype distribution, rates of vaccine compliance, and emergence of antibiotic resistance are scarce.

In the current systematic review, we aimed to determine whether PCV introduction, in countries where this data is available, had altered the landscape of IPD in a manner consistent with global trends. Our secondary objective was to assess the influence of vaccination on antimicrobial resistance (AMR). The results would be valuable in evaluating current vaccine impact and influencing future decisions on higher valency vaccine introductions.

## 2. Materials and Methods

### 2.1. Search Strategy

We conducted a systematic review in accordance with the PRISMA 2020 guidelines. We searched the literature up to 24 January 2024, using nine electronic databases: CINAHL, MEDLINE, Cochrane, PubMed, EMBASE, Global Health, Global Index Medicus, EBSCO, and Scopus. The search aimed to identify studies reporting on *Streptococcus pneumoniae* serotype distribution and antimicrobial resistance in the MENA region, including: Afghanistan, Algeria, Bahrain, Djibouti, Egypt, Iran, Iraq, Jordan, Kuwait, Lebanon, Libya, Mauritania, Morocco, Oman, Occupied Palestinian Territory, Pakistan, Qatar, Saudi Arabia, Somalia, Sudan, Syria, Tunisia, United Arab Emirates, and Yemen. The full search strategies for each database are provided in Supplementary S1 of the Supplementary Materials.

### 2.2. Selection Criteria

Included articles were: (1) Studies published in the English or French language. (2) Original articles with studies performed in the MENA region. (3) Studies reporting on serotype distribution of *S. pneumoniae* in the pre- and post-PCV era. (4) Studies reporting on the antibiotic susceptibility patterns of invasive *S. pneumoniae*.

Articles were excluded if they were not original papers (scientific meeting abstracts, research letters, letters to the editor, case reports and case series).

### 2.3. Study Selection Process

**Figure 1:**
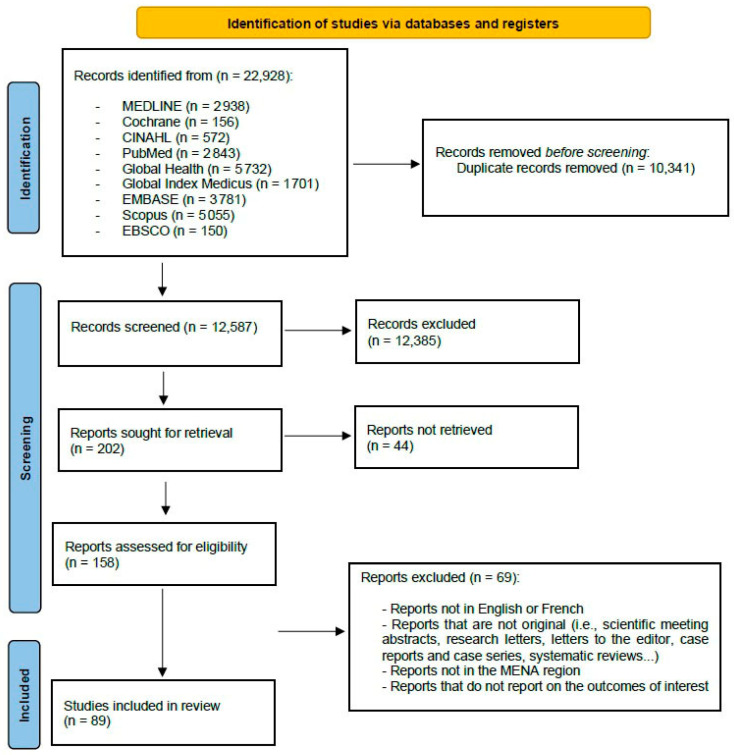
PRISMA 2020 flow diagram for the selection procedure for inclusion of articles from inception through 24 January 2024, in the final analysis of the review.

Six independent reviewers screened all titles and abstracts, followed by full-text review of eligible articles. Disagreements were resolved by discussion or by consulting a third reviewer. A PRISMA flow diagram (Figure 1 in Section 3) outlines the selection process and reasons for exclusion.

### 2.4. Data Extraction

Three teams of two independent reviewers extracted data using a standardized Excel sheet. Extracted variables included: study setting and year, study population characteristics, sample source and type, serotyping and antimicrobial susceptibility, in addition to data regarding PCV vaccination and vaccine-related serotypes. Any discrepancies were resolved by consensus or third-party review.

### 2.5. Risk of Bias Assessment

The quality of included observational studies was assessed using the Strengthening the Reporting of Observational Studies in Epidemiology (STROBE) checklist. Each article was independently appraised by two reviewers for completeness and transparency of reporting. The quality of reporting for each item was assessed as “Yes,” “No,” or “Not Applicable.” The percentage of satisfied applicable items was calculated using the formula: (number of items satisfied/total number of applicable items) × 100. Reporting quality was then categorized as follows: 0–25% (low), 26–50% (medium), 51–75% (good), and 76–100% (excellent).

### 2.6. Data Synthesis

Due to heterogeneity in study designs and outcome measures, a narrative synthesis approach was used. Serotype data were grouped according to vaccine formulations:

- PCV7 serotypes: 4, 6B, 9V, 14, 18C, 19F, 23F.
- PCV13 serotypes: 1, 3, 5, 6A, 7F, 19A.
- PCV15 serotypes: 22F, 33F.
- PCV20 serotypes: 10A, 15B, 8, 11A, and 12F.
- NVTs represented all other serotypes that were typed and reported but did not fit any of the above classifications.

## 3. Results

The flowchart in Figure 1 shows the selection procedure for the involved studies from inception through 24 January 2024. There were 22,928 articles retrieved for consideration from database searches. Of these, 10,341 papers were excluded due to duplication, and 12,587 articles were retained for the title and abstract assessment. Then, after reviewing the titles and abstracts, an additional 12,385 articles were excluded. After screening the remaining 158 articles for eligibility, 89 articles were found to satisfy the inclusion criteria.

### 3.1. Quality of Reporting of the Studies

Out of 89 studies, 49 had a good quality of reporting (55.1%) [18,19,20,21,22,23,24,25,26,27,28,29,30,31,32,33,34,35,36,37,38,39,40,41,42,43,44,45,46,47,48,49,50,51,52,53,54,55,56,57,58,59,60,61,62,63,64,65,66], while 28 had an excellent quality (31.5%) [5,6,67,68,69,70,71,72,73,74,75,76,77,78,79,80,81,82,83,84,85,86,87,88,89,90] (Table S1).

### 3.2. Overall Characteristics and Serotype Distribution of S. pneumoniae Isolates

The included studies (n = 89) were conducted in Algeria (n = 4) [18,19,20,21], Bahrain (n = 2) [67,90], Egypt (n = 3) [22,23,68], Iraq (n = 1) [24], Iran (n = 15) [25,26,27,28,29,30,31,32,33,34,35,36,37,69,70], Jordan (n = 1) [71], Kuwait (n = 6) [38,39,40,64,91,92], Lebanon (n = 6) [6,41,72,73,93,94], Morocco (n = 8) [5,42,43,44,63,82,88,95], Oman (n = 2) [45,83], Pakistan (n = 4) [46,47,48,75], Palestinian Territories (n = 1) [49], Qatar (n = 2) [50,96], Saudi Arabia (n = 14) [51,52,53,54,55,56,65,76,77,97,98,99,100,101], Tunisia (n = 7) [57,58,59,78,79,102,103], and Turkey (n = 12) [60,61,62,66,80,81,84,85,86,87,89,104] (Table S2). One study involved multiple countries (Algeria, Cyprus, Egypt, Jordan, Lebanon, Malta, Morocco, Tunisia and Turkey) [74]. No published papers were found from Afghanistan, Djibouti, Libya, Mauritania, Somalia, Sudan, Syria, and Yemen. Most of these studies were cross-sectional, performed at university hospitals, and included adult, pediatric, or combined populations. Almost 70% of the studies were performed prior to the introduction of any PCV into the national immunization program (NIP), 4 post PCV7 [38,50,55,104], 4 pre PCV13 [27,47,72,73], 4 post PCV 13 [83,85,86,87], 4 pre/post PCV13 [66,84,89,101], 3 pre/post implementation of both PCV13 and PCV10 within the same study period [5,88,95], 4 pre/post PCV10 [47,64,75,89], and 3 across multiple vaccine eras [6,64,89]. To note, one study in Bahrain did not specify the years over which data was collected, and hence we could not categorize it [90].

Only 59 articles reported the serotypes of invasive *S. pneumoniae* (Tables S3 and S4). Among a total of 5902 isolates recovered from various invasive samples, PCV7 serotypes 14, 19F, and 23F (10%, 10%, and 6% of the total, respectively) (Figure S1), as well as PCV13 serotypes 1 and 19A (7% and 5%, respectively) predominated. PCV15 and PCV 20 serotypes comprised 1% and 6%, respectively, of all isolates. As for NVTs, they cumulatively contributed 25% of the total pool.

### 3.3. Serotype Distribution of S. pneumoniae Isolates by Country

**Figure 2:**
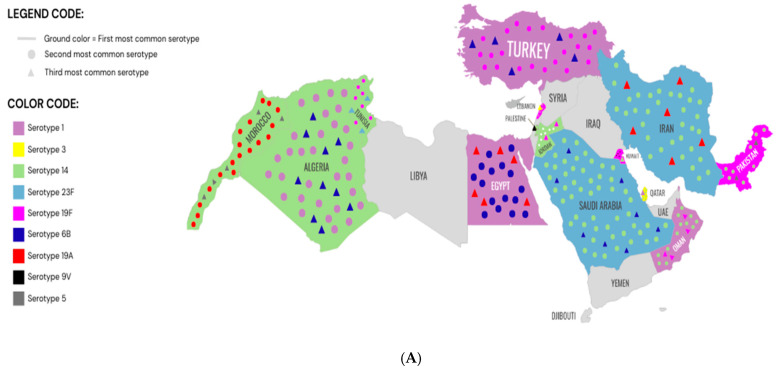

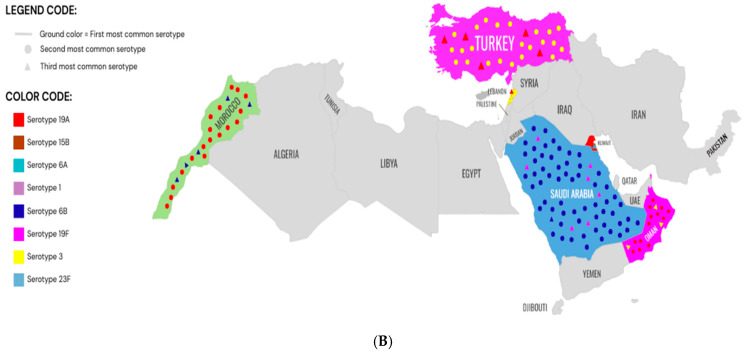
Maps of the Top 3 serotypes in each country in the MENA region during the pre-PCV13 era (**A**) and post-PCV13 era (**B**).

Figure 2 shows two maps of the Top 3 serotypes in each country in the MENA region during the pre-PCV13 era (A) and post-PCV13 era (B).

#### 3.3.1. Countries with No PCV Introduction into NIP

Until the date our review was conducted, PCVs were not yet incorporated within the NIP in Egypt, Iran, Jordan, and Palestinian Territories; their use in some of these counties has been limited to high-risk groups and/or physician preferences in the private sector. As for Algeria, our search did not yield eligible studies covering the period after April 2016, when PCV13 was introduced to the NIP. Similarly, studies from Tunisia were all prior to PCV10 introduction in April 2019. Scarce data of invasive *S. pneumoniae* serotypes was available from Egypt, Jordan, and Palestinian territories (Tables S3 and S4). Studies from Algeria, Iran, and Tunisia showed that PCV7 serotypes predominated in earlier years, but as years progressed PCV13 serotypes emerged. In Algeria, between 2001 and 2014, PCV7 serotypes 14, 19F, and 6B were among the prevalent serotypes [18,19,20,21]. Serotype 23F was initially common, but later replaced by PCV13 serotypes 1, 5, and 19A, which cumulatively contributed to 30% in the most recent data by Ziane et al. [21]. Similarly in Iran, serotype 19A started appearing as a common contributor (18%) [29] after serotypes 14 and 23F dominated earlier (24% and 18%, respectively) [25,27,28,29,31,32,33]. Non-typeable isolates (NTs) from these countries ranged between 5 and 20% [27,33]. As for Tunisia, recent data revealed the increase in serotype 1 to 23% [57], as well as serotype 3 (11%) and 6A (7.5%) [79]. On the contrary, NVTs showed a declining prevalence in Algeria from 26% between 2001 and 2010 [18] to 7.5% between 2010 and 2014 [21]; and in Iran from 50% [25] to 30% [28,30].

#### 3.3.2. Countries with Data Pre and Post PCV Introduction into NIP

**Figure 3:**
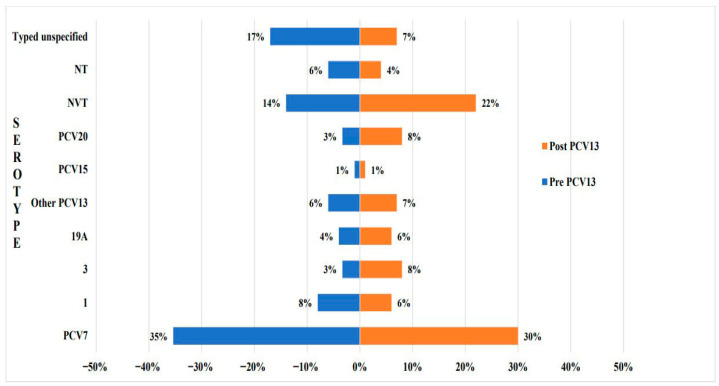
The impact of PCV13 implementation on invasive *S. pneumoniae* serotype distribution. PCV7 serotypes: 4, 6B, 9V, 14, 18C, 19F, 23F; Other PCV13 serotypes: 5, 6A, 7F; PCV15 serotypes: 22F, 33F; PCV20 serotypes: 10A, 15B, 8, 11A, and 12F. NVT: non-vaccine type, represented all other serotypes that were typed and reported, but are not among the PCV20 serotypes; NT: non-typeable; Typed but unspecified: serotypes that were typed but not reported, hence we could not classify them within any category.

Five countries had data on invasive IPD serotypes before and after the introduction of PCV(s) into their NIPs, with PCV13 being the latest (Tables S3–S5 and Table 1): Kuwait (n = 2), Lebanon (n = 2), Oman (n = 2), Saudi Arabia (n = 6), and Turkey (n = 7). Combined data from these countries yielded a total of 2105 invasive *S. pneumoniae* serotypes during pre PCV13 era [6,38,45,51,52,55,56,60,61,64,73,80,89,97], compared to only 799 isolates after PCV13 introduction [6,64,83,84,86,87,89,101] (Figure 3). In the pre-PCV13 era, PCV7 serotypes prevailed at 35%, followed by serotypes 1, 3, and 19A (15% combined). After PCV13 introduction, a modest 5% decrease in PCV7 serotypes was observed. Although serotype 1 declined by 2%, the overall proportion of PCV13 serotypes rose by 6%, largely due to a nearly threefold increase in serotype 3 and, to a lesser extent, a 2% increase in serotype 19A. Similarly, PCV20 serotypes almost tripled, while NVTs nearly doubled to 22%. Serotypes 22F and 33F remained stable.

As for initial PCV7 introduction within countries, retrieved data from Kuwait revealed that the most common serotypes among invasive strains in pre-PCV era were PCV7 serotypes 14, 23F, 9V, and 19F [39,64]. After PCV7 was added in August 2006, Mokaddas et al. noticed a modest reduction of around 20% of PCV7 serotypes across all ages [64] (Table 1 and Table S5), and a rise in PCV13 serotypes 1, 3, and 6A [64]. When differentiating between age groups, it was observed that serotypes 19F and 8 prevailed in children ≤ 5 years as well as adults > 50 years. Serotypes 19A, 6A, and 15B were additionally common among children; whereas serotypes 14, 3, and 1 were the most common in adults [38]. Later in August 2010, PCV7 was replaced by PCV13; which further reduced PCV7 serotypes infection by an overall 57% [64] with an increase in IPD due to serotype 19A, decrease in 6A, and disappearance of 1. PCV20 serotypes increased from 6% in pre PCV7 era to 24% post PCV13 era [39,64]. On the other hand, NVTs increased from 13% to 35%, with serotypes 33D, 20, 9L, 17F being the most frequent.

A similar patten was observed in Lebanon where PCV7 was introduced in 2006 in the private sector, followed by PCV10 and PCV13 in 2010. Later in January 2016, PCV13 was added to NIP using the 2 + 1 schedule (4, 6, and 12 months). The most common serotypes among all ages within 2005–2009 were 3, 6B, 19F, and 14 (cumulatively 38%) [6,72]. Later, serotype 6B was replaced by serotypes 1 and 19A (8% and 7%, respectively) (Table 1 and Table S5). PCV13 era witnessed a further decrease in PCV7 serotypes and dominance of PCV13 serotypes, particularly 1 and 3 (20% of 170 IPD cases) [6]. Moreover, a significant expansion of NVTs from 10% to 25% was observed; mainly due to serogroup 24 and serotype 16F. The increase in PCV20 serotypes was less significant, from 9% to 11%. When categorized by age, it was observed that in children ≤ 5 years of age, serotype 3 did not constitute a major contributor in IPD until PCV13 era. On the contrary, this serotype predominated in adults above 60 years of age along all periods, reaching its maximum contribution of 18% in PCV13 era.

Between 2002 and 2007, prior to PCV introduction in Oman, serotype 1 (15%) was the most common serotype followed by PCV7 serotypes 6B, 14, and 19F (12% each) [45]. PCV7 was incorporated into NIP in 2008, replaced by PCV10 in 2010, and later replaced by PCV13 in 2012. Among a total of 132 IPD cases, between 2014 and 2016, data by Al-Jardani et al. revealed the persistence of serotype 19F (7.5%) and replacement of serotype 1, along with PCV7 serotypes, by serotypes 3 and 19A (6% each) [83]. Notably, a major rise in NVTs from 3% to 56% occurred, particularly serogroups 11, 12, and 15 [45,83].

In Saudi Arabia, PCV7 was introduced in the private sector in 2006 before it became included in the NIP in 2008. Two studies among children ≤ 5 years of age were conducted by Shibl et al. at two time periods reflecting serotypes of invasive *S. pneumoniae* pre PCV7, and up to 4 years after PCV7 implementation [55,56]. In both studies the leading serotypes were 23F (13–19%) and 19F (9–13%). During the earlier period, PCV7 serotypes 14, 6B, and 18C were common, whereas serotypes 1, 5, and 7F prevailed later (25%). Between 2009 and 2012, during which PCV7 was replaced by PCV13 in 2010, there was persistence of serotypes 23F (23%) and 19F (12%) [101]. Additionally, serotypes 6B (14%) and 18C (10%) re-emerged whereas PCV13 serotypes declined (11%) [101]. The rate of NVTs remained relatively stable between the study periods, not exceeding 14%; with serotypes 15 and 23A (44% of NVTs) being the main contributors.

In our review, Turkey was the country with the largest number of studies reflecting multiple vaccine eras. PCV7 was the first pneumococcal vaccine to be incorporated into the NIP in 2009. Between 2008 and 2010, Ceyhan determined that serotypes 19F (19.3%), 6B (7.9%), 4 (6.9%), and 14 (5.9%) were the leading serotypes among children with IPD [104]. NVTs constituted 13% with serotypes 7A, 15, 15C, in addition to PCV20 serotype 8 individually corresponding to 2.5% of the total pool. In November 2011, PCV13 replaced PCV7; data during 2011–2012 revealed that serotype 19F continued to be prevalent with the emergence of serotype 3 (7.5%) [89]. These two serotypes remained prevalent during 2013–2014, with the appearance of serotype 14 again (9.0%). An increase in typed, but unspecified, serotypes to 27% was observed, whereas NVTs slightly decreased to 8%. A subsequent study, 4 to 7 years from PCV13 addition, revealed the further persistence of serotypes 19F (11.9%) and 3 (10.1%); whereas serotype 14 was replaced by serotype 1 (10.7%) [87]. Furthermore, there was an increase in PCV 20 serotypes to 14% occurred.

**Figure 4:**
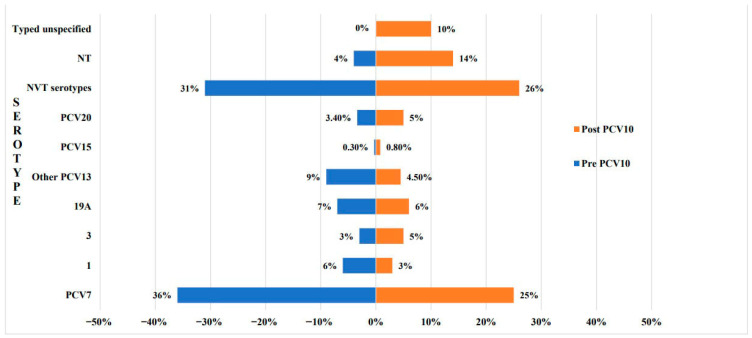
Comparison of invasive pneumococcal disease serotype prevalence pre and post PCV10 introduction. PCV7 serotypes: 4, 6B, 9V, 14, 18C, 19F, 23F; Other PCV13 serotypes: 5, 6A, 7F; PCV15 serotypes: 22F, 33F; PCV20 serotypes: 10A, 15B, 8, 11A, and 12F. NVT: non-vaccine type, represented all other serotypes that were typed and reported, but are not among the PCV20 serotypes; NT: non-typeable; Typed but unspecified: serotypes that were typed but not reported, hence we could not classify them within any category.

As for Morocco and Pakistan, PCV10 was the latest PCV to be added; with a pooled total of 585 invasive isolates before introduction [5,43,44,46,47,88] and 370 after [5,75,88,95] (Figure 4). In contrast to the previous countries, the decline in PCV7 serotypes was of greater magnitude, from 36% to 25%. Additionally, overall PCV13 serotypes decreased from 25% to 18.5%. Although serotype 3 increased from 3% to 5%, the proportion of serotype 1 decreased by half, while that of 19A was only 1%. PCV20 serotypes slightly increased from 3.4% to 5%, while NVTs decreased by 5%. Yet, a significant proportion of the post-PCV10 isolates were classified as either NT or typed but not specified serotypes (20% increase). As for serotypes 22F and 33F, their contribution remained relatively stable. To note, in Morocco, PCV13 was initially introduced in 2010 before it was replaced by PCV10 in 2012, which had variable impacts on children and adults. Among a total of 187 IPD cases in children ≤ 5 years of age, pre PCV-13 introduction, the most common serotypes were: 14 (13%), 5 (8%), 1 (8%), and 6B/19F/23F (6% each) [43]. Data shortly after the successive introduction of both vaccines highlighted the further dominance of serotypes 14 and 6B (31%), slight decrease in serotypes 1 and 5, whereas 19F and 19A were not detected [5]. As for NVTs, a decrease from 32% [43] to 22% [5] was noticed. Among adults, Nzoyikorera observed that PCV13 serotypes 19A and 3, as well as PCV20 serotype 8 prevailed initially (10%,10%, and 9%, respectively) [88]. In early and late post PCV phases serotypes 3 and 8 remained major contributors, but 6B and 23F (7% each) emerged in the early period then decreased. On the contrary, serotype 19A prevalence diminished between 2011 and 2014, then increased again to 7% between 2015 and 2019. Although the rate of NVTs was similar before and directly after vaccination, it almost doubled later (67%), mainly due to serotypes 9N, 17F, 33.

### 3.4. Antimicrobial Resistance to Penicillin, Macrolides, and Ceftriaxone/Cefotaxime

Penicillin, erythromycin, and ceftriaxone/cefotaxime were the most frequent antimicrobials tested in *S. pneumoniae* isolates in 61 studies (Table 2 and Table S6). Other antibiotics were not reported consistently, including trimethoprim/sulfamethoxazole, clindamycin, tetracycline, vancomycin, and carbapenems.

#### 3.4.1. Countries with No PCV Introduction into NIP

Resistance to penicillin was highest in Algeria and Tunisia reaching almost 70% [19,21,79]. In Iraq and Iran this rate ranged between 16% and 50% [24,27,31,35]. The scarce data from Bahrain, Egypt, and Jordan revealed penicillin non-susceptibility ranging between 14% and 33% [67,74]. As for erythromycin, non-susceptibility ranged from 23% in Egypt, to 50% in Algeria, but exceeded 70% in Iraq and Iran [20,24,27,31,67,74]. On the contrary, more than 90% of *S. pneumoniae* isolates within those countries were sensitive to ceftriaxone/cefotaxime [19,20,27,31].

#### 3.4.2. Impact of PCV Introduction on AMR

**Figure 5:**
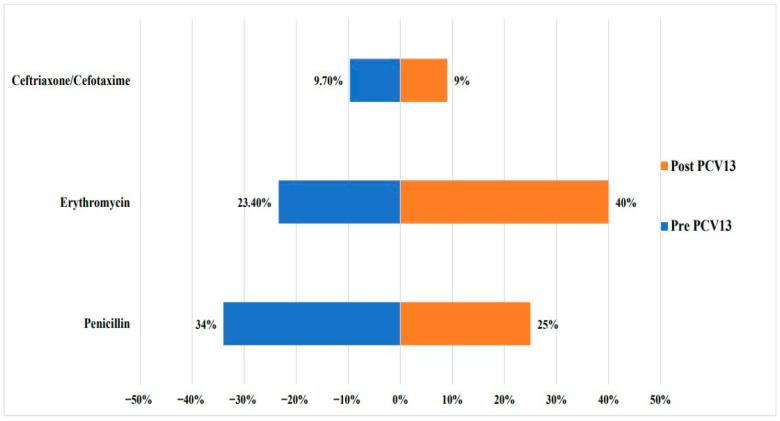
Proportion of non-susceptibility to penicillin, erythromycin, and ceftriaxone/cefotaxime among pre- and post-PCV13 invasive *S. pneumoniae* isolates.

Several countries demonstrated that vaccine serotypes 9V/9A,14, 19A, 19F, and 23F were more associated with penicillin resistance compared to other serotypes [6,19,21] (Table 3 and Table S7). Compared to serotype distribution, data reflecting AMR changes pre and post PCV13 introduction was very limited. Cumulative data from Kuwait, Lebanon, Morocco, Saudi Arabia and Turkey revealed a decrease of around 10% in penicillin resistance reaching 25% after PCV13 implementation [5,6,38,43,44,54,56,61,87,92,100,101,105] (Figure 5). On the contrary, a notable rise in erythromycin non-susceptibility, from 23% to 40%, was observed [5,6,43,44,54,56,61,100,101]. As for ceftriaxone/cefotaxime, the proportion remained relatively stable at around 9% between the two periods [6,43,44,87].

When examining trends within individual countries, a significant variability in penicillin resistance during pre PCV era, ranging between 2% and 45% [38,39,91,92], was evident in Kuwait. The only study including post PCV13 era, by Mokaddas et al., showed that initially penicillin non-susceptibility was 6.5%, increased to 7.3% post PCV7, and then to 9% in the period after PCV13 [64]. In Lebanon, 18.6% of invasive *S. pneumoniae* isolates were found to be resistant to penicillin in PCV7 era [6,72]. This rate decreased slightly to 15.4% in the post-PCV7/pre-PCV13 era; with a further significant decline to 8% in the PCV13 era [6]. In contrast, an initial elevation of erythromycin resistance from 28% to 37.5% occurred; followed by a decline to 27% in the PCV13 era. Susceptibility to ceftriaxone was highest compared to other antibiotics, increasing from 84% in PCV era to reach 100% in PCV13 era. Similarly in Morocco, penicillin non-susceptibility decreased from 35–37% to 24% after PCV13 implementation [5,43]; whereas that of erythromycin slightly increased from 17% to 22% [5,43]. Data from Saudi Arabia revealed that pre PCV, 20% to 54% of IPD specimens were penicillin resistant [53,54,56,99,100]. This ratio reached 36% in the only study reflecting post PCV13 period [101]. As for erythromycin resistance, the rate increased from 20–30% [54,56] to 77% [101]. Cefotaxime susceptibility remained high, though it slightly decreased from 93% to 90% [99,101]. Ceyhan et al. detected an increase in penicillin-resistant invasive *S. pneumoniae* in Turkey from 16.5% in 2008–2010 to 33.7% post-PCV7 implementation. After PCV13 replaced PCV7, the rate remained relatively stable at 32.9% [87,104]. In a study by Altun et al., 12.6% of isolates were erythromycin-resistant [61]. As for ceftriaxone, susceptibility reached 84.7% in the post-PCV13 era [87].

## 4. Discussion

This systematic review revealed a decrease in PCV7 serotypes following the introduction of either PCV10 or PCV13, with a more pronounced reduction observed in studies covering the post PCV10 era. This could be attributed to the fact that many of the pre-PCV13 isolates were retrieved in eras following PCV7 implementation [38,50,55,104]. As a result, major reductions in PCV7 serotypes had already occurred prior to PCV13 implementation, and the subsequent impact of PCV13 on these serotypes may have been underestimated compared to PCV10 implementing countries.

Although serotype 3 increased in both groups, yet more significantly post PCV13, serotype 19A declined only after PCV10. One plausible explanation for this unexpected finding is that most of the post-PCV10 data came from Morocco [5,88,95], where within the same study period PCV13 was introduced first and then replaced by PCV10 two years later. This transition may have confounded the ability to isolate the specific impact of PCV10. Moreover, the post PCV10 invasive serotypes reflected a period that extended only up to two years after the vaccine’s introduction, which may be insufficient to capture its full epidemiological effect. This may also have contributed to the observed decrease in NVTs, in contrast to their expansion post PCV13; in addition to the fact that nearly 20% of IPD isolates post PCV10 were either NTs or typed but unspecified.

Our findings align with those of a recent review by Ugrekhelidze et al., which determined that the decrease in proportion of PCV7 serotypes among children in MENA countries during the post-PCV era reached 50%; whereas PCV13 serotypes have remained prevalent at high levels (≥50%) [106]. Serotypes 3, 19A, and 19F predominated despite vaccination [106]. However, our observed impact is of lower magnitude, probably because it reflected changes across all age groups, not only children below 18 years of age. Analysis of data from underrepresented countries of WHO regions revealed more variability compared to us, where the proportion of PCV13 serotypes after the use of PCV10 or PCV13 ranged from 20% to 90% [107]. Reports from high income countries highlighted similar findings as well. The Active Bacterial Core surveillance data by the Centers for Disease Control and Prevention, USA, between 1998 and 2018, showed that most of the remaining PCV13-realted IPD was due to serotypes 3, 19A, and 19F [108]. Furthermore, among 30 high-income countries that already had introduced PCV10 or PCV13 into their NIP, serotypes 3 and 19A contributed to 11.4% and 13.3% of invasive *S. pneumoniae* cases, respectively, across all countries [109].

Several theories of the ongoing leading role of serotypes 3, 19A, and 19F in invasive disease, despite the widespread use of PCV13, have been proposed. Nasopharyngeal carriage data, mainly available in high income countries, reflected the persistence of these serotypes. This suggests that higher antibody concentrations might be necessary to protect against their carriage, which is a precursor for their dissemination [17,110,111]. Another possible cause of their reemergence, after a decline, in some countries is the development of a new clades that evade the immune response induced by the current PCVs [112,113,114]. Moreover, the unique structure of serotype 3 capsule may necessitate the activation of distinct immunological pathways in order to reach optimal protection [109,115,116]. Additionally, low vaccination rates, schedules lacking a booster dose, as well as transmission from unvaccinated older siblings to younger infants may contribute to this phenomenon [117,118,119,120].

As for PCV20 serotypes, data from high income countries, as well as under-represented regions, highlighted the rising prevalence of PCV20 serotypes, exceeding 30%. However, contrary to our categorization, in most of these studies PCV20 serotypes encompassed all 15 serotypes included in PCV15, combined with the additional 5 serotypes [16,107,109,121,122,123,124,125]. Conforming with our post PCV13 findings, NVTs expansion has been universally reported, alarmingly exceeding 60% in some European countries [107,108,121,122,123]. This has been reflected in nasopharyngeal carriage data as well; where carriage rate post PCV had been stable, or even higher compared to pre PCV era, due to increase in NVTs despite decrease in vaccine types [126,127].

The data on AMR in the MENA region is insufficient to conclude a general trend. Included countries showed a wide variability, with an increase in resistance after PCV implementation in some countries and decrease in others. This is consistent with a recent systematic review by Reyburn et al. assessing the impact of PCV10 or PCV13 on AMR in high and low-middle-income countries [12]. This could be induced by differences in baseline AMR, laboratory cut-off levels used, as well as vaccination rates [12]. Furthermore, overuse of antibiotics is an essential contributor. The rate of self-medication is high in the Middle East in general, surpassing 80% in some countries including Jordan, Syria, and United Arab Emirates [128]. In contrast, European countries, where access to over-the-counter antibiotics is tightly regulated, experience much lower rates ranging from just 1% to 4% [129]. Increasing empirical use of penicillins and macrolides, particularly, leads to increased resistance to these classes via different mechanisms: transfer of resistance genes, clonal expansion of an NVT global pneumococcal sequence cluster lineage associated with AMR, or expansion of an existing NVT within a resistant global pneumococcal sequence cluster lineage [123,130,131,132]. This mechanism has been suggested to explain the emergence of multi drug-resistant vaccine serotypes, such as 19A, after PCV7 introduction [12,123,132].

### Strengths and Limitations

The main strength of this review is the inclusion of 89 studies, spanning multiple countries in the MENA region, reflecting serotype distribution among different age groups and multiple vaccine eras. We compared patterns detected with those of high and low-middle-income countries where this data is available. However, certain countries (e.g., Afghanistan, Djibouti, Libya, Mauritania, Somalia, Sudan, Syria, Yemen) lacked published data, limiting the representativeness of the findings for the entire MENA region. Overall, the studies included in our review were heterogeneous in several aspects. Some countries had not implemented PCVs in their NIP until the end of the current review, which diluted the expected impact of PCV in the region. Even among studies reflecting pre and post vaccine eras, we detected differences between the PCV used and/or the sequential use of PCVs in a country; in addition to variability in the vaccination schedule followed. The number of post-PCV10/PCV13 implementation serotypes retrieved was significantly less than that obtained for the pre-PCV era, further hindering the opportunity to capture the exact impact. Even within the available serotypes, we might have overestimated the vaccine types because some reports only indicated group types instead of subtypes, which were then added to a vaccine type when applicable. Furthermore, most studies reflecting serotype distribution post-PCV, were conducted over a period not exceeding 4 years from initiation; even successive incorporation of more than one vaccine in a single study occurred. This is in contrast with the surveillance data reported from the USA and Europe, usually spanning a duration up to 8 years from introduction. Moreover, information regarding vaccination rates across countries and PCV eras was not extracted, which is a critical determinant of vaccine impact.

## 5. Conclusions

Data reflecting serotype distribution of invasive *S. pneumoniae* and the corresponding AMR in the MENA region is deficient on multiple levels. This information is crucial in forming decisions on higher valency vaccine introduction. From this review, we can conclude that PCV20, rather than PCV15, would provide better serotype coverage in the region. Yet, there is a need to conduct more studies across all countries to assess long-term trends that reveal the mature impact of PCVs. Improved surveillance should include nasopharyngeal carriages, since they are a precursor of pneumococcal infection. Furthermore, the application of metagenomics could help reveal the mechanisms behind the persistence and emergence of vaccine and non-vaccine types.

## Figures and Tables

**Table 1:** Serotype Distribution according to Pneumococcal Conjugate Vaccine (PCV) coverage by country and age groups in the MENA region (19 original studies up to 24 January 2024).

Author, Year	Country	Total Number of IPD Isolates with Documented Serotypes	Population	Period	Age Distribution	Number of Isolates /Age Group	PCV7 Serotypes	PCV13/Non-PCV7 Serotypes	PCV15 Serotypes	PCV20 Serotypes	Non- Typeable	Other Serotypes
Pre-PCV13 introduction												
[21]	Algeria	80	Pediatric	2010–2014 (pre-PCV7 introduction)	<1 y	44	20	19			1	35F (1), 35B (1), 24F (1), 20 (1)
					1–2 y	20	12	6			1	9N/9L (1)
					3–5 y	16	12	3				6C (1)
[22]	Egypt	99	All age groups	1977–1978 (pre-PCV7 introduction)	<1 y	24	1	5				2 (2), 6 (1), 7 (2); 9 (1); 12 (1); 18 (1), 20 (2); 33 (1); 34 (2); 35 (1); 36 (1); 45 (1); 46 (2)
					1–4 y	16	1	8				6 (2), 7A (1), 9 (1); 12 (1); 23 (1); 39 (1)
					5–9 y	12	2	7				9 (1);19 (1);46 (1)
					10–14 y	20		6				6 (2); 9 (1); 10 (1); 15 (1); 18 (1); 19 (2); 20 (1); 24 (1); 36 (1); 38 (1); 45 (2)
					15–34 y	17	1	7				2 (1); 9 (2); 9N (1); 10 (1); 12 (1); 20 (1); 29 (1); 29, 35, 42 (1)
					≥35 y	10		3		1		7 (1); 9N (2); 12 (1); 45 (1); 29, 42 (1)
[27]	Iran	53	Pediatric	2013–2016 (pre-PCV13 introduction)	0–3 months	9	6			1	1	15A (1)
					4–24 months	25	18	2			1	6 (2), 35B (1), 31 (1)
					25–60 months	19	9	4		3	1	15A (1), 34 (1)
[33]	Iran	19	Pediatrics	2016–2017 (post-PCV introduction)	≤1 y			1			1	3 & 5A (1); 3 & 11A (1)
					2–4 y		1	1		1	2	3 & 11A (2); 6A & 7C (1); 19F & 23B (1)
					5–10 y		1	1			1	3 & 11A (2); 7C & 14 (1)
[71]	Jordan	23	Pediatrics	2021–2022 (pre-PCV introduction)	≤6 m	12	8	3				28 (1)
					7 m–12 m	4	3	1				
					13 m–24 m	2		1				Other unspecified serotype (1)
					25 m–53 m	5	3	2				
[38]	Kuwait	129	All age groups	2006–2011 (Post PCV7 introduction)	<2 y	26	The predominant serotypes in children ≤5 years were 19F, 19A, 6A, 8 and 15B. However, the predominant serotypes in adults >50 years were 14, 3, 1, 19F and 8.				9	
					2–5 y	19						
					6–50 y	36						
					51–65 y	24						
					>65 y	24						
[47]	Pakistan	111	All age groups	2005–2013 (pre-and post-PCV13 introduction) (Pre PCV10 introduction)	0–59 months	85	20	12		3	5	6A/6B/6C (2), 9V/9A (3), 9N/9L (1), 10F/10C (1), 10F/10C/33 (1), 11A/11D (2), 12F/A/44/46 (6), 15B/15C (2), 18A/18B/18C/18F (13), 22A/22F (1), 23A (2), 23B (6), 33F/A/37 (1), 35B (2), 24A/B/F (1), 17 (1)
					5–15 y	9	3	1			1	7F/7A (1), 11A/11D (1), 18A/18B/18C/18F (1), 24A/B/F (1)
					18–70 y	17	7	4			1	13 (2), 15B/15C (1), 22A/22F (1), 38/25F/25A (1)
[50]	Qatar	134	All age groups	2005–2009 (Post PCV7/pre-PCV13 introduction)	<2 y	23	8	10			2	35B (2), 24F (1)
					2–5 y	28	14	7		1	2	9A (1), 12F/A/44/46 (1), 18 (1), 35B (1)
					6–64 y	58	16	22	2	2	7	6C (1), 7C (1), 12F/A/44/46 (2), 18 (1), 18F (1), 31 (1), 34 (2)
					>64 y	25	6	10			1	G (1),6A/6B (2), 7C (1), 12F/A/44/46 (1), 15A (1), 18F (1), 23A (1)
[52]	Saudi Arabia	71	All age groups	2000–2001 (pre-PCV7 introduction)	<15 y	51	7	3				6 (10), 7 (2), 15 (5), 18 (3), 19 (4), 23 (6)
					≥15 y	20		5				6 (1), 15 (3), 19 (6), 22 (2)
					Children (<2 y): 27 isolates included in the 51 isolates for children <15 y	27 isolates included in the 51 isolates for children <15 y	Serotypes 23 and 14 causing 6 (22%) cases. Serogroups/serotypes 3, 4, 6, 14 and 23 were the most common.					
[56]	Saudi Arabia	350	Pediatric	2000–2004 (pre-PCV13 pre-PCV7 introduction)	<2 y	159	132	7			11	7 (1), 11 (1), 15 (2), 22 (1), 23A (1), 23B (1), 24 (2)
					2–<5 y	106	66	8		1	15	7 (2), 11 (4), 12 (1), 22 (3), 23A (2), 23B (1), 24 (3)
					5 y	85	19	14		5	25	7 (3), 11 (1), 12 (6), 15 (4), 23A (3), 23B (3), 24 (2)
[58]	Tunisia	58	Pediatrics	1998–2004 (pre-PCV7 introduction)	<2 y	38	38					
					2–16 y	20	20					
[102]	Tunisia	73	All age groups	2012–2016 (Pre PCV introduction)	<2 y	25	18	4			1	9V/A (2)
					2–4 y	5	3	2				
					5–17 y	5	2	1		1	1	
					18–65 y	24	10	6				6C (1); 7C (1); 9V/A (2); 17F (1); 34 (1); 35B (1); 35F (1)
					>65 y	14	7	3			1	9V/A (1); 17F (1); 35B (1)
[60]	Turkey	27	Pediatric	2005–2007 (pre-PCV13 introduction)	<2 y	14	10	4				
					>2 y	13	2	9		1		18 (1)
**Post- PCV13 introduction**												
[85]	Turkey	252	Adults ≥18 years	2015–2018 (Post-PCV13 introduction)	<65 years			108	5	10		5 other unknown serotypes covered by PPV23; unknown number of NVT cases (35F, 15A, and 18F)
					≥ 65 years			58	2	8		Unknown number of NVT cases (35F, 15A, and 11C)
**Pre- and post-PCV13 introduction**												
[64]	Kuwait	212	All age groups	Pre-PCV7 vaccination 2003–2006	<2 y	9	5	1		1	1	33D (1)
					2–5 y	7	4	2		1		
					6–50 y	13	5	2		3	1	2 (2)
					51–65 y	25	13	6		1	1	7C (1), 9A (1), 22A (2)
					>65 y	9	6	1		1		9N (1)
				Post-PCV7 vaccination 2006–2010	<2 y	18	7	5		4		15C (1), 15F (1)
					2–5 y	14	8	4		1		15C (1)
					6–50 y	31	6	10		6		2 (1), 9L (1), 12B (1), 15C (1), 15F (2), 19C (1), 23A (2)
					51–65 y	21	9	6		2	2	15A (1), 18A (1)
					>65 y	23	6	7	1	5	1	15F (1), 20 (1), 33D (1)
				Post-PCV13 vaccination 2010–2013	<2 y	4	1	1		1	1	
					2–5 y	6		2		1	1	12B (1), 20 (1)
					6–50 y	12		3		2		9L (2), 17F (1), 20 (1), 33D (3)
					51–65 y	9	1	2		5		15F (1)
					>65 y	11		2	1	2		15A (1), 17F (1), 20 (1), 23A (1), 33A (1)
[6]	Lebanon	543	All age groups	2005–2009 (PCV7 era)	≤5 y	68	38	13	2	3	6	9N (1); 16F (1); 24F (1); 28A (1); 31 (1); 42 (1)
					6–60 y	48	18	12	3	6	2	9N (2); 15A/A5F (2); 16F (1); 29 (1); 38/25 (1)
					>60 y	56	19	18	7	6	1	9N (2); 16F (1); 29 (1); 34 (1)
				2010–2015 (post-PCV7/pre-PCV13 era)	≤5 y	76	34	24	1	5	2	2 (3); 9N (1); 10F/10C/33C (2); 16F (1); 23A (1); 24F (1); 35F/47 (1)
					6–60 y	64	21	24		4		2 (1); 6C (1); 9N (1); 10B (1); 13 (2); 15A/15F (1); 16F (1); 17F (1); 21 (2); 23A (2); 35B (1); 38/25 (1)
					>60 y	61	21	20	5	1	3	2 (1); 6C (1); 9N (1); 10B (1); 16F (2); 20 (1); 23A (1); 31 (1); 38/25 (1); 39 (1)
				2016–2020 (PCV13 era)	≤5 y	63	13	20	2	5	9	9N (2); 16F (1); 23A (2); 23B (1); 24F (5); 34 (1); 35B (1); 35F/47 (1)
					6–60 y	52	6	15	1	8	2	7C/7B/40F (2); 9N (1); 13 (1); 16F (1); 17F (1); 23A (1); 23B (2); 24F (4); 31 (2); 34 (2); 35A/35C/42 (2); 35F/47 (1)
					>60 y	55	12	18	4	6	6	2 (1); 9N (1); 10F/10C/33C (1); 15A/15F (1); 23A (1); 23B (2); 24F (1); 31 (1)
[5]	Morocco	136	Pediatrics	Pre-PCV13 introduction (2007–2010)	≤2 y	79	41	26	1	1	6	11A/11E (1), 18F (1), 10F (1), 24F (1)
					>2–5 y	12	6	5		1		
				Post-PCV13 introduction and PCV10 (2011–2014)	≤2 y	32	11	5		2	6	2 (3); 15A (1), 18F (2), 10F (1), 7A (1)
					>2–5 y	13	4	5			3	23B (1)
[88]	Morocco	239	Adults	2007–2010 (Pre-vaccine period)	15–59 y	71	14	19		10		2 (1); 7A (1); 7C (1); 10 (2); 23A (2); 34 (3); NVT (18)
					≥60 years	15	3	6	1	2		9N (1)|; 20 (1); NVT (1)
				2011–2014 (Early post-vaccine period)	15–59 y	57	13	12	1	6		7A (2); 10 (1); 11 (2); 17F (2); 33 (1); 34 (1); 35F (1); NVT (15)
					≥60 years	26	5	9		5		17F (1); NVT (6)
				2015–2019 (Mature post-vaccine period)	15–59 y	58	2	13	1	4		9N (1); 11 (1); 17F (3); 23A (1); 33 (3); 34 (1); NVT (28)
					≥60 years	12	2			1		9N (1); 23A (1); NVT (7)
[89]	Turkey	335	Pediatric	2008–2010 (pre-PCV7 introduction)	≤5 y	146	84	29		2	6	2 (2), 7A (1), 10 (1), 15 (2), 15C (2), 16F (1), 23A (1), 15 other serotypes
					≥5–≤18 y	56	12	9		3	7	6 (1), 7A (4), 10 (1), 15 (3), 15C (3), 17F (1), 12 other serotypes
				2011–2012 (Post-PCV7/During PCV13 introduction)	≤5 y	31	15	7				15C (1), 23A (1), 7 other serotypes
					≥5–≤18 y	36	7	9		1	2	10 (1), 15 (2), 15C (2), 23A (1), 11 other serotypes
				2013–2014 (post-PCV13 introduction)	≤5 y	38	14	9		1	4	2 (1), 9 other serotypes
					≥5–≤18 y	28	6	7		1	3	10 (1), 15C (1), 9 other serotypes

Background colors were used to indicate the pneumococcal vaccine (PCV) coverage period and to distinguish the respective studies.

**Table 2:** Antimicrobial susceptibility among IPD cases in the MENA region by country (61 original studies up to 24 January 2024).

Author, Year	Country	Total Number of Tested Invasive Isolates	Specific Classification	Penicillin			Erythromycin			TMP/SMX			Ceftriaxone			Clindamycin		
				S	I	R	S	I	R	S	I	R	S	I	R	S	I	R
Pre-PCV13 introduction																		
[19]	Algeria	100	Blood (N = 22)		46	11			22			51						
			CSF (N = 75)															
			Pleural fluid (N = 3)															
			<17 y (N = 46)			37												
			≥18 y (N = 54)		16	4												
[20]	Algeria	97		49	10	38	46											
[21]	Algeria	80	Meningitis (N = 39)			26												
			Non-Meningitis (N = 25)			20												
[74]	Algeria		Tested isolates: 216 for penicillin 181 for erythromycin			Proportion 41 (35–48) CI95			19 (14–26)									
	Egypt		Tested isolates: 347 for penicillin 317 for erythromycin			25 (21–30)			23 (19–28)									
	Jordan		Tested isolates: 57 for penicillin 46 for erythromycin			33 (22–47)			24 (13–39)									
	Lebanon		Tested isolates: 16 for penicillin 16 for erythromycin			50 (26–74)			25 (8–53)									
	Morocco		Tested isolates: 110 for penicillin 91 for erythromycin			16 (10–25)			12 (6–21)									
	Tunisia		Tested isolates: 117 for penicillin 116 for erythromycin			29 (21–38)			33 (25–42)									
	Turkey		Tested isolates: 369 for penicillin 342 for erythromycin			20 (16–25)			9 (6–12)									
[67]	Bahrain	371	Blood, CSF, and other invasive body fluids			52			42									
[68]	Egypt	52			12	40	52			39	13							
[30]	Iran	51	2014–2015						10			4						8
			2015–2016						11			11						14
			2016–2017						10			14						9
			2017–2018						14			14						13
			Blood (PNSP isolates)						45			43						44
[35]	Iran	28	Blood (N = 10)	3	1	6												
			CSF (N = 15)	7	1	7												
			Pleural Fluid (N = 3)	2		1												
[27]	Iran	53	Meningitis (N = 32)	27		5							30	0	2			
			Non-meningitis (N = 21)	15	2	4	5	1	15	7	2	12	19	0	2			
[33]	Iran	34	Blood (N = 13)			8												
			CSF (N = 15)			4												
			BAL (N = 6)															
[36]	Iran	4	BAL (N = 2)	1	1				2	1	1			1	1			
			Blood (N = 1)	1					1			1			1			
			CSF (N = 1)	1					1		1				1			
[37]	Iran	4	CSF (meningitis cases) (N = 4)			2												
[31]	Iran	44	Blood			7			32			38						19
[24]	Iraq	18	CSF (N = 18)	8/17 (47%)		9/17 (53%)	2/12 (16.7%)		10/12 (83%)	2/18 (11%)		16/18 (89%)	2/15 (13%)		13/15 (87%)	7/17 (41%)		10/17 (59%)
[91]	Kuwait	24	Blood			9												
[92]	Kuwait	49	Blood:25 isolates			Among the 11 isolates PRSP: 11% blood, 9% ET secretion, 4% CSF												
			CSF:7 isolates															
			Other invasive isolates:17 isolates															
[40]	Kuwait	122	Bronchial aspirates (N = 3)		1	1			1		1	1						
			Pleural Fluid (N = 2)		1	1		2			1							
			Tracheal secretions (N = 46)		30	5			17		5	35						
			Blood (N = 68)		30	2		1	29		6	8		20	6			
			CSF (N = 3)		3				2		1	2						
[39]	Kuwait	44	Blood (N = 42)			19												
			CSF (N = 2)			2												
[38]	Kuwait	129	Blood (N = 116)			0												
			CSF (N = 13)			2												
[93]	Lebanon	24	Blood:17 isolates	8	8	1												
			CSF:7 isolates	4	3	0												
[41]	Lebanon	27	Blood (N = 20)	11	5	4												
			CSF (N = 5)	2	3													
			Abscess (N = 1)		1													
			Pleural Fluid (N = 1)		1													
[94]	Lebanon	22	Blood (N = 16)	6	10	0												
			CSF (N = 6)	2	3	1												
[73]	Lebanon	37		25	10	2												
[42]	Morocco	531	All ages (N = 531)			105												
			Children (N = 252) (age ≤ 14 y)			75												
			Adults (N = 279)			30												
[44]	Morocco	24				15			4			8	24					
[43]	Morocco	187	1994–2001 (pre-PCV7 introduction)		19			15						6				
			2006–2010 (pre-PCV13 introduction)		50			17						7				
[45]	Oman	34	Blood (N = 32)			9												
			CSF (N = 2)			2												
[46]	Pakistan	87			8						54	27						
[48]	Pakistan	267	CSF (N = 267)															
			Pre-2008 (N = 209)	183	20	6												
			Post-2008 (N = 58)	37		21												
[49]	Palestinian Territories	120	Blood	120			82	5	33	46	21	53						
[50]	Qatar	118		60	40	12	90	1	27	39	12	67						
[98]	Saudi Arabia	52	Blood (N = 49)			16												
			CSF (N = 3)	3														
[76]	Saudi Arabia	27	Blood (N = 22)		5			2										
			CSF (N = 3)		1			1										
			Joint Fluid (N = 2)		0			0										
[77]	Saudi Arabia	49				10			4									5
[99]	Saudi Arabia	172		82	88								152/164 (93%)	12/164 (7%)				
[54]	Saudi Arabia	62	Blood (N = 58)	31	22	5	46		12	35	1	22						
			CSF (N = 4)															
[51]	Saudi Arabia	51	Blood (N = 27)			21												
			CSF (N = 24)			16												
[100]	Saudi Arabia	107	Blood and CSF	85	4	18	8	4	15									
[53]	Saudi Arabia	50	Blood	29	17	4						19						
[56]	Saudi Arabia	350	Blood (N = 287)	161	147	42			91									
			CSF and other (N = 63)															
[58]	Tunisia	106	Blood (N = 40)			LR: 17, HR: 4												
			CSF (N = 52)			LR: 20, HR: 1												
			Other invasive samples (joints, pleural and intraabdominal) (N = 14)			LR: 10, HR: 1												
[59]	Tunisia	200	Blood (N = 73)			LLR:25 HLR:14												
			CSF (N = 99)			LLR: 34 HLR: 3												
			Pleural Punction, arthritis, osteomyelitis (N = 28)			LLR: 12 HLR:2												
[78]	Tunisia	9	Blood						6			6						5
[57]	Tunisia	108				25												
[79]	Tunisia	106				72												
			Meningococcal isolates (n = 31)			21			17									
			Non-meningococcal isolates (n = 75)			51												
[62]	Turkey	124	Blood (N = 84)			84			82			80						
			CSF (N = 40)			40			40			38						
[81]	Turkey	218	Blood (N = 91)	64	24	3												
			CSF (N = 59)	44	14	1												
			Pleural Fluid (N = 23)	17	6	0												
			BAL (N = 17)	12	4	1												
			Tracheal Aspirate (N = 20)	13	5	2												
			Peritoneal fluid (N = 8)	6	2	0												
[80]	Turkey	332	<3 y (N = 64)	2 had MDR for Penicillin G + bactrim//1 had MDR for Penicillin G + Bactrim + erythromycin+ chloramphenicol///1 had MDR for Penicillin G + Bactrim + chloremphenicol														
			≥3 y (N = 268)	10 had MDR for Penicillin G and Bactrim//3 had MDR for penicillin G + bactrim + erythromycin///1 had MDR for Penicillin G + cefttriaxone														
[61]	Turkey	182	CSF (N = 32) (15 adults, 17 children)			16 (7 adults, 9 children)												
			Blood (N = 150)															
			Total (N = 182)					1 (pediatric patient)	22 (13 adults, 9 children)									14
**Post-PCV13 introduction**																		
[63]	Morocco	65	CSF (N = 65)	50		15	55		8	42	6	12	62	2	1			
[83]	Oman	132	Meningitis (N = 23)	14	0	9	95	3	34	89	8	35	22	1	0	110		22
			Non-meningitis (N = 109)	23	0	0							108	1	0			
[101]	Saudi Arabia	78	Meningitis (N = 32)	0	32	0												
			Non-meningitis (N = 46)	46	0	0												
			All cases						60			78						
[84]	Turkey	33	Meningitis			16									3			
			Non-meningitis			1									0			
[86]	Turkey	110		Oral penicillin (5), Penicillin parenteral non-meningitidis (78) Penicillin parenteral meningitidis (2)	Oral penicillin (34), Penicillin parenteral non-meningitidis (17)	Oral penicillin (56), Penicillin parenteral meningitidis (13)										8	0	102
[87]	Turkey	167				55							141					
**Pre- and post-PCV13 introduction**																		
[6]	Lebanon	537	2005–2009 (PCV7 era)															
			≤5 y	44	6	9	42		26	17	5	41	45	8	5	7		3
			6–60 y	39	2	6	39		11	16	6	25	43	2	2	1		2
			>60 y	44	4	2	45		12	18	10	25	45	6	2	8		5
			2010–2015 (Post-PCV7/pre-PCV13 era)															
			≤5 y	43	4	9	47	1	33	26	5	31	43	5	1	30	1	12
			6–60 y	44	1	5	41	2	19	26	3	30	42	1	1	28	0	11
			>60 y	45	3	2	42	0	23	32	1	26	41	1	2	23	1	6
			2016–2020 (PCV13 era)															
			≤5 y	43		7	38	2	16	25	4	28	47	1		42	2	14
			6–60 y	40		1	35	4	9	22	3	23	39			38	1	9
			>60 y	45			39		11	27	5	18	45			43		7
[5]	Morocco	136	Period 1 (pre-PCV13 implementation): ≤2 y (N = 79)		40			13				31						
			Period 1 (pre-PCV13 implementation): 2 y–<5 y (N = 12)		5			3				4						
			period 2 (post-PCV13 implantation): ≤2 y (N = 32)		7			8				2						
			Period 2 (post-PCV13 implementation):2 y–<5 y (N = 13)		4			2				2						
[88]	Morocco	239	2007–2010 (Pre-vaccine period)															
			15–59 y		17			5						1				
			≥60 years		2			1										
			2011–2014 (Early post-vaccine period)															
			15–59 y		5			6						1				
			≥60 years		2			2										
			2015–2019 (Mature post-vaccine period)															
			15–59 y		11			11						2				
			≥60 years		2			1										
[65]	Saudi Arabia	208	Period 1 (2006–2008) (N = 76) (Before the revision of breakpoints for meningitis, non-meningitis intravenous and oral administration)		47/72	5/72						15/76						
			Period 2 (2008–2012) (N = 132) (After the revision of breakpoints for meningitis, non-meningitis intravenous and oral administration)		Oral penicillin (76/115) Penicillin non-meningitis (1/125) Penicillin meningitis (0/120)	Oral penicillin (10/115) Penicillin non-meningitis (2/125) Penicillin meningitis (90/120)						93/132						
[89]	Turkey	335	2008–2010 (N = 202)			68												
			2011–2014 (N = 133)			22												
**Unspecified period**																		
[90]	Kingdom of Bahrain	22	Blood (N = 21)			4			9									
			CSF (N = 1)			1			1									

Background colors were used to indicate the pneumococcal vaccine (PCV) coverage period and to distinguish the respective studies.

**Table 3:** Antimicrobial susceptibility among IPD cases in the MENA region by country and PCV/non-PCV serotypes (14 original studies up to 24 January 2024).

Author, Year	Country	Total Number of Isolates	ATB	Antimicrobial Sensitivity	PCV7 Serotypes	PCV13/non-PCV7 Serotypes	PCV15 Serotype 33F	PCV20 Serotypes	Non-Typeable	Other Serotypes (N)
Pre-PCV13 introduction										
[19]	Algeria	100	Penicillin	R (N = 43)	35	8		2		
			Erythromycin	R (N = 16)	12	3		1		
[23]	Egypt	205	Penicillin	I (N = 97)	43	26		3	10	9A (1), 10B (1), 15A (4), 16F (1), 20 (2), 22A (2), 23B (1), 35 (2), Pool C (2), Pool E (1), Pool G (1), Pool I (2)
				R (N = 3)	3					
			Erythromycin	I and R (N = 26)	12	6			1	2 (1), 18A (1), 20 (1), Pool C (2), Pool H (1), Pool I (1)
			TMP/SMX	I and R (N = 149)	49	41		4	8	2 (2), 7A (1), 7B (1), 7C (1), 9A (2), 10B (1), 10F (1), 11C (1), 15A (3), 16F (5), 17F (1), 18A (1), 20 (2), 22A (1), 23B (4), 33C (1), 35 (2), 37 (1), Pool C (2), Pool D (2), Pool E (2), Pool F (1), Pool G (1), Pool H (1), Pool I (7)
			Ceftriaxone	I (N = 10)	8			1		
				R (N = 3)	3					
			Chloramphenicol	I and R (N = 21)	8	3			2	2 (2), 7A (1), 15A (1), 31 (2), Pool I (2)
			Tetracyclin	I and R (N = 109)	34	36		5	5	2 (3), 7A (1), 10B (1), 12A (1), 15A (1), 15C (1), 15F (1), 17F (1), 18A (1), 20 (3), 23B (4), 31 (1), Pool C (3), Pool D (1), Pool E (2), Pool H (1), Pool I (3)
[30]	Iran	51	Chloramphenicol	R (N = 29)	11	8			1	6A/B (5); 15A (1); 15B/C (3)
			TMP/SMX	R (N = 36)	15	9			1	6A/B (7); 15B/C (4)
			Clindamycin	R (N = 36)	14	9			1	6A/B (7); 15A (1); 15B/C (4)
			Erythromycin	R (N = 45)	14	17			1	6A/B (8); 15A (1); 15B/C (4)
			Oxacillin	R (N = 18)	10	4			1	6A/B (1); 15A (1); 15B/C (1)
			Cefotaxime	R (N = 6)	2	4			0	
			Tetracycline	R (N = 43)	13	17			1	6A/B (7); 15A (1); 15B/C (4)
[34]	Iran	53 (All PNSP)	Ceftriaxone	I (N = 19)	16					6A/6B (1); NVT (2)
				R (N = 14)	9	4				NVT (1)
			Cefotaxime	I (N = 13)	10	1				NVT (2)
				R (N = 16)	11	4				NVT (1)
[39]	Kuwait	43	Penicillin	S (n = 16)	3	9		1		11C (1), 16F (1), 17F (1)
				I (N = 22)	14	3		1		9A (1), 15A (3)
				R (N = 5)	5	0		0		
[38]	Kuwait	129	Penicillin	R (N = 2)	1	1				
[43]	Morocco	187	Penicillin	I and R (N = 66)	41	10		1	3	2 (1), 7 (2), 9 (1), 19 (3), 23 (4)
[45]	Oman	54	Penicillin	R (N = 11)	7	1		1	1	9A (1)
[46]	Pakistan	87	Chloramphenicol	R (N = 14)	9	5				
			Tetracyclin	R (N = 29)	18	1				Serogroup 16 (10)
			MDR		7 isolates with serotype 19F (tetracycline and penicillin)/4 (Chloramphenicol and tetracyclin)	1isolate with serotype 5 (co-trimoxazole and tetracyclin); 1 isolate with serotype 19A (co-trimoxazole and penicillin and tetracycline)/9 (chloramphenicol and tetracycline)				serotype 31: 1 MDR to Chloramphenicol and tetracyclin/12 MDR to co-trimoxazole, chloromphenicol and tetracycline; 15C (4 MDR to chloramphenicol and tetracycline); Serogroup 16 (2 MDR- chloramphenicol and tetracyclin)
[49]	Palestinian Territories	120	Penicillin	S (N = 120)	44	32	2	6	3	6A/B (17), 16F (2), Sg18 (5), 17F (1), 35B (3), 38F (1)
			Erythromycin	S (N = 82)	26	27	1	5	3	6A/B (8), 16F (2), Sg18 (5), 17F (1), 35B (3), 38F (1)
				I (N = 5)	1	3				6A/B (1)
				R (N = 33)	17	6	1	1		6A/B (8)
			TMP/SMX	S (N = 46)	14	6		2	3	6A/B (9), 16F (2), Sg18 (5), 17F (1), 35B (3), 38F (1)
				I (N = 21)	4	12		3		6A/B (2)
				R (N = 53)	26	18	2	1		6A/B (6)
			Vancomycin	S (N = 120)	44	32	2	6	3	6A/B (17), 16F (2), Sg18 (5), 17F (1),35B (3), 38F (1)
			Cefotaxime	S (N = 120)	44	32	2	6	3	6A/B (17), 16F (2), Sg18 (5), 17F (1),35B (3), 38F (1)
			Ofloxacin	S (N = 118)	42	32	2	6	3	6A/B (17), 16F (2), Sg18 (5), 17F (1),35B (3), 38F (1)
				I (N = 2)	2					
[56]	Saudi Arabia	350	Penicillin	S (N = 161)	77	16		6	25	Serogroup 7 (6), serogroup 11 (6), serogroup 12 (7), serogroup 15 (3), serogroup 22 (4), 23A (4), 23B (2), 24 (5)
				I (N = 147)	110	4			1	serogroup 15 (2), 23A (1), 23B (2), 24 (2)
				R (N = 42)	30	8			1	serogroup 15 (1), 23A (1), 23B (1)
			Erythromycin	R (N = 91)	70	10			5	serogroup 15 (3), 23B (1), 24 (2)
			Cefotaxime	R (N = 23)	16	4			0	23A (1)
[79]	Tunisia	106	Penicillin	S (N = 35)	11	15				7C (1); 9N (1), 13 (1); 16F (2); 17F (2); 24F (1); 34 (1)
				I (N = 59)	38	11				6C (1); 9A (2); 9N (2); 17F (1); 35B (3); 35F (1)
				R (N = 12)	10	1				9A (1)
			Cefotaxime	S (N = 89)	44	26				6C (1); 7C (1); 9A (3); 9N (3); 13 (1); 16F (2); 17F (3); 24F (1); 34 (1); 35B (2); 35F (1)
				I (N = 17)	15	1				35B (1)
			Tetracycline	S (N = 66)	34	19				7C (1); 9N (1); 13 (1); 16F (2); 17F (3); 34 (1); 35B (3); 35F (1)
				I (N = 5)	3	2				
				R (N = 35)	22	6				6C (1); 9A (3); 9N (2); 24F (1)
			Erythromycin	S (N = 39)	16	13				7C (1); 9N (1); 13 (1); 16F (2); 17F (2); 34 (1); 35B (1); 35F (1)
				I (N = 1)		1				
				R (N = 66)	43	13				6C (1); 9A (3); 9N (2); 17F (1); 24F (1); 35B (2)
**Pre- and post-PCV13 introduction**										
[6]	Lebanon	542	Penicillin	S (N = 387)	105	124	21	31	19	2 (4); 6C (2); 7C/7B/40F (2); 9N (7); 10B (2); 10F/10C/33C (2); 13 (3); 15A/15F (4); 16F (7); 17F (2); 20 (2); 21 (2); 23A (5); 23B (5); 23F (11); 24F (9); 28A (1); 29 (2); 31 (5); 34 (4); 35A/35C/42 (1); 35F/47 (2); 38/25 (2); 42 (1);
				I (N = 20)	17			1		2 (1); 9N (1)
				R (N = 43)	24	4		4		16F (1); 23F (3); 24F (1); 35B (2); 35F/47 (1); 38/25 (1); 9N (2);
			Ceftriaxone	S (N = 384)	111	127	19	35	18	2 (3); 6C (1); 7C/7B/40F (1); 9N (8); 10B (2); 13 (3); 15A/15F (4); 16F (7); 17F (1); 20 (2); 21 (2); 23A (5); 23B (5); 24F (9); 28A (1); 29 (2); 31 (5); 34 (4); 35A/35C/42 (1); 35B (2); 35F/47 (3); 38/25 (3)
				I (N = 23)	17	2		1	2	9N (1)
				R (N = 13)	12			1		
			Tetracycline	S (N = 349)	94	120	18	31	19	2 (5); 6C (2); 7C/7B/40F (2); 9N (7); 10B (1); 10F/10C/33C (2); 13 (3); 15A/15F (3); 16F (8); 17F (1); 20 (1); 21 (2); 23A (3); 23B (5); 24F (5); 28A (1); 29 (2); 31 (5); 34 (2); 35B (2); 35F/47 (3); 38/25 (1); 42 (1)
				I (N = 22)	7	9	2	2		9N (2)
				R (N = 125)	66	25	1	9	6	2 (1); 10F/10C/33C (1); 15A/15F (1); 20 (1); 23A (2); 24F (6); 34 (2); 35A/35C/42 (1); 38/25 (1); 9N (2)
			Erythromycin	S (N = 358)	93	125	21	34	16	2 (4); 6C (1); 7C/7B/40F (2); 9N (10); 10B (2); 10F/10C/33C (1); 13 (4); 15A/15F (3); 16F (7); 17F (1); 20 (2); 21 (2); 23A (3); 23B (5); 24F (3); 28A (1); 29 (2); 31 (5); 34 (3); 35B (2); 35F/47 (3); 38/25 (2); 42 (1)
				I (N = 8)	1	3			1	2 (1); 24F (1); 35A/35C/42 (1)
				R (N = 153)	80	34	3	9	10	10F/10C/33C (2); 15A/15F (1); 16F (2); 2 (1); 23A (1); 24F (7); 38/25 (1); 6C (1); 9N (1)
			Chloramphenicol	S (N = 409)	135	127	15	37	22	2 (5); 6C (1); 7C/7B/40F (2); 9N (9); 10B (2); 10F/10C/33C (2); 13 (4); 15A/15F (3); 16F (7); 17F (1); 20 (1); 21 (2); 23A (4); 23B (4); 24F (10); 29 (2); 31 (5); 34 (1); 35A/35C/42 (1); 35B (2); 35F/47 (3); 38/25 (2)
				R (N = 40)	13	10	4	1	1	2 (1); 6C (1); 16F (2); 23A (1); 23B (1); 34 (3); 9N (2)
			Clindamycin	S (N = 212)	52	71	9	14	16	2 (4); 6C (1); 7C/7B/40F (2); 9N (6); 10B (2); 10F/10C/33C (1); 13 (3); 15A/15F (1); 16F (6); 17F (1); 20 (1); 21 (2); 23A (3); 23B (4); 24F (3); 31 (4); 34 (1); 35B (2); 35F/47 (2); 38/25 (1)
				I (N = 5)	2	1				23B (1); 34 (1)
				R (N = 64)	21	18		7	4	2 (2); 10F/10C/33C (1); 15A/15F (1); 23A (1); 24F (7); 34 (1); 35A/35C/42 (1)
			TMP/SMX	S (N = 201)	41	77	14	12	14	2 (4); 7C/7B/40F (2); 9N (2); 10B (1); 10F/10C/33C (2); 13 (2); 15A/15F (3); 16F (2); 17F (1); 23A (2); 23B (2); 24F (3); 28A (1); 29 (2); 31 (4); 34 (1); 35A/35C/42 (1); 35B (2); 35F/47 (3); 38/25 (3)
				I (N = 43)	11	18	6	4		24F (2); 34 (1); 9N (1)
				R (N = 242)	109	58	1	22	10	2 (2); 6C (2); 9N (8); 10B (1); 10F/10C/33C (1); 13 (2); 15A/15F (1); 16F (5); 20 (2); 21 (2); 23A (3); 23B (3); 24F (6); 31 (1); 34 (2); 42 (1)
			Levofloxacin	S (N = 448)	149	143	18	37	24	2 (5); 6C (2); 7C/7B/40F (2); 9N (8); 10B (2); 10F/10C/33C (3); 13 (3); 15A/15F (4); 16F (7); 17F (1); 20 (1); 21 (2); 23A (4); 23B (5); 24F (10); 28A (1); 29 (1); 31 (5); 34 (3); 35A/35C/42 (1); 35B (2); 35F/47 (3); 38/25 (2)
				I (N = 3)	1					16F (2)
				R (N = 14)	3	5		3	1	23A (1); 24F (1)
			Vancomycin	S (N = 504)	169	158	24	38	24	2 (6); 6C (2); 7C/7B/40F (2); 9N (11); 10B (2); 10F/10C/33C (3); 13 (4); 15A/15F (4); 16F (9); 17F (1); 20 (2); 21 (2); 23A (5); 23B (5); 24F (11); 28A (1); 29 (2); 31 (5); 34 (4); 35A/35/42 (1); 35B (2); 35F/47 (3); 38/25 (3); 42 (1)
				R (N = 3)	1	2				
**Unspecified period**										
[90]	Kingdom of Bahrain	17	Penicillin	S (N = 5)	0	2				6 (2), 19 (1)
				I (N = 7)	1	0				6 (5), 19 (1)
				R (N = 4)	0	0				6 (1), 19 (2), 23 (1)
			Erythromycin	S (N = 8)	0	2				6 (4), 19 (2)
				R (N = 8)	1	0				6 (4), 19 (2), 23 (1)
			Tetracycline	S (N = 10)	0	2				6 (6), 19 (2)
				R (N = 7)	1	0				6 (3), 19 (2), 23 (1)

Background colors were used to indicate the pneumococcal vaccine (PCV) coverage period and to distinguish the respective studies.

## Data Availability

No new data were created or analyzed in this study. Data sharing is not applicable to this article.

## Supplementary Materials

The following supporting information can be downloaded at: https://www.mdpi.com/article/10.3390/vaccines13090923/s1, Supplement S1: Search strategy: from inception to 24 January 2024; Figure S1: Overall Serotype distribution in the MENA region (N = 5902 IPD cases) (55 original studies up to 24 January 2024); Table S1: Reporting Quality of Included Observational Studies Based on the STROBE Guidelines; Table S2: Characteristics of the 89 studies satisfying the inclusion criteria of the systematic review (Original studies up to 24 January 2024); Table S3: Serotype Distribution according to Pneumococcal Conjugate Vaccine (PCV) coverage by country in the MENA region (55 original studies up to 24 January 2024); Table S4: Serotype Distribution by country in the MENA region (59 original studies up to 24 January 2024); Table S5: Serotype Distribution by country and age groups in the MENA region (17 original studies up to 24 January 2024); Table S6: Antimicrobial susceptibility among IPD cases in the MENA region by country (36 original studies up to 24 January 2024); Table S7: Antimicrobial susceptibility among IPD cases in the MENA region by country and detected serotypes (13 original studies up to 24 January 2024).

## Abbreviations

The following abbreviations are used in this manuscript:

PCV	Pneumococcal conjugate vaccine
IPD	Invasive pneumococcal disease
MENA	Middle East and North Africa
AMR	Antimicrobial resistance
VT	Vaccine type
NVT	Non-vaccine type
WHO	World Health Organization
PPV23	23 valent pneumococcal polysaccharide vaccine
USA	United States of America
EPI	Extended Program of Immunization
STROBE	Strengthening the Reporting of Observational Studies in Epidemiology
NIP	National immunization program
NT	Non-typeable isolates
