# Supplementary material for: Changing Landscape of Invasive Pneumococcal Disease Serotypes and Antimicrobial Resistance Following Pneumococcal Conjugate Vaccine Introduction in the Middle East and North Africa Region: A Systematic Review
Source: Vaccines (Basel). 2025 Aug 29;13(9):923. doi: 10.3390/vaccines13090923 (PMC12474362; doi:10.3390/vaccines13090923)
Supplement: Supplementary file 1 [file vaccines-13-00923-s001.zip › vaccines-3759464-supplementary.pdf]

Supplementary material

Supplement S1. Search strategy from inception to January 24, 2024.

Medline:

Click to run search

The above Jumpstart will only work for users who have access to this specific database.

Database:

Ovid MEDLINE(R) and Epub Ahead of Print, In-Process, In-Data-Review & Other Non-Indexed Citations and Daily <1946 to January 24, 2024>

#	Query	Results from 24 Jan 2024
1	Streptococcus pneumoniae/ or exp Pneumococcal Infections/ or Streptococcaceae/ or Streptococcus/	58,888
2	(pneumoc* or streptoc* or diplococ*).mp.	192,295
3	1 or 2	192,295
4	middle east/ or afghanistan/ or bahrain/ or iran/ or iraq/ or jordan/ or kuwait/ or lebanon/ or oman/ or qatar/ or saudi arabia/ or syria/ or turkey/ or united arab emirates/ or yemen/ or pakistan/	159,050
5	exp Africa, Northern/	42,147
6	comoros/ or djibouti/ or sudan/ or mauritania/ or somalia/ or Arabs/ or Arab world/	14,390
7	(algeri* or Algiers or bahrain* or egypt* or cairo or moroc* or maroc* or iraq* or jordan* or Am?an or kuw?it* or lebanon or lebanese or liban* or Beirut or Beyro?t? or y?m?n* or aden or sanaa or UAE or Emirat* or (abu adj dhabi) or dubai or libya* or Tripoli or oman* or muscat or palestin* or g?az?a or (west* adj2 bank) or qatar* or katar* or quatar* or saudi* or KSA or Syria* or Syrie* or Damascus or tunis* or ((east* or north*) adj2 africa*) or sudan* or afghani* or Tal?ban or Bag?dad* or Iraq* or irak* or iran* or T?hran* or djibouti* or somali* or mauritania* or ifni or (trucial adj state*) or MENA or EMRO or ((middle or near) adj2 east*) or (east* adj2 mediterranean) or orient or arabs or arab* or arabia or levant or damas or pakistani* or comoros* or istanbul or ankara).mp.	491,298
8	4 or 5 or 6 or 7	544,698
9	3 and 8	2,938

Streptococcus pneumoniae/ or exp Pneumococcal Infections/ or Streptococcaceae/ or Streptococcus/

(pneumoc* or streptoc* or diplococ*).mp.

1 or 2

middle east/ or afghanistan/ or bahrain/ or iran/ or iraq/ or jordan/ or kuwait/ or lebanon/ or oman/ or qatar/ or saudi arabia/ or syria/ or turkey/ or united arab emirates/ or yemen/ or pakistan/

exp Africa, Northern/

comoros/ or djibouti/ or sudan/ or mauritania/ or somalia/ or Arabs/ or Arab world/

(algeri* or Algiers or bahrain* or egypt* or cairo or moroc* or maroc* or iraq* or jordan* or Am?an or kuw?it* or lebanon or lebanese or liban* or Beirut or Beyro?t? or y?m?n* or aden or sanaa or UAE or Emirat* or (abu adj dhabi) or dubai or libya* or Tripoli or oman* or muscat or palestin* or g?az?a or (west* adj2 bank) or qatar* or katar* or quatar* or saudi* or KSA or Syria* or Syrie* or Damascus or tunis* or ((east* or north*) adj2 africa*) or sudan* or afghani* or Tal?ban or Bag?dad* or Iraq* or irak* or iran* or T?hran* or djibouti* or somali* or mauritania* or ifni or (trucial adj state*) or MENA or EMRO or ((middle or near) adj2 east*) or (east* adj2 mediterranean) or orient or arabs or arab* or arabia or levant or damas or pakistani* or comoros* or istanbul or ankara).mp.

4 or 5 or 6 or 7

3 and 8

https://ovidsp.ovid.com/ovidweb.cgi?T=JS&NEWS=N&PAGE=main&SHAREDSEARCHID=60k24XifThbcl8krXHYVGppM32XrU2f5dd7FtT2gAkvwupDOqKDURIFWfi91yvZRu

CINAHL:

Search ID#	Search Terms	Search Options	Actions	
☑	S7	S3 AND S6	**Expanders** - Apply equivalent subjects<br>**Search modes** - Boolean/Phrase	**View Results** (572)<br>**View Details**<br>**Edit**
☑	S6	S4 OR S5	**Expanders** - Apply equivalent subjects<br>**Search modes** - Boolean/Phrase	**View Results** (141,579)<br>**View Details**<br>**Edit**
☑	S5	TI (algeri* OR Algier* OR bahrain* OR egypt* OR cairo OR moroc* OR maroc* OR iraq* OR jordan* OR Am#an OR kuw#it* OR lebanon OR lebanese OR liban* OR Beirut OR Beyro#t# OR y#m#n* OR aden OR sanaa OR UAE OR Emirat* OR abu	**Expanders** - Apply equivalent subjects<br>**Search modes** - Boolean/Phrase	**View Results** (114,248)<br>**View Details**<br>**Edit**

		dhabi OR dubai OR libya* OR Tripoli OR oman* OR muscat OR palestin* OR g#az#a OR (west* N2 bank) OR qatar* OR katar* OR quatar* OR saudi* OR KSA OR Syria* OR Syrie* OR Damascus OR tunis* OR ((east* OR north*) N2 africa*) OR sudan* OR afghani* OR Tal#ban OR Bag#dad* OR Iraq* OR irak* OR iran* OR T#hran* OR djibouti* OR somali* OR mauritania* OR ifni OR (trucial W1 state*) OR MENA OR EMRO OR ((middle OR near) N2 east*) OR (east* N2 mediterranean) OR orient OR arabs OR arab* OR arabia OR levant OR damas OR pakistani* OR comoros* OR istanbul OR ankara) OR AB (algeri* OR Algiers OR bahrain* OR egypt* OR cairo OR moroc* OR maroc* OR iraq* OR jordan* OR Am#an OR kuw#it* OR lebanon OR lebanese OR liban* OR Beirut OR Beyro#t# OR y#m#n* OR aden OR sanaa		

		OR UAE OR Emirat* OR (abu W1 dhabi) OR dubai OR libya* OR Tripoli OR oman* OR muscat OR palestin* OR g#az#a OR (west* N2 bank) OR qatar* OR katar* OR quatar* OR saudi* OR KSA OR Syria* OR Syrie* OR Damascus OR tunis* OR ((east* OR north*) N2 africa*) OR sudan* OR afghani* OR Tal#ban OR Bag#dad* OR Iraq* OR irak* OR iran* OR T#hran* OR djibouti* OR somali* OR mauritania* OR ifni OR (trucial W1 state*) OR MENA OR EMRO OR ((middle OR near) N2 east*) OR (east* N2 mediterranean) OR orient OR arabs OR arab* OR arabia OR levant OR damas OR pakistani* OR comoros* OR istanbul OR ankara) OR MW (algeri* OR Algiers OR bahrain* OR egypt* OR cairo OR moroc* OR maroc* OR iraq* OR jordan* OR Am#an OR kuw#it* OR lebanon OR lebanese OR liban* OR Beirut OR Beyro#t# OR		

		y#m#n* OR aden OR sanaa OR UAE OR Emirat* OR (abu W1 dhabi) OR dubai OR libya* OR Tripoli OR oman* OR muscat OR palestin* OR g#az#a OR (west* N2 bank) OR qatar* OR katar* OR quatar* OR saudi* OR KSA OR Syria* OR Syrie* OR Damascus OR tunis* OR ((east* OR north*) N2 africa*) OR sudan* OR afghani* OR Tal#ban OR Bag#dad* OR Iraq* OR irak* OR iran* OR T#hran* OR djibouti* OR somali* OR mauritania* OR ifni OR (trucial W1 state*) OR MENA OR EMRO OR ((middle OR near) N2 east*) OR (east* N2 mediterranean) OR orient OR arabs OR arab* OR arabia OR levant OR damas OR pakistani* OR comoros* OR istanbul OR ankara) Show Less		
☑	S4	(MH "Middle East") OR (MH "Afghanistan") OR (MH "Bahrain") OR (MH "Iran") OR (MH "Iraq") OR (MH "Jordan") OR (MH	**Expanders -** Apply equivalent subjects<br>**Search modes -** Boolean/Phrase	**View Results** (105,834)<br>**View Details**<br>**Edit**

		"Kuwait") OR (MH "Lebanon") OR (MH "Oman") OR (MH "Qatar") OR (MH "Saudi Arabia") OR (MH "Syria") OR (MH "Turkey") OR (MH "United Arab Emirates") OR (MH "Yemen") OR (MH "Pakistan") OR (MH "Africa, Northern+") OR (MH "Djibouti") OR (MH "Somalia") OR (MH "Sudan") OR (MH "Mauritania") OR (MH "Arabs")		
☑	S3	S1 OR S2	**Expanders -** Apply equivalent subjects<br>**Search modes -** Boolean/Phrase	View Results (25,033)<br>View Details<br>Edit
☑	S2	TI (pneumoc* OR streptoc* OR diplococ*) OR AB (pneumoc* OR streptoc* OR diplococ*) OR MW (pneumoc* OR streptoc* OR diplococ*)	**Expanders -** Apply equivalent subjects<br>**Search modes -** Boolean/Phrase	View Results (25,033)<br>View Details<br>Edit
☑	S1	(MH "Pneumococcal Infections+") OR (MH "Streptococcus")	**Expanders -** Apply equivalent subjects<br>**Search modes -** Boolean/Phrase	View Results (7,803)<br>View Details<br>Edit

Cochrane:

Date Run: 24/01/2024 08:39:33

ID	Search	Hits
#1	MeSH descriptor: [Streptococcus pneumoniae] this term only	554
#2	MeSH descriptor: [Pneumococcal Infections] explode all trees	185
#3	MeSH descriptor: [Streptococcus] this term only	287
#4	MeSH descriptor: [Streptococcaceae] this term only	5

#5	(pneumoc* OR streptoc* OR diplococ*):ti,ab,kw	7856
#6	#1 OR #2 OR #3 OR #4 OR #5	7856
#7	MeSH descriptor: [Middle East] this term only	41
#8	MeSH descriptor: [Afghanistan] this term only	37
#9	MeSH descriptor: [Bahrain] this term only	8
#10	MeSH descriptor: [Iran] this term only	1185
#11	MeSH descriptor: [Iraq] this term only	47
#12	MeSH descriptor: [Jordan] this term only	70
#13	MeSH descriptor: [Kuwait] this term only	33
#14	MeSH descriptor: [Lebanon] this term only	56
#15	MeSH descriptor: [Oman] this term only	17
#16	MeSH descriptor: [Qatar] this term only	18
#17	MeSH descriptor: [Saudi Arabia] this term only	160
#18	MeSH descriptor: [Syria] this term only	25
#19	MeSH descriptor: [Turkey] this term only	770
#20	MeSH descriptor: [United Arab Emirates] this term only	22
#21	MeSH descriptor: [Yemen] this term only	5
#22	MeSH descriptor: [Pakistan] this term only	428
#23	MeSH descriptor: [Africa, Northern] explode all trees	473
#24	MeSH descriptor: [Comoros] this term only	1
#25	MeSH descriptor: [Djibouti] this term only	2
#26	MeSH descriptor: [Sudan] this term only	82
#27	MeSH descriptor: [Mauritania] this term only	4
#28	MeSH descriptor: [Somalia] this term only	18
#29	MeSH descriptor: [Arabs] this term only	45
#30	MeSH descriptor: [Arab World] this term only	1

#31 (algeri* OR algiers OR bahrain* OR egypt* OR cairo OR moroc* OR maroc* OR jordan* OR am?an OR kuw?it* OR lebanon OR lebanese OR liban* OR beirut OR beyro?t? OR y?m?n* OR aden OR sanaa OR uae OR emirat* OR dubai OR libya* OR tripoli OR oman* OR muscat OR palestin* OR g?az?a OR qatar* OR katar* OR quatar* OR saudi* OR ksa OR syria* OR syrie* OR damascus OR tunis* OR sudan* OR afghani* OR tal?ban OR bag?dad* OR iraq* OR irak* OR iran* OR t?hran* OR djibouti* OR somali* OR mauritania* OR ifni OR mena OR emro OR orient OR arabs OR arab? OR

arabia OR levant OR damas OR pakistani* OR comoros* OR istanbul OR Ankara):ti,ab,kw 25934

#32 (east* NEAR/2 mediterranean):ti,ab,kw 31

#33 ((east* OR north*) NEAR/2 africa*):ti,ab,kw 343

#34 (abu NEXT/1 dhabi):ti,ab,kw 16

#35 (west* NEAR/2 bank):ti,ab,kw 16

#36 (middle NEAR/2 east*):ti,ab,kw 464

#37 (near NEAR/2 east*):ti,ab,kw 0

#38 (trucial NEXT/1 state*):ti,ab,kw 0

#39 #7 OR #8 OR #9 OR #10 OR #11 OR #12 OR #13 OR #14 OR #15 OR #16 OR #17 OR #18 OR #19 OR #20 OR #21 OR #22 OR #23 OR #24 OR #25 OR #26 OR #27 OR #28 OR #29 OR #30 OR #31 OR #32 OR #33 OR #34 OR #35 OR #36 OR #37 OR #38 1628625

#40 #6 AND #39 156

PubMed:

Query	Items found	Time
Search ((streptococcus pneumoniae [MeSH:noexp] OR Pneumococcal Infections [MeSH] OR Streptococcaceae [MeSH:noexp] OR Streptococcus [MeSH:noexp] OR pneumoc*[tw] OR streptoc*[tw] OR diplococ*[tw])) AND (middle east [MeSH:noexp] OR afghanistan [MeSH:noexp] OR bahrain [MeSH:noexp] OR iran [MeSH:noexp] OR iraq [MeSH:noexp] OR jordan [MeSH:noexp] OR kuwait [MeSH:noexp] OR lebanon [MeSH:noexp] OR oman [MeSH:noexp] OR qatar [MeSH:noexp] OR saudi arabia [MeSH:noexp] OR syria [MeSH:noexp] OR turkey [MeSH:noexp] OR united arab emirates [MeSH:noexp] OR yemen [MeSH:noexp] OR pakistan [MeSH:noexp] OR Africa, Northern [MeSH] OR comoros [MeSH:noexp] OR djibouti [MeSH:noexp] OR sudan [MeSH:noexp] OR mauritania [MeSH:noexp] OR somalia [MeSH:noexp] OR Arabs [MeSH:noexp] OR Arab world [MeSH:noexp] OR algeri*[tw] OR Algier*[tw] OR bahrain*[tw] OR egypt*[tw] OR cairo[tw] OR moroc*[tw] OR maroc*[tw] OR iraq*[tw] OR jordan*[tw] OR Amman[tw] OR Aman[tw] OR kuwait*[tw] OR lebanon[tw] OR lebanese[tw] OR liban*[tw] OR Beirut[tw] OR Beyrout[tw] OR Beyrouth [tw] OR yemen*[tw] OR yamen*[tw] OR aden[tw] OR sanaa[tw] OR UAE[tw] OR Emirat*[tw] OR abu-dhabi[tw] OR dubai[tw] OR libya*[tw] OR Tripoli[tw] OR oman*[tw] OR muscat[tw] OR palestin*[tw] OR gaza[tw] OR ghaza[tw] OR west bank[tw] OR western bank[tw] OR qatar*[tw] OR quatar*[tw] OR saudi*[tw] OR KSA[tw] OR Syria*[tw] OR Syrie*[tw] OR Damascus[tw] OR tunis*[tw] OR east-africa*[tw] OR eastern-africa*[tw] OR north-africa*[tw] OR northern-africa*[tw] OR sudan*[tw] OR afghani*[tw] OR Taliban[tw] OR Bagdad*[tw] OR Baghdad*[tw] OR Iraq*[tw] OR iran*[tw] OR Tehran*[tw] OR tahran*[tw] OR djibouti*[tw] OR somali*[tw] OR mauritania*[tw] OR ifni[tw] OR trucial state*[tw] OR MENA[tw] OR EMRO[tw] OR middle east*[tw] OR near east*[tw] OR east-mediterranean[tw] OR eastern-	2,843	04:47:33

mediterranean[tw] OR orient[tw] OR arabs[tw] OR arab*[tw] OR levant[tw] OR damas[tw] OR pakistani*[tw] OR comoros*[tw] OR istanbul[tw] OR ankara[tw])		
Search middle east [MeSH:noexp] OR afghanistan [MeSH:noexp] OR bahrain [MeSH:noexp] OR iran [MeSH:noexp] OR iraq [MeSH:noexp] OR jordan [MeSH:noexp] OR kuwait [MeSH:noexp] OR lebanon [MeSH:noexp] OR oman [MeSH:noexp] OR qatar [MeSH:noexp] OR saudi arabia [MeSH:noexp] OR syria [MeSH:noexp] OR turkey [MeSH:noexp] OR united arab emirates [MeSH:noexp] OR yemen [MeSH:noexp] OR pakistan [MeSH:noexp] OR Africa, Northern [MeSH] OR comoros [MeSH:noexp] OR djibouti [MeSH:noexp] OR sudan [MeSH:noexp] OR mauritania [MeSH:noexp] OR somalia [MeSH:noexp] OR Arabs [MeSH:noexp] OR Arab world [MeSH:noexp] OR algeri*[tw] OR Algier*[tw] OR bahrain*[tw] OR egypt*[tw] OR cairo[tw] OR moroc*[tw] OR maroc*[tw] OR iraq*[tw] OR jordan*[tw] OR Amman[tw] OR Aman[tw] OR kuwait*[tw] OR lebanon[tw] OR lebanese[tw] OR liban*[tw] OR Beirut[tw] OR Beyrout[tw] OR Beyrouth [tw] OR yemen*[tw] OR yamen*[tw] OR aden[tw] OR sanaa[tw] OR UAE[tw] OR Emirat*[tw] OR abu-dhabi[tw] OR dubai[tw] OR libya*[tw] OR Tripoli[tw] OR oman*[tw] OR muscat[tw] OR palestin*[tw] OR gaza[tw] OR ghaza[tw] OR west bank[tw] OR western bank[tw] OR qatar*[tw] OR quatar*[tw] OR saudi*[tw] OR KSA[tw] OR Syria*[tw] OR Syrie*[tw] OR Damascus[tw] OR tunis*[tw] OR east-africa*[tw] OR eastern-africa*[tw] OR north-africa*[tw] OR northern-africa*[tw] OR sudan*[tw] OR afghani*[tw] OR Taliban[tw] OR Bagdad*[tw] OR Baghdad*[tw] OR Iraq*[tw] OR iran*[tw] OR Tehran*[tw] OR tahran*[tw] OR djibouti*[tw] OR somali*[tw] OR mauritania*[tw] OR ifni[tw] OR trucial state*[tw] OR MENA[tw] OR EMRO[tw] OR middle east*[tw] OR near east*[tw] OR east-mediterranean[tw] OR eastern-mediterranean[tw] OR orient[tw] OR arabs[tw] OR arab*[tw] OR levant[tw] OR damas[tw] OR pakistani*[tw] OR comoros*[tw] OR istanbul[tw] OR ankara[tw]	531,848	04:44:45
Search streptococcus pneumoniae [MeSH:noexp] OR Pneumococcal Infections [MeSH] OR Streptococcaceae [MeSH:noexp] OR Streptococcus [MeSH:noexp] OR pneumoc*[tw] OR streptoc*[tw] OR diplococ*[tw]	192,277	04:44:11

Global Health:

All	Search term	Date (GMT)	Results	Actions
	((algeri* OR Algier* OR bahrain OR egypt* OR cairo OR moroc* OR maroc* OR iraq* OR jordan* OR Amman OR kuwait* OR lebanon OR lebanese OR liban* OR Beirut OR Beyrouth OR yemen* OR aden OR sanaa OR UAE OR Emirat* OR abu-dhabi OR dubai OR libya* OR Tripoli OR oman* OR muscat OR palestin* OR g?aza OR west-bank OR western-bank OR q?atar* OR saudi* OR KSA OR Syria* OR Syrie* OR Damascus OR tunis* OR east-africa* OR eastern-africa* OR north-africa* OR northern-africa* OR sudan* OR afghani* OR Tal?ban OR Bag?dad* OR Iraq* OR iran* OR Tehran* OR djibouti* OR somali* OR mauritania* OR ifni OR trucial-state* OR MENA OR EMRO OR middle east* OR near east* OR east mediterranean OR eastern mediterranean OR orient OR arabs OR arab* OR levant OR damas OR pakistani* OR comoros* OR istanbul OR ankara) OR ("middle east" OR "Afghanistan" OR "Bahrain" OR "iran" OR "Iraq" OR "Jordan" OR "Kuwait" OR "Lebanon" OR "oman" OR "Qatar" OR "saudi arabia" OR "syria" OR "turkey" OR "united arab emirates" OR "yemen" OR "Pakistan" OR " Africa, Northern" OR "comoros" OR "Djibouti" OR "sudan" OR "mauritania" OR "somalia" OR "Arabs" OR "Arab world")) AND ((title:(pneumoc* OR streptoc* OR diplococ*) OR ab:(pneumoc* OR streptoc* OR diplococ*) OR subject:(pneumoc* OR streptoc* OR diplococ*)) OR ("Streptococcus" OR "Streptococcus pneumoniae" OR "Streptococcaceae"))<br>Refinements:	24 Jan 2024 16:51	5,732	
	(algeri* OR Algier* OR bahrain OR egypt* OR cairo OR moroc* OR maroc* OR iraq* OR jordan* OR Amman OR kuwait* OR lebanon OR lebanese OR liban* OR Beirut OR Beyrouth OR yemen* OR aden OR sanaa OR UAE OR Emirat* OR abu-dhabi OR dubai OR libya* OR Tripoli OR oman* OR muscat OR palestin* OR g?aza OR west-bank OR western-bank OR q?atar* OR saudi* OR KSA OR Syria* OR Syrie* OR Damascus OR tunis* OR east-africa* OR eastern-africa* OR north-africa* OR northern-africa* OR sudan* OR afghani* OR Tal?ban OR Bag?dad* OR Iraq* OR iran* OR Tehran* OR djibouti* OR somali* OR mauritania* OR ifni OR trucial-state* OR MENA OR EMRO OR middle east* OR near east* OR east mediterranean OR eastern mediterranean OR orient OR arabs OR arab* OR levant OR damas OR pakistani* OR comoros* OR istanbul OR ankara) OR ("middle east" OR "Afghanistan" OR "Bahrain" OR "iran" OR "Iraq" OR "Jordan" OR "Kuwait" OR "Lebanon" OR "oman" OR "Qatar" OR "saudi arabia" OR "syria" OR "turkey" OR "united arab emirates" OR "yemen" OR "Pakistan" OR " Africa, Northern" OR "comoros" OR "Djibouti" OR "sudan" OR "mauritania" OR "somalia" OR "Arabs" OR "Arab world")<br>Refinements:	24 Jan 2024 16:50	374,195	
	algeri* OR Algier* OR bahrain OR egypt* OR cairo OR moroc* OR maroc* OR iraq* OR jordan* OR Amman OR kuwait* OR lebanon OR lebanese OR liban* OR Beirut OR Beyrouth OR yemen* OR aden OR sanaa OR UAE OR Emirat* OR abu-dhabi OR dubai OR libya* OR Tripoli OR oman* OR muscat OR palestin* OR g?aza OR west-bank OR western-bank OR q?atar* OR saudi* OR KSA OR Syria* OR Syrie* OR Damascus OR tunis* OR east-africa* OR eastern-africa* OR north-africa* OR northern-africa* OR sudan* OR afghani* OR Tal?ban OR Bag?dad* OR Iraq* OR iran* OR Tehran* OR djibouti* OR somali* OR mauritania* OR ifni OR trucial-state* OR MENA OR EMRO OR middle east* OR near east* OR east mediterranean OR eastern mediterranean OR orient OR arabs OR arab* OR levant OR damas OR pakistani* OR comoros* OR istanbul OR ankara<br>Refinements:	24 Jan 2024 16:45	49,432	

☐	"middle east" OR "Afghanistan" OR "Bahrain" OR "iran" OR "Iraq" OR "Jordan" OR "Kuwait" OR "Lebanon" OR "oman" OR "Qatar" OR "saudi arabia" OR "syria" OR "turkey" OR "united arab emirates" OR "yemen" OR "Pakistan" OR " Africa, Northern" OR "comoros" OR "Djibouti" OR "sudan" OR "mauritania" OR "somalia" OR "Arabs" OR "Arab world"<br>Refinements:	24 Jan 2024 16:43	950,973	
☐	(title:(pneumoc* OR streptoc* OR diplococ*) OR ab:(pneumoc* OR streptoc* OR diplococ*) OR subject:(pneumoc* OR streptoc* OR diplococ*)) OR ("Streptococcus" OR "Streptococcus pneumoniae" OR "Streptococcaceae")<br>Refinements:	24 Jan 2024 16:43	112,134	
☐	title:(pneumoc* OR streptoc* OR diplococ*) OR ab:(pneumoc* OR streptoc* OR diplococ*) OR subject:(pneumoc* OR streptoc* OR diplococ*)<br>Refinements:	24 Jan 2024 16:43	96,993	
☐	"Streptococcus" OR "Streptococcus pneumoniae" OR "Streptococcaceae"<br>Refinements:	24 Jan 2024 16:43	95,902	

Global Index Medicus:

tw:("Pneumococcal Infection" OR "Pneumococcal Infections" OR "Diplococcus" OR "Streptococcus" OR "Pneumococcus" OR "Streptococcus Pneumoniae" OR "Streptococcaceae") AND (db:("IMEMR"))

EMBASE:

No.	Query	Results	Date

#7,"#3 AND #6",3781,24 Jan 2024

#6,"#4 OR #5",493331,24 Jan 2024

#5,"algeri*:ti,ab,kw OR algiers:ti,ab,kw OR bahrain*:ti,ab,kw OR egypt*:ti,ab,kw OR cairo:ti,ab,kw OR moroc*:ti,ab,kw OR maroc*:ti,ab,kw OR jordan*:ti,ab,kw OR am$an:ti,ab,kw OR kuw$it*:ti,ab,kw OR lebanon:ti,ab,kw OR lebanese:ti,ab,kw OR liban*:ti,ab,kw OR beirut:ti,ab,kw OR beyro$t$:ti,ab,kw OR ymn*:ti,ab,kw OR aden:ti,ab,kw OR sanaa:ti,ab,kw OR uae:ti,ab,kw OR emirat*:ti,ab,kw OR ((abu NEXT/1 dhabi):ti,ab,kw) OR dubai:ti,ab,kw OR libya*:ti,ab,kw OR tripoli:ti,ab,kw OR oman*:ti,ab,kw OR muscat:ti,ab,kw OR palestin*:ti,ab,kw OR gaza:ti,ab,kw OR ((west* NEAR/2 bank):ti,ab,kw) OR qatar*:ti,ab,kw OR katar*:ti,ab,kw OR quatar*:ti,ab,kw OR saudi*:ti,ab,kw OR ksa:ti,ab,kw OR syria*:ti,ab,kw OR syrie*:ti,ab,kw OR damascus:ti,ab,kw OR tunis*:ti,ab,kw OR (((east* OR north*) NEAR/2 africa*):ti,ab,kw) OR sudan*:ti,ab,kw OR afghani*:ti,ab,kw OR tal$ban:ti,ab,kw OR bag$dad*:ti,ab,kw OR iraq*:ti,ab,kw OR irak*:ti,ab,kw OR iran*:ti,ab,kw OR t$hran*:ti,ab,kw OR djibouti*:ti,ab,kw OR somali*:ti,ab,kw OR mauritania*:ti,ab,kw OR ifni:ti,ab,kw OR ((trucial NEXT/1 state*):ti,ab,kw) OR mena:ti,ab,kw OR emro:ti,ab,kw OR (((middle OR near) NEAR/2 east*):ti,ab,kw) OR ((east* NEAR/2 mediterranean):ti,ab,kw) OR orient:ti,ab,kw OR arabs:ti,ab,kw OR arab$:ti,ab,kw OR arabia:ti,ab,kw OR levant:ti,ab,kw OR damas:ti,ab,kw OR pakistani*:ti,ab,kw OR comoros*:ti,ab,kw OR istanbul:ti,ab,kw OR ankara:ti,ab,kw",430615,24 Jan 2024

#4,"'middle east'/de OR 'bahrain'/de OR 'iran'/de OR 'iraq'/exp OR 'jordan'/de OR 'kuwait'/de OR 'lebanon'/de OR 'oman'/de OR 'palestine'/de OR 'qatar'/de OR 'saudi arabia'/de OR 'syrian arab republic'/de OR 'turkey (republic)'/de OR 'united arab emirates'/de OR 'yemen'/de OR 'afghanistan'/de OR 'north africa'/exp OR 'arab world'/de OR 'comoros'/de OR 'djibouti'/de OR 'somalia'/de OR 'sudan'/de OR 'arab'/de",273893,24 Jan 2024

#3,"#1 OR #2",231147,24 Jan 2024

#2,"pneumoc*:ti,ab,kw OR streptoc*:ti,ab,kw OR diplococ*:ti,ab,kw",196458,24 Jan 2024

#1,"'streptococcaceae'/de OR 'streptococcus'/de OR 'streptococcus pneumoniae'/exp OR 'pneumococcal infection'/de OR 'pneumococcal meningitis'/exp",100155,24 Jan 2024

Scopus:

ID	Query	Documents	Date last run
result#7	((INDEXTERMS ("Streptococcus pneumoniae" OR "Pneumococcal Infections" OR streptococcaceae OR streptococcus)) OR (TITLE-ABS-KEY (pneumoc* OR streptoc* OR diplococ*))) AND ((INDEXTERMS ("middle east" OR afghanistan OR bahrain OR iran OR iraq OR jordan OR kuw	5,055	24 Jan 2024

ID	Query	Documents	Date last run
	ait OR lebanon OR oman OR qatar OR saudi AND arabia OR syria OR turkey OR "united arab emirates" OR yemen OR pakistan OR "Africa, Northern" OR comoros OR djibouti OR sudan OR mauritania OR somalia OR arabs OR "Arab world")) OR (TITLE-ABS-KEY (algeri* OR algiers OR bahrain* OR egypt* OR cairo OR moroc* OR maroc* OR iraq* OR jordan* OR am?an OR kuw#it* OR lebanon OR lebanese OR liban* OR beirut OR beyro#t? OR y#m#n* OR aden OR sanaa OR uae OR emirat* OR (abu-dhabi) OR dubai OR libya* OR tripoli OR oman* OR muscat OR palestin* OR g#az#a OR (west* W/2 bank) OR qatar* OR katar* OR quatar* OR saudi* OR ksa OR syria* OR syrie* OR damascus OR tunis* OR ((east* OR north*) W/2 africa*) OR sudan* OR afghani* OR tal#ban OR bag#dad* OR iraq* OR irak* OR iran* OR t#hran* OR djibouti* OR somali* OR mauritania* OR ifni OR (trucial-state*) OR mena OR emro OR ((middle OR near) W/2 east*) OR (east* W/2 mediterranean) OR orient OR arabs OR arab* OR arabia OR levant OR damas OR pakistani* OR comoros* OR istanbul OR ankara)))<br>View Less<br>Edit query		
result#6	(INDEXTERMS ("middle east" OR afghanistan OR bahrain OR iran OR iraq OR jordan OR kuwait OR lebanon OR oman OR qatar OR saudi AND arabia OR syria OR turkey OR "united arab emirates" OR yemen OR pakistan OR "Africa, Northern" OR comoros OR djibouti OR sudan OR mauritania OR somalia OR arabs OR "Arab world")) OR (TITLE-ABS-KEY (algeri* OR algiers OR bahrain* OR egypt* OR cairo OR moroc* OR maroc* OR iraq* OR jordan* OR am?an OR kuw#it* OR lebanon OR lebanese OR liban* OR beirut OR beyro#t? OR y#m#n* OR aden OR sanaa OR uae OR emirat* OR (abu-dhabi) OR dubai OR libya* OR tripoli OR oman* OR muscat OR palestin* OR g#az#a OR (west* W/2 bank) OR qatar* OR katar* OR quatar* OR saudi* OR ksa OR syria* OR syrie* OR damascus OR tunis* OR ((east* OR north*) W/2 africa*) OR sudan* OR afghani* OR tal#ban OR bag#dad* OR iraq* OR irak* OR iran* OR t#hran* OR djibouti* OR somali* OR mauritania* OR ifni OR (trucial-state*) OR mena OR emro OR ((middle OR near) W/2 east*) OR (east* W/2 mediterranean) OR orient OR arabs OR arab* OR arabia OR levant OR damas OR pakistani* OR comoros* OR istanbul OR ankara))	1,342,382	24 Jan 2024

ID	Query	Documents	Date last run
	View Less<br>Edit query		
result#5	TITLE-ABS-KEY (algeri* OR algiers OR bahrain* OR egypt* OR cairo OR moroc* OR maroc* OR iraq* OR jordan* OR am?an OR kuw#it* OR lebanon OR lebanese OR liban* OR beirut OR beyro#t? OR y#m#n* OR aden OR sanaa OR uae OR emirat* OR (abu-dhabi) OR dubai OR libya* OR tripoli OR oman* OR muscat OR palestin* OR g#az#a OR (west* W/2 bank) OR qatar* OR katar* OR quatar* OR saudi* OR ksa OR syria* OR syrie* OR damascus OR tunis* OR ((east* OR north*) W/2 africa*) OR sudan* OR afghani* OR tal#ban OR bag#dad* OR iraq* OR irak* OR iran* OR t#hran* OR djibouti* OR somali* OR mauritania* OR ifni OR (trucial-state*) OR mena OR emro OR ((middle OR near) W/2 east*) OR (east* W/2 mediterranean) OR orient OR arabs OR arab* OR arabia OR levant OR damas OR pakistani* OR comoros* OR istanbul OR ankara)<br>View Less<br>Edit query	1,342,357	24 Jan 2024
result#4	INDEXTERMS ("middle east" OR afghanistan OR bahrain OR iran OR iraq OR jordan OR kuwait OR lebanon OR oman OR qatar OR saudi AND arabia OR syria OR turkey OR "united arab emirates" OR yemen OR pakistan OR "Africa, Northern" OR comoros OR djibouti OR sudan OR mauritania OR somalia OR arabs OR "Arab world")<br>View Less<br>Edit query	49,190	24 Jan 2024
result#3	(INDEXTERMS ("Streptococcus pneumoniae" OR "Pneumococcal Infections" OR streptococcaceae OR streptococcus)) OR (TITLE-ABS-KEY (pneumoc* OR streptoc* OR diplococ*))	296,404	24 Jan 2024
result#2	TITLE-ABS-KEY (pneumoc* OR streptoc* OR diplococ*)	296,404	24 Jan 2024
result#1	INDEXTERMS ("Streptococcus pneumoniae" OR "Pneumococcal Infections" OR streptococcaceae OR streptococcus)	189,671	24 Jan 2024

EBSCO:

EBSCOhost

Wednesday, January 24, 2024 9:33:08 AM

#	Query	Limiters/Expanders	Last Run Via	Results
S7	S3 AND S6	Limiters - Publication Date: 20200101-20240131<br>Expanders - Apply equivalent subjects<br>Search modes - Boolean/Phrase	Interface - EBSCOhost Research Databases<br>Search Screen - Advanced Search<br>Database - CINAHL Ultimate	150
S6	S4 OR S5	Expanders - Apply equivalent subjects<br>Search modes - Boolean/Phrase	Interface - EBSCOhost Research Databases<br>Search Screen - Advanced Search<br>Database - CINAHL Ultimate	141,579
S5	TI (algeri* OR Algier* OR bahrain* OR egypt* OR cairo OR moroc* OR maroc* OR iraq* OR jordan* OR Am#an OR kuw#it* OR lebanon OR lebanese OR liban* OR Beirut OR Beyro#t# OR y#m#n* OR aden OR sanaa OR UAE OR Emirat* OR abu dhabi OR dubai OR libya* OR Tripoli OR oman* OR muscat OR palestin* OR g#az#a OR (west* N2 bank) OR qatar* OR katar* OR quatar* OR saudi* OR KSA OR Syria* OR Syrie* OR Damascus OR tunis* OR ((east* OR north*) N2 africa*) OR sudan* OR afghani* OR Tal#ban OR Bag#dad* OR Iraq* OR irak* OR iran* OR T#hran* OR djibouti*	Expanders - Apply equivalent subjects<br>Search modes - Boolean/Phrase	Interface - EBSCOhost Research Databases<br>Search Screen - Advanced Search<br>Database - CINAHL Ultimate	114,248

OR somali* OR
mauritania* OR ifni OR
(trucial W1 state*) OR
MENA OR EMRO OR
((middle OR near) N2
east*) OR (east* N2
mediterranean) OR orient
OR arabs OR arab* OR
arabia OR levant OR
damas OR pakistani* OR
comoros* OR istanbul OR
ankara) OR AB (algeri*
OR Algiers OR bahrain*
OR egypt* OR cairo OR
moroc* OR maroc* OR
iraq* OR jordan* OR
Am#an OR kuw#it* OR
lebanon OR lebanese OR
liban* OR Beirut OR
Beyro#t# OR y#m#n* OR
aden OR sanaa OR UAE
OR Emirat* OR (abu W1
dhabi) OR dubai OR libya*
OR Tripoli OR oman* OR
muscat OR palestin* OR
g#az#a OR (west* N2
bank) OR qatar* OR katar*
OR quatar* OR saudi* OR
KSA OR Syria* OR Syrie*
OR Damascus OR tunis*
OR ((east* OR north*) N2
africa*) OR sudan* OR
afghani* OR Tal#ban OR
Bag#dad* OR Iraq* OR
irak* OR iran* OR T#hran*
OR djibouti* OR somali*
OR mauritania* OR ifni
OR (trucial W1 state*) OR
MENA OR EMRO OR
((middle OR near) N2
east*) OR (east* N2
mediterranean) OR orient
OR arabs OR arab* OR
arabia OR levant OR

	damas OR pakistani* OR comoros* OR istanbul OR ankara) OR MW (algeri* OR Algiers OR bahrain* OR egypt* OR cairo OR moroc* OR maroc* OR iraq* OR jordan* OR Am#an OR kuw#it* OR lebanon OR lebanese OR liban* OR Beirut OR Beyro#t# OR y#m#n* OR aden OR sanaa OR UAE OR Emirat* OR (abu W1 dhabi) OR dubai OR libya* OR Tripoli OR oman* OR muscat OR palestin* OR g#az#a OR (west* N2 bank) OR qatar* OR katar* OR quatar* OR saudi* OR KSA OR Syria* OR Syrie* OR Damascus OR tunis* OR ((east* OR north*) N2 africa*) OR sudan* OR afghani* OR Tal#ban OR Bag#dad* OR Iraq* OR irak* OR iran* OR T#hran* OR djibouti* OR somali* OR mauritania* OR ifni OR (trucial W1 state*) OR MENA OR EMRO OR ((middle OR near) N2 east*) OR (east* N2 mediterranean) OR orient OR arabs OR arab* OR arabia OR levant OR damas OR pakistani* OR comoros* OR istanbul OR ankara)			
S4	(MH "Middle East") OR (MH "Afghanistan") OR (MH "Bahrain") OR (MH "Iran") OR (MH "Iraq") OR (MH "Jordan") OR (MH "Kuwait") OR (MH	Expanders - Apply equivalent subjects<br>Search modes - Boolean/Phrase	Interface - EBSCOhost Research Databases<br>Search Screen - Advanced Search<br>Database - CINAHL Ultimate	105,834

	"Lebanon") OR (MH "Oman") OR (MH "Qatar") OR (MH "Saudi Arabia") OR (MH "Syria") OR (MH "Turkey") OR (MH "United Arab Emirates") OR (MH "Yemen") OR (MH "Pakistan") OR (MH "Africa, Northern+") OR (MH "Djibouti") OR (MH "Somalia") OR (MH "Sudan") OR (MH "Mauritania") OR (MH "Arabs")			
S3	S1 OR S2	Expanders - Apply equivalent subjects<br>Search modes - Boolean/Phrase	Interface - EBSCOhost Research Databases<br>Search Screen - Advanced Search<br>Database - CINAHL Ultimate	25,033
S2	TI (pneumoc* OR streptoc* OR diplococ*) OR AB (pneumoc* OR streptoc* OR diplococ*) OR MW (pneumoc* OR streptoc* OR diplococ*)	Expanders - Apply equivalent subjects<br>Search modes - Boolean/Phrase	Interface - EBSCOhost Research Databases<br>Search Screen - Advanced Search<br>Database - CINAHL Ultimate	25,033
S1	(MH "Pneumococcal Infections+") OR (MH "Streptococcus")	Expanders - Apply equivalent subjects<br>Search modes - Boolean/Phrase	Interface - EBSCOhost Research Databases<br>Search Screen - Advanced Search<br>Database - CINAHL Ultimate	7,803

Table S1. Reporting Quality of Included Observational Studies Based on the STROBE Guidelines

Author, Year	Percentage of satisfied STROBE items	Quality appraisal Category reporting
**Pre-PCV13 introduction**		
Tali-Maamar H. et al., 2012	57.90%	Good
Hecini-Hannachi A. et al., 2013	70%	Good
Ramdani-Bouguessa, N., et al., 2015	66.70%	Good
Ziane H, et al., 2016	75%	Good
Al Musawi M., 2012	80%	Excellent
Guirguis N. et al., 1983	63%	Good
Ostroff S. M. et al., 1996	86%	Excellent
Wasfy M. et al., 2005	67%	Good
Saadi A. T. et al., 2017	70.80%	Good
Tavana A. M. & Ataee R. A., 2013	55%	Good
Talebi M., 2016	64.70%	Good
Houri H. et al., 2017	68%	Good
Abdoli S. et al., 2020	75%	Good
Ghahfarokhi S.H. et al., 2020	69%	Good
Azimian A. et al., 2020	75%	Good
Beheshti M. et al., 2020	68%	Good
Esteghamati A. et al., 2021	61%	Good
Tabatabaie SR. et al., 2021	61%	Good
Mosadegh M. et al., 2022	75%	Good
Tabatabaei SR. et al., 2017	80%	Excellent
Tabatabaei SR et al., 2022	78%	Excellent
Kohanteb J. et al., 2007	57%	Good
Alam A.N. et al., 2017	52%	Good
Azarsa M. et al., 2019	65%	Good
Abu-Helalah M. et al., 2023	90%	Excellent
Johny M. et al., 1998	45.45%	Medium
Ahmed k. et al., 2000	45%	Medium
Mokaddas E.M. et al., 2007	67.80%	Good
Mokaddas, E.M. et al., 2008	67.00%	Good
Mokaddas E. & Albert M.J., 2012	70%	Good
Uwaydag M. et al., 1996	35%	Medium
Araj G.F et al., 1999	57.38%	Good
Uwaydah M. et al., 2006	35%	Medium
Hanna-Wakim R. et al., 2012	82%	Excellent

**Moghnieh R. et al., 2019**	82%	Excellent
**Borg M.A et al., 2008**	83%	Excellent
**Benbachir M. et al., 2012**	73%	Good
**ElMdaghri N., 2012**	60%	Good
**ElMdaghri N., 2012**	60%	Good
**Al-Yaqoubi M.M. & Elhag K. M., 2011**	70%	Good
**Mastro T. D. et al., 1991**	61%	Good
**Sadia S. et al., 2014**	75%	Good
**Riaz A. et al., 2019**	90%	Excellent
**Zafar A. et al., 2021**	79%	Good
**Kattan R. et al., 2011**	66.60%	Good
**Al Khal A.L. et al, 2007**	45%	Medium
**Elshafie, S. & Taj-Aldeen S.J., 2016**	53%	Good
**Al-Swailem A. et al., 2004**	50%	Medium
**Memish Z.A. et al., 2004**	75%	Good
**Al-Mazrou A. et al., 2005**	71%	Good
**Al Tawfiq J.A., 2004**	65%	Good
**Al-Tawfiq J. A., 2006**	66%	Good
**Shibl A. M., 2008**	55%	Good
**Shibl, A. M., et al.,2012**	61%	Good
**Qadri S.M. & Kroschinsky R., 1991**	35%	Medium
**Chowdhury M.N.H., et al., 1995**	76.80%	Excellent
**Kambal, A. et al., 1997**	81.50%	Excellent
**Al-Aqeeli A. A. et al., 2002**	45%	Medium
**Fouda S. I. et al., 2004**	50%	Medium
**Marzouk M. et al., 2015**	60.00%	Good
**Smaoui H. et al., 2009**	70%	Good
**Charfi F. et al., 2012**	58.50%	Good
**Raddaoui A., et al., 2015**	85%	Excellent
**Ktari S. et al., 2017**	95%	Excellent
**Haddad-Boubaker S. et al., 2020**	91%	Excellent
**Ktari S. et al., 2023**	77%	Excellent
**Percin D. et al., 2010**	80%	Excellent
**Ceyhan M. et al., 2010**	55%	Good
**Ceyhan M. et al., 2011**	50%	Medium
**Altun B. et al., 2006**	81%	Excellent
**Altun H.U. et al., 2015**	52%	Good
**Gur D. et al., 2001**	70%	Good
**Post-PCV13 introduction**		

**Diawara I., 2017**	36%	Medium
**Ikken Y. et al., 2020**	75%	Good
**Chikhaoui A. et al., 2022**	82%	Excellent
**A-Jardani A. et al., 2019**	89%	Excellent
**Al-Sherikh Y. A., et al., 2014**	50%	Medium
**Ozdemir H. et al., 2017**	91%	Excellent
**Hascelik G. et al., 2023**	86%	Excellent
**Kİttana F. N. A., et al., 2019**	82%	Excellent
**Ceyhan M. et al., 2020**	82%	Excellent
**Pre- and Post-PCV13 introduction**		
**Mokaddas, E. & Albert M. J., 2016**	75%	Good
**Reslan L. et al., 2022**	84%	Excellent
**Diawara I. et al., 2015**	86%	Excellent
**Nzoyikorera N. et al., 2023**	86%	Excellent
**Al-Waili B.R. et al., 2013**	65%	Good
**Ceyhan M. et al., 2013**	75%	Good
**Ceyhan M. et al., 2016**	93%	Excellent
**Unspecified period**		
**Haifa Al-Muhtaresh A. & Bindayna K.M., 2020**	92%	Excellent

Table S2. Characteristics of the 89 studies satisfying the inclusion criteria of the systematic review (Original studies up to January 24, 2024)

Author, Year	Period	Country (Population number: most recent value in 2023 - World Bank classification)	Setting	Population/ Age distribution	Sample size (Invasive samples)	Source (N)
**Pre-PCV13 introduction**						
**Tali-Maamar H. et al., 2012**	115 months: January 2001 to July 2010	Algeria (46,164,219)	1 university hospital	All age groups: <2y, 2-5y, 6-10y, 11-15y, >15y	167	Blood (41), CSF (111), Puncture fluid (Pleural fluid, peritoneal fluid, ascitic fluid, and gastric fluid) (15)
**Hecini-Hannachi A. et al., 2013**	84 months: 2005-2011	Algeria	1 university hospital	All age groups/ <18y and ≥18y	100	Blood (22), CSF (75), pleural fluid (3)
**Ramdani-Bouguessa, N., et al., 2015**	90 months: Jan 2005-Jun 2012	Algeria	7 university hospitals and 3 non-university hospitals	Children: <5y and >5-16y	97	Blood (21), CSF (53), Pleural fluid (15), joint fluid (5), peritoneal fluid (3)
**Ziane H, et al., 2016**	60 months: 1st January 2010 - 31st December 2014	Algeria	81 university hospitals	Pediatrics/ <1y, 1-2y, 3-5y	80	Not specified
**Al Musawi M., 2012**	60 months: 1 January 1999 - 31 December 2003	Bahrain (1,577,059)	5 hospitals	Pediatrics/ 0-1y, 1-2y, 2-3y, 3-4y, 4-5y mean age: 1.25 ±1.21	207	Blood, CSF and other invasive body fluids
**Guirguis N. et al., 1983**	11 months: 1977 and 1978	Egypt (114,535,772)	2 hospitals	All age groups/ >1y, 1-4y, 5-9y, 10-14y, 15-34y, >35y	142	CSF
**Ostroff S. M. et al., 1996**	18 months: between October 1991 and April 1993	Egypt	2 largest government-operated primary facilities for children with pneumonia in the Cairo area	Children 2m-60m	52	Blood (52)
**Wasfy M. et al., 2005**	72 months: 1998-2003	Egypt	13 community-based hospitals	All age groups/ <2y, 2-5y, 6-17y, 18-49y, 50-64y, ≥65y	205	CSF

**Saadi A. T. et al., 2017**	24 months: January 2014 and January 2016	Iraq (45,074,049)	1 university hospital	Pediatric/ 0 to 15 years	18	CSF
**Tavana A. M. & Ataee R. A., 2013**	12 months: 2009-2010	Iran (90,608,707)	Selected laboratory hospitals in Iranian central provinces were collected	All age groups	133	blood (15), CSF (6), Wound (8), Rhinit (4), Sinus (3), Eye invasive samples (26), Throat invasive samples (32), Lung samples (39)
**Talebi M., 2016**	36 months: 2011–2013	Iran	Clinics and private laboratories	All age groups	15	Not specified
**Houri H. et al., 2017**	32 months: July 2013 to March 2016	Iran	Several university and non-university hospitals	Pediatrics/ children <5y mean age: 2.7 ± 0.5y	53	Blood (21), CSF (32)
**Abdoli S. et al., 2020**	60 months: 2014-2018	Iran	1 university hospital	Pediatric/ Children <5y	106	CSF
**Ghahfarokhi S.H. et al., 2020**	8 months: Feb to September 2015 9 months: July 2018 till March 2019	Iran	1 university hospital	All age groups/ 1month to 72y	38	Blood (26), CSF (6), pleural fluid (4), synovial fluid (1), ascites fluid (1)
**Azimian A. et al., 2020**	60 months: 2014-2018	Iran	1 university hospital	Pediatric/ children <5y	51	Blood
**Beheshti M. et al., 2020**	12 months: October 2016-September 2017	Iran	2 university hospitals	Children (89%) and adults (11%)	44	Blood
**Esteghamati A. et al., 2021**	25 months: June 2017-August 2019	Iran	Unspecified number of hospitals	10 days to 92 years	55	Blood (13), CSF (37), and pleural fluid (5)
**Tabatabaie SR. et al., 2021**	12 months: March 2012-March 2013	Iran	1 university hospital and other regional hospitals	Children: 1 month to 18 years	83	Blood and CSF
**Mosadegh M. et al., 2022**	36 months: Between 2017 and 2019	Iran	>1 university hospital	1 month to 88 years/ mean age: 21 years	53	Blood (31) and CSF (22)
**Tabatabaei SR. et al., 2017**	35 months: September 2012 to July 2015	Iran	Various hospitals	All age groups	34	Blood (13), CSF (15), BAL (6)

**Tabatabaei SR et al., 2022**	12 months: November 14, 2016-November 15, 2017	Iran	2 university hospitals and 2 general hospitals	6 months to 10 years	20	CSF
**Kohanteb J. et al., 2007**	Not specified	Iran	1 university hospital	All age groups/Mean 28.9y (range: 1y-70y)	28	10 from blood (10), CSF (15), pleural fluid (3)
**Alam A.N. et al., 2017**	17 months: Nov 2014 - Mar 2016	Iran	2 university hospitals	All age groups	4	BAL (2), Blood (1), CSF (1)
**Azarsa M. et al., 2019**	8 months: Feb–Sep 2015	Iran	Hospitals affiliated to Tehran University of Medical Sciences	All age groups	27	Blood (10), CSF (4), BAL (5), tracheal aspirate (5), pleural fluid (2), ascites fluid (1)
**Abu-Helalah M. et al., 2023**	15 months: October 1, 2021-December 31, 2022	Jordan (11,439,213)	3 governmental hospital & 5 university hospitals	Age under 5 years/ Mean age: 15 ± 16.1 months	23	Blood
**Johny M. et al., 1998**	25 months: 1992 to June 1994 and 16 months: April 1995 to August 1996	Kuwait (4,853,420)	1 university hospital in Kuwait	All age groups	25	Blood (24) and CSF (1)
**Ahmed k. et al., 2000**	12 months: 5 November 1995 - 4 November 1996	Kuwait	3 university hospitals	Not specified	50	Blood (25), CSF (7), peritoneal fluid (1), BAL (2), appendicular abscess (1), endotracheal tube (13), peritoneal dialysis catheter (1)
**Mokaddas E.M. et al., 2007**	48 months: between January 2001, and December, 2004	Kuwait	11 university hospitals and 30 primary care centers in Kuwait	All age groups/ 0y-5y, 6y-15y, 16y-25y, 26y-35y, 36y-45y, 46y-55y, 56y-65y, ≥66y	122	Blood (68), CSF (3), bronchial aspirates (3), Pleural fluid (2), tracheal secretions (46)
**Mokaddas, E.M. et al., 2008**	24 months: between January 2004 and December 2005	Kuwait	11 secondary and tertiary care hospitals and 30 primary care clinics	All age groups/ ≤5y, 6y-15y, 16y-64y, ≥65y	44	Blood (42), CSF (2)
**Mokaddas E. & Albert M.J., 2012**	65 months: August 2006 through December 2011	Kuwait	Several university and non-university hospitals	All age groups/ <2y, 2-5y, 6-50y, 51-65y, >65y	129	Blood (116), CSF (13)

**Uwaydah M. et al., 1996**	21 months: 19 November 1991-12 August 1993	Lebanon (5,773,493)	1 university hospital	Not specified	24	Blood (17), CSF (7)
**Araj G.F et al., 1999**	24 months: October 1996 - September 1998	Lebanon	1 university hospital in Beirut	All age groups	27	Blood (20), CSF (5), pleural fluid (1), abscess (1)
**Uwaydah M. et al., 2006**	6 months: Dec 2000 till May 2001	Lebanon	3 university hospitals	Not specified	22	Blood (16), CSF (6)
**Hanna-Wakim R. et al., 2012**	75 months: October 2005 - December 2011	Lebanon	78 university hospitals	All age groups: <2y, 2y-5y, 6y-20y, 21y-60y, >60y	257	Blood (201), CSF (35), pleural fluid (9), middle ear fluid (3), mastoid abscess (2), eye discharge (2), urine (2), peri-sternal abscess (1), peritoneal fluid (1), and synovial fluid (1)
**Moghnieh R. et al., 2019**	120 months: Jan 2006 -Dec 2015	Lebanon	1 university hospital	Adults ≥18y	37	Blood, CSF, pleural fluid, or joint fluid
**Borg M.A et al., 2008**	36 months: 2003 to 2005	Mediterranean region (in Algeria, Cyprus, Egypt, Jordan, Lebanon, Malta, Morocco, Tunisia and Turkey)	59 participating laboratories	All age groups	1298	Blood and spinal fluid cultures
**Benbachir M. et al., 2012**	132 months: January 1998 to December 2008	Morocco (37,712,505)	1 University hospital	All age groups/ ≤14y and >14y	531	Blood (172), CSF (271), other invasive biological fluids (88)
**ElMdaghri N., 2012**	12 months: September 2007 and August 2008	Morocco	1 university hospital	Pediatric/ Children ≤5y	24	Blood, CSF, and pleural fluid
**ElMdaghri N., 2012**	96 months:1994–2001 and 48 months: 2006–2010	Morocco	1 university hospital	Pediatric/ Children ≤5y	187	Blood (65), CSF (100), pleural fluid (11), articular fluid, ascitis fluid and pus (11)

**Al-Yaqoubi M.M. & Elhag K. M., 2011**	72 months: September 2002 - December 2007	Oman (5,049,269)	1 university hospital	All age groups/ ≤2y, 2y-5y, 6y-20y, 21y-59y, ≥60y	34	Blood (30), CSF (4)
**Mastro T. D. et al., 1991**	36 months: 1986-1989	Pakistan (247,504,495)	2 large urban hospitals in Islamabad and Rawalpindi	Children <5 y	87	Blood (87)
**Sadia S. et al., 2014**	96 months: from 2005 to 2013	Pakistan	1 University hospital	All age groups: 0-59 months, 5-15 years, 18-70 years	111	CSF (81), Blood (23), Pleural fluid (2), Ascitic fluid (3), synovial fluid (1), unknown sterile body fluid (1)
**Riaz A. et al., 2019**	45 months: July 2013 to March, 2017	Pakistan	16 hospitals	Pediatric/ mean age: 6.5 ± 4.1y	92	CSF
**Zafar A. et al., 2021**	24 years: 1993-2016	Pakistan	1 university hospital	All age groups	2158	Blood, CSF, synovial fluid, pus from sterile body sites, deep tissue, and wounds
**Kattan R. et al., 2011**	111 months: January 2001–April 2010	Palestinian Territories (5,165,775)	2 university hospitals	Pediatric/ range from 1 day to 11 years (71.7% were <2 years of age)	120	Blood
**Al Khal A.L. et al, 2007**	10 months: September 1999 and July 2000	Qatar (2,656,032)	Non-university hospitals	Children <2 and >2 and adults age 40-80	8	Blood (7), CSF (1)
**Elshafie, S. & Taj-Aldeen S.J., 2016**	51 months: Jan 2005 - Mar 2009	Qatar	1 university hospital	All age groups/ range: 4m-90y	134	Blood and CSF
**Al-Swailem A. et al., 2004**	10 months: October 2001 and July 2002	Saudi Arabia (33,264,292)	Laboratories at several hospitals in Riyadh	Not specified	89	Blood (75), CSF (14)
**Memish Z.A. et al., 2004**	12 months: January–December 2000	Saudi Arabia	3 major hospitals	All age groups	51	Blood (27), CSF (24)
**Al-Mazrou A. et al., 2005**	20 months: February 2000 and November 2001	Saudi Arabia	8 university hospitals in Riyadh	All age groups	86	Blood (71), CSF (11), peritoneal fluid (1), synovial fluid (2), bone (1)

**Al Tawfiq J.A., 2004**	48 months: January 1999 - December 2002	Saudi Arabia	Saudi Aramco Medical Services Organization (SAMSO)	All age groups/ mean age: 30.6 ± 30.3y Range: 2 months - 90 years	61	Blood (57), CSF (4)
**Al-Tawfiq J. A., 2006**	60 months: January 1999 to December 2004	Saudi Arabia	Privately contracted hospital (Saudi Aramco Organization)	Pediatrics/ range: 1 month to 17 years with a mean (±SD) of 5.2 (±4.8) years	50	Blood (50)
**Shibl A. M., 2008**	48 months: 2000-2004	Saudi Arabia	25 university hospitals	Pediatric/ Children<2y, 2y->5y, and 5y	350	Blood (287), CSF and other sterile sites (63)
**Shibl, A. M., et al.,2012**	72 months: 2005-2010	Saudi Arabia	Twenty-two hospital clinical laboratories representing the three main regions within KSA	Children<5y	311	Blood (250) and CSF (61)
**Qadri S.M. & Kroschinsky R., 1991**	12 months	Saudi Arabia	1 university hospital	Not specified	52	Blood (49), CSF (3)
**Chowdhury M.N.H., et al., 1995**	10 months: 18 March 1994 to 17 January 1995	Saudi Arabia	1 university hospital	All age groups/ 122 children and 28 adults	27	Blood (22), CSF (3), joint fluids (2)
**Kambal, A. et al., 1997**	49 months: between 1 January 1991 and 31 December 1995	Saudi Arabia	1 university hospital	Pediatrics <14 years old/ 1-12m,13-24m, 25-48m, >49m	49	Blood
**Al-Aqeeli A. A. et al., 2002**	48 months: January 1995 to December 1999	Saudi Arabia	teaching university hospital in Riyadh	All age groups / 3m-11 months, 12m-24 m, 25m-60 m, 61m-14 y, 15y-60y, >60y	172	Blood (172)
**Fouda S. I. et al., 2004**	12 months: 2001-2002	Saudi Arabia	5 laboratory hospitals in Riyadh	Not specified	107	Blood and CSF
**Marzouk M. et al., 2015**	84 months: 2007-2013	Tunisia (12,200,431)	1 university hospital	All age groups/ mean age for adults: 64.3 mean age for children: 32 months	108	Blood (44), CSF (39), pleural fluid (18), other punctures (7)

**Smaoui H. et al., 2009**	84 months: 1998-2004	Tunisia	1 general hospital in Tunisia	Children <2 y	106	blood (40), CSF (52), and articular and pleural fluids (14)
**Charfi F. et al., 2012**	120 months: January 2000–December 2009	Tunisia	1 university hospital	Pediatric/ <2y, 2-5y, >5y (range: 0-16y)	200	Blood (73), CSF (99), other punctions including pleural punction, arthritis, and osteomyelitis (28)
**Raddaoui A., et al., 2015**	84 months: 2005-2011	Tunisia	1 University hospital in Tunisia	All age groups: <6y :10.2%, 6y-18y: 40.7%, >18y:42.4%	9	Blood (9)
**Ktari S. et al., 2017**	60 months: Jan 2012- Dec 2016	Tunisia	1 university hospital	All age groups/ mean age: 29.3 years (range 4 days to 85 years) distribution: <2, 2–4, 5–17, 18–65, >65	73	Blood (38), CSF (28), puncture fluid (Articular, pleural, and ascites fluid samples) (6), and abscess (1)
**Haddad-Boubaker S. et al., 2020**	42 months: January 2014-June 2017	Tunisia	1 governmental hospital	Children aged from 1 month to 15 years old	44	CSF
**Ktari S. et al., 2023**	84 months: 2012-2018	Tunisia	1 university hospital	All age groups	106	Blood (57), CSF (31), puncture fluid (10), deep pus (4), and abscesses (4)
**Percin D. et al., 2010**	115 months: between January 1998 and July 2007	Turkey (85,325,965)	1 University hospital	All age groups: 2, 3-5, 6-15, adult	332	Blood (109), CSF (169), Pleural fluid (15), Peritoneal fluid (37), joint fluid specimens (2)
**Ceyhan M. et al., 2010**	24 months: 2005 - 2007	Turkey	13 medical centers	Children/ <2y and ≥2y (range not specified)	31	CSF (31)
**Ceyhan M. et al., 2011**	18 months: July 2008 - February 2010	Turkey	15 different health centers in Turkey	Pediatrics (≤18y)	202	Blood, CSF and other body fluids
**Altun B. et al., 2006**	48 months: 1999-2002	Turkey	13 centers from different regions of Turkey	All age groups	218	Blood (91), CSF (59), BAL (17), Tracheal Aspirate (20), Pleural fluid (23), Peritoneal fluid (8)
**Altun H.U. et al., 2015**	156 months: 1996 and 2008	Turkey	2 university hospitals in Ankara	All age groups	182	CSF (32) and blood (150)
**Gur D. et al., 2001**	48 months: 1996 - 1999	Turkey	4 university hospitals	All age groups/ Children (303, 44.7%) Adults (375, 55.3%)	124	Blood (84), CSF (40)

**Post-PCV13 introduction**						
**Diawara I., 2017**	84 months: 2007 to 2014	Morocco	1 university hospital	Pediatric/ 0 to 14 years	80	35 from blood (35), 32 from CSF (32), pleural fluid (3), and sterile body fluids (10)
**Ikken Y. et al., 2020**	48 months: 2015-2018	Morocco	12 hospitals	All age groups	65	CSF
**Chikhaoui A. et al., 2022**	48 months: 1st January 2015-31st December 2018	Morocco	1 university hospital	0 to 14 years old	83	Blood (40), CSF (34), ascites (5), pleural fluid (2), peritoneal fluid (1), and ear pus (1)
**A-Jardani A. et al., 2019**	36 months: June 2014 - June 2016	Oman	14 regional and tertiary care hospitals	All age groups: ≤5y, 6y-50y and ≥65y	132	Blood (122), CSF (8), other body fluids (2)
**Al-Sherikh Y. A., et al., 2014**	48 months: between 2009 and 2012	Saudi Arabia	1 University hospital	Children <15y	78	Blood (69), CSF (6), peritoneal fluid (1), synovial fluid (1), and pleural fluid (1)
**Ozdemir H. et al., 2017**	84 months: Sep 2009 - Sep 2015	Turkey	1university hospital	Pediatric/ 1 month-18 years	39	Blood (27), CSF (5), Abscess (2), pleural fluid (1), peritoneal fluid (1), CSF & Blood (3)
**Hascelik G. et al., 2023**	48 months: 2015-2018	Turkey	21 university hospitals	≥ 18 years	252	Blood (191), CSF (38), pleural fluid (17), peritoneal flui (4), paracentesis fluid (1), and vitreous humour (1)
**Kİttana F. N. A., et al., 2019**	Not specified	Turkey	1 university hospital	All age groups	110	Blood (42), CSF (15), pleural fluid (5), cornea (3), peritoneal fluid (1), catheter (1), and BAL (43)
**Ceyhan M. et al., 2020**	48 months: January 2015-December 2018	Turkey	33 hospitals	Children < 18 years old (33% of the cases were under 2 years old and 56% of them were under 5 years old)	167	Blood (104), CSF (52), and Pleural fluid (11)
**Pre- and post-PCV13 introduction**						
**Mokaddas, E. & Albert M. J., 2016**	48 months: August 2003 to July 2006<br>96 months: August 2006 to July 2013	Kuwait	all general hospitals, tertiary-care hospitals, and polyclinics in Kuwait	All age groups/ <2 y, 2–5 y, 6–50 y, 51–65 y, and >65 y	217	Blood (200), CSF (17)

**Reslan L. et al., 2022**	180 months: 2005-2020	Lebanon	79 governmental and university hospitals	All age groups/<br>Mean age: 34.3 ± 32.5 years	593	Blood (502), CSF (74), pleural fluid (21), synovial fluid (1), pericardial fluid (1), mastoid abscess (2), parasternal abscess (1), abscess (1), urine (2), and peritoneal fluid (1). Note: A total of 593 isolates were collected from 606 different sources with 13 isolates identified simultaneously from 2 different sources in the same patient (11 cases from blood and CSF and 2 cases from blood and pleural fluid).
**Diawara I. et al., 2015**	46 months: Jan 2007- Oct 2010 and 48 months: Jan 2011-Dec 2014	Morocco	1 University Hospital	Children ≤2y and >2y-5y	136	Blood, CSF, pleural fluid, and other sterile sites (articular fluid, pus/tissues)
**Nzoyikorera N. et al., 2023**	156 months: 2007-2019	Morocco	1 governmental hospital	≥15 years	250	Blood, CSF, pleural fluid, and other sterile sites (articular fluid, pus/tissues)
**Al-Waili B.R. et al., 2013**	74 months: January 2006 to March 2012	Saudi Arabia	1 university hospital	Pediatric/<br><2y; 2-5y; 5-14y	208	Blood (203), CSF (2), pleural fluid (1), surgical tissues (2)
**Ceyhan M. et al., 2013**	24 months: January 2010 - December 2011	Turkey	13 hospitals	Pediatrics/<br>0 to 15y<br>mean age: 6.17 ± 3.54	55	Pleural fluid
**Ceyhan M. et al., 2016**	77 months in 3 periods: 2008-2010, 2011-2012, 2013-2014	Turkey	22 different hospitals	Pediatric/<br>≤5 and <5- ≤ 18<br>median age: 4 y (interquartile range [IQR], 1.5–9.0)	335	blood, CSF and lung aspirates
**Unspecified period**						
**Haifa Al-Muhtaresh A. & Bindayna K.M., 2020**	Unspecified period	Kingdom of Bahrain	2 governmental hospitals	All age groups	23	Blood (22) and CSF (1)
		**Total population number: 744,051,276**				

Table S3. Serotype Distribution according to Pneumococcal Conjugate Vaccine (PCV) coverage by country in the MENA region (55 original studies up to January 24, 2024)

Author, Year	Country	Total number of invasive isolates with documented serotypes	PCV7 serotypes	PCV13 /non-PCV7 serotypes	PCV15 serotypes	PCV20 serotypes	Non-typeable cases	Other serotypes (N)
**Pre-PCV13 introduction**								
Tali-Maamar et al., 2012	Algeria	49	27	7		2		9N (2); 10 (1); 13 (1); 18 (2); 20 (2); 21 (1); 24 (1); 28 (1); 29 (1); 35 (1)
Hecini-Hannachi A. et al., 2014	Algeria	84	47	13		3	1	19C,9N,12A,16,24F,47F,33F,39,29,21,35B,48 (15); 9A (2); 11 (3)
Ramdani et al., 2015	Algeria	85	45	28		1		6C (1); 9A (1); 12 (1); 16F (2); 17F (1); 23A (1); 23B (1); 28F (1); 35B (1); 35F (1)
Ziane H. et al., 2016	Algeria	80	44	28			2	6C (1); 9N/9L (1); 20 (1); 24F (1); 35B (1); 35F (1)
Guirguis, N. et al., 1983	Egypt	99	5	36		1		2(3); 6(5); 7(3); 9(6); 10(4) ; 11(1);12(4); 15 (1) ; 18(3); 19(3); 20(4); 23(1); 24 (1); 29 (1); 33(1); 34 (2) ; 35 (1); 36(2); 38(1);39(1); 45(4);46(3); 29,42 (1); 29,35,42 (1)
Wasfy M. et al., 2005	Egypt	205	57	51		5	10	2 (4); 7A (1); 9A (2); 11C (1); 15 (3); 15A (1); 15C (1); 16F (6); 17F (1); 20 (5); 7B,7C,23B,9N,11A,15F,18A,22A,33C,35,37,10B,10F,12A,15B,22F (57)
Tavana A. et al., 2013	Iran	133	16	21		8		2 (10); 6 (24); 7 (10); 10 (2); 17 (4); 18 (2); 19 (14); 20 (11); 22 (3); G (8)
Talebi M. et al., 2016	Iran	15	14	1				
Houri H. et al., 2017	Iran	53	33	6		4	3	6A/6B (2); 15A (2); 34 (1); 31 (1); 35B (1)
Abdoli S. et al., 2020	Iran	106	50	24			3	6A/6B (14); 7F/7A (2); 9V/9A (5); 15A/15F (1); 15B/15C (2); 11A/D/F, 22A/F (5)
Ghahfarokhi et al., 2020	Iran	38	19	11				NVT (8)
Azimian A. et al., 2020	Iran	51	17	18			1	6A/B (9); 15A (1); 15B/C (5)
Beheshti M. et al., 2020	Iran	44	12	8		4	3	6A/B (8); 15A (7); 15B/C (2)

Esteghamati A. et al., 2021	Iran	54	24	9		12		16F (2); 31 (2); 34 (3); 35B (1); 35F (1)
Tabatabaie SR. et al., 2021	Iran	83	28	27		3	16	7A (1); 15A (3); 6C (2); 23A (1); 34 (1); 35B (1)
Mosadegh M. et al., 2022	Iran	53	34	9				6A/6B (3); NVT (7)
Tabatabaei SR et al., 2022	Iran	19	2	3		1	4	3 & 11A (5); 3 & 5A (1); 7C & 14 (1); 6A & 7C (1); 19F & 23B (1)
Abu-Helalah M. et al., 2023	Jordan	23	14	7				28 (1); unspecified serotype (1)
Mokaddas E.M. et al., 2008*	Kuwait	44	22	13		2		11C (1); 9A (1); 15A (3); 17F (1); 20 (1)
Mokaddas E. & Albert M.J., 2012	Kuwait	129	The predominant serotypes (identified in >5 isolates) were in descending order: 19F, 6A, 8, 9V, 19A, 1 and 14				9	
Hanna-Wakim R. et al., 2012**	Lebanon	256	75	65	13	12	16	6 (23); 9N (5); 9V/9A (13); 11A/11D (5); 15A (3); 15B/15C (3); 16F (3); 23A (2); 29 (2); 35B (2); 38 (2); 12 other unspecified serotypes
Moghnieh, et al.,2019	Lebanon	24	3	8	2		3	9V/9A (1); 10 (2); 15B/15C (1); 18 (1); 16F (2); 29 (1)
ElMdaghri N. et al., 2012	Morocco	23	14	7				NVT (2)
ElMdaghri N. et al., 2012	Morocco	187	64	49		3	10	2 (5), 7 (10), 6 (8), 9 (1), 9A(1), 10 (3), 11 (3), 15 (5), 18 (4), 18F (1), 19 (5), 22 (1), 23 (7), 24 (2), 25 (1), 27 (1), 33 (1), 38 (1), 40 (1)
Al-Yaqoubi M. et al., 2011	Oman	34	17	11		4	1	9A (1)
Mastro T.D. et a;, 1991	Pakistan	87	38	18				9A (1), 15C (4), 16 (12), 18A (1), 31 (13)
Sadia S. et al., 2014	Pakistan	111	29	18		3	7	6A/6B/6C (2); 7F/7A (1); 9V/9A (3); 9N/9L (1); 10F/10C/33 (2); 11A/11D (3); 12F/A/44/46 (6); 13 (2); 15B/15C (3); 17 (1); 18A/B/C/F (14); 22A/22F (2); 23A (2); 23B (6); 24A/B/F (2); 33F/A/37 (1); 35B (2); 38/25F/25A (1)
Riaz A. et al., 2019	Pakistan	92	15	5			32	6A/6B/6C/6D (1); 7F/7A (1); 18A/B/C/F (2);<br>36 unspecified serotypes
Kattan R. et al., 2011	Palestinian Territories	120	44	36	2	6	3	6A/B (17), Sg18 (5), 16F (2), 17F (1), 35B (3), 38F (1)

Al Khal A. et al., 2007	Qatar	8	2	2	1			16F (1); 40 (1); 48 (1)
El-Shafie S. et al., 2016	Qatar	134	44	49	2	7	12	6A/6B (2); 6C (1); 7C (2); 9A (1); 15A (1); 18 (2); 18F (2); 23A (1); 24F (1); 31 (1); 34 (2); 35B (3); G (1)
Al Swailem A. et al., 2004	Saudi Arabia	89	50	16			6	The prevalence of other serotypes (22F, 2, 3, 4, 7F, 8, 10A, 11A, 12F, 20, and nonvaccine serotype from pool sera D and E) ranged from 1 (1.1%) to 3 (3.4%).
Memish Z. et al., 2004	Saudi Arabia	50	22	9		1	2	22F, 23B, 9N, 15B, 19C (10); 7A (3); 17F (3)
Al-Mazrou A. et al. 2005	Saudi Arabia	79	9	12			5	9 (1); 13 (1); 6 (12); 19 (12); 15 (8); 23 (6); 7 (3); 18 (3); 22 (4); 10 (1); 11 (1); 12 (1)
Shibl A. et al., 2008	Saudi Arabia	350	217	29		6	51	7 (6), 11 (6), 12 (7), 15 (6), 22 (4), 23A (6), 23B (5), 24 (7)
Shibl A. et al., 2012	Saudi Arabia	108	58	44				6 other unspecified serotypes
Smaoui H. et al., 2009	Tunisia	58	58					
Charfi F. et al., 2012	Tunisia	186	127	17		4		2, 12, 20, 33 and NT (36); 10 (1); 15B/15C (1)
Raddaoui A. et al., 2015	Tunisia	9	5	1				23 (1); 31 (1); 35 (1)
Haddad-Boubaker S. et al., 2020	Tunisia	34	26	3		1		6A/B (2); 18 (2)
Marzouk M. et al., 2015	Tunisia	108	37	32			28	9 (2); 19B (7); 23 (2)
Ktari S. et al., 2017***	Tunisia	73	40	16		1	3	6C (1); 7C (1); 9V/A (5); 17F (2); 34 (1); 35B (2); 35F (1)
Ktari S. et al., 2023	Tunisia	106	59	27				6C (1); 7C (1); 9A (3); 9N (3); 13 (1); 16F (2); 17F (3); 24F (1); 34 (1); 35B (3); 35F (1)
Altun H. et al., 2015	Turkey	66	21	13		1	15	20 (5); 15 (3); 7A (4); 11 (3); 17 (1)
Percin et al., 2010	Turkey	332		95				1,2,3,4,5,6,7,8,9,10,11,12,14,15,18,20,22,23, NT (175); 19 (62)
Ceyhan M. et al., 2010	Turkey	31	12	13		1	4	7 (1)
Ceyhan M. et al., 2011****	Turkey	202	96	38		5	13	2 (2); 6 (1); 15 (5); 15C (5); 23A (1); 7A (5); 10 (2); 16F (1); 17 (1); 27 unspecified serotypes

**Post-PCV13 introduction**								
Diawara I. et al., 2017	Morocco	80	42	22	1		11	7A (2); 23A (1); 24 (1)
Al-Jardani et al., 2019	Oman	132	21	28		3	6	6C (1); 6D (1); 7F/7A (1); 7B/7C (1); 9A (3); 9N/9L (4); 10B (2); 10F/10C (1); 11A/11D (3); 11F/11B/11C (3); 12 (11); 13 (2); 15 (11); 16F (3); 17F (4); 18A/18B (2); 19 (1); 22 (7); 23A (3); 23B (1); 24 (1); 28 (1); 29 (1); 34 (1); 35B (3); 37 (1); 39 (1)
Al-Sherikh et al, 2014	Saudi Arabia	77	57	9		2		24 (1); 15 (2); 23A (2); 23B (1); 7 (1); 22 (1); 11 (1)
Ozdemir et al., 2017	Turkey	39	10	13		2	3	10 (1); 15 (1); 15A/15F (1); 15A/15F/15B/15C (1); 18F (1); 12B (1); 17F/17A (1); 21 (1); 33F/A/B/C/D (1); 35A/B/C (1); 35B (1)
Kittana F.N.A. et al., 2019	Turkey	107	57	29	2	4		35F (3); 16B (1); 6C (1); 15A (2); 6 (1); 6D (1); 7 (1); 11B (1); 6B/D (1); 12 (1); 15 (1); 23B (1)
Ceyhan M. et al., 2020	Turkey	167	42	52		24		15C (3); 15F (3); 20 (3); and others (40)
Hascelik G. et al., 2023	Turkey	193		166	7	17		3 other unknown serotypes covered by PPSV23; unknown number of NVT cases (35F, 15A, 18F, and 11C)
**Pre- and post-PCV13 introduction**								
Mokaddas, E. & Albert M. J., 2016	Kuwait	212	72	54	2	36	8	33D (5), 2 (3), 7C (1), 9A (1), 22A (2), 9N (1), 15C (3), 15F (5), 9L (3), 12B (2), 19C (1), 23A (3), 15A (2), 18A (1), 20 (4),17F (2), 33A (1)
Reslan L. et al., 2022	Lebanon	549	182	166	25	45	32	2 (6); 6C (2); 7C/7B/7B/40F (2); 9N (12); 10B (2); 10F/10C/33 (3); 13 (4); 15A/15F (4); 16F (9); 17F (2); 20 (2); 21 (2); 23A (8); 23B (5); 24F (12); 28A (1); 29 (2); 31 (5); 34 (4); 35A/35C/42 (2); 35B (2); 35F/47 (3); 38/25 (3); 39 (1); 42 (1)
Diawara I. et al., 2015	Morocco	136	62	41	1	4	15	11A/11E (1), 15A (1); 18F (3); 10F (2); 2 (3); 7A (1); 24F (1); 23B (1)
Nzoyikorera N. et al., 2023	Morocco	239	39	59	3	28		2 (1); 7A (3); 7C (1); 10 (3); 11 (3); 17F (6); 20 (1); 23A (4); 33 (4); 34 (5); 9N (3); 35F (1); NVT (75)
Ceyhan M. et al., 2013****	Turkey	55	9	22		3	20	7 (1)
Ceyhan M. et al., 2016	Turkey	335	138	70		8	22	2 (3); 6 (1); 7A (5); 10 (4); 15 (7); 15C (9); 16F (1); 17 (1); 23A (3); Other serotypes (63)

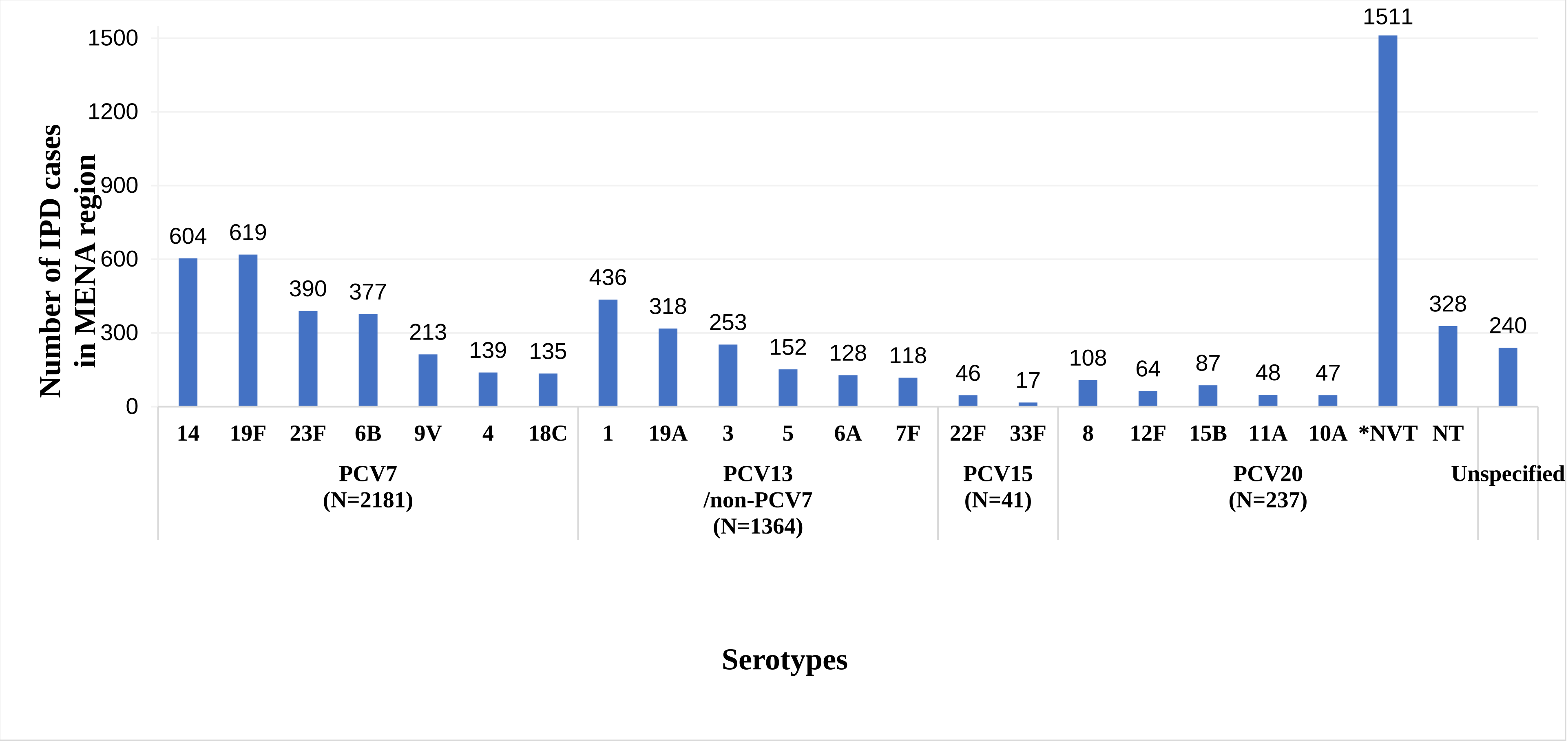

Figure S1. Overall Serotype distribution in the MENA region (N=5902 IPD cases) (55 original studies up to January 24, 2024)

NVT (non-vaccine type): 2 (33);); 6C (6); 6D (2); 7A (21); 7B/7C (1); 7C (4); 9 (10); 9N (8); 9N/9L (6); 9L (3);10B (2); 10F (2); 10F/10C (1); 10F/10C/33 (2); 11A/11D (11); 11A/11E (1); 11B (1); 11F/11B/11C (3); 11C (2); 12B (3); 12F/A/44/46 (6);13 (6);15C (22); 15A (15); 15A/15F (2); 15A/15F/15B/15C (1); 15F (5); 15B/15C (10); 16B (13); 16F (21); 17 (4); 17F (19); 17F/17A (1); 18A (2); 18A/18B (2); 18A/B/C/F (16); 18F (7); 19B (8); 19C (1); 20 (33); 21 (2); 22A (2); 22A/22F (2); 23A (25); 23B (16); 24 (14); 24A/B/F (2); 24F (3); 25 (1); 27 (1); 28 (2); 28F (1); 29 (6); 29,42 (1); 29,35,42 (1); 31 (16); 33A (1); 33D (5); 33F/A/37 (1); 33F/A/B/C/D (1); 34 (7); 35 (3); 35A/B/C (1); 35B (21); 35F (6); 36 (2); 37 (1); 38(4); 38/25F/25A (1); 38F (1); 39 (2); 40 (2); 45(4); 46(3); 48 (1); G (9).

*Studies where there was no distinction between serogroup 6 sub-serotypes were included under 6B, same for 7 and 7F, 9V and 9V/A,10A and 10,11A and 11,12F and 12,15 and 15B, 18C and 18,19 and 19F,22 and 22,23 and 23F,33 and 33F.

NT: Non-typeable isolates; Unspecified serotypes: serotypes that were typed but not reported, hence we could not include them within any category.

Table S4. Serotype Distribution by country in the MENA region (59 original studies up to January 24, 2024)

Author, Year	Country	Total number of invasive isolates with documented serotypes	4	6B	9V	14	18C	19F	23F	1	5	7F	3	6A	19A	22F	33F	8	10A	11A	12F	15B	Non-typeable	Other serotypes (N)
**Pre-PCV13 introduction**																								
**Tali-Maamar et al., 2012**	**Algeria**	**49**		3		15		4	5	2	1	1	1	1	1			2						9N (2); 10 (1); 13 (1); 18 (2); 20 (2); 21 (1); 24 (1); 28 (1); 29 (1); 35 (1)
**Hecini-Hannachi A. et al., 2014**	**Algeria**	**84**	2	5		17	3	13	7	3		3	3	1	3				3				1	19C,9N,12A,16,24F,47F,33F,39,29,21,35B,48 (15); 9A (2); 11 (3)
**Ramdani et al., 2015**	**Algeria**	**85**	1	6		25	1	9	3	9	4	2	4	3	6			1						6C (1); 9A (1); 12 (1); 16F (2); 17F (1); 23A (1); 23B (1); 28F (1); 35B (1); 35F (1)
**Ziane H. et al., 2016**	**Algeria**	**80**		6		26	1	8	3	6	6	1	3		12								2	6C (1); 9N/9L (1); 20 (1); 24F (1); 35B (1); 35F (1)
**Guirguis, N. et al., 1983**	**Egypt**	**99**	3			2				34	1		1					1						2(3); 6(5) ; 7(3); 9(6); 10(4) ; 11(1);12(4); 15 (1) ; 18(3); 19(3); 20(4); 23(1); 24 (1); 29 (1); 33(1); 34 (2) ; 35 (1); 36(2); 38(1);39(1); 45(4);46(3); 29,42 (1); 29,35,42 (1)
**Wasfy M. et al., 2005**	**Egypt**	**205**	3	21		8	4	8	13	15	5	1	4	12	14			2	3				10	2 (4); 7A (1); 9A (2); 11C (1); 15 (3); 15A (1); 15C (1); 16F (6); 17F (1); 20 (5); 7B,7C,23B,9N,11A,15F,18A,22A,33C,35,37,10B,10F,12A,15B,22F (57)
**Tavana A. et al., 2013**	**Iran**	**133**	8			8				10	7		4					8						2 (10); 6 (24); 7 (10); 10 (2); 17 (4); 18 (2); 19 (14); 20 (11); 22 (3); G (8)

**Houri H. et al., 2017**	**Iran**	**53**	2		4	3	1	10	13	1		1			4			1		3			3	6A/6B (2); 15A (2); 34 (1); 31 (1); 35B (1)
**Talebi M. et al., 2016**	**Iran**	**15**			1	10		2	1				1											
**Abdoli S. et al., 2020**	**Iran**	**106**				9	4	17	20	11					13								3	6A/6B (14); 7F/7A (2); 9V/9A (5); 15A/15F (1); 15B/15C (2); 11A/D/F, 22A/F (5)
**Ghahfarokhi et al., 2020**	**Iran**	**38**		1		6		3	9				4		7									NVT (8)
**Azimian A. et al., 2020**	**Iran**	**51**				5		5	7	8					10								1	6A/B (9); 15A (1); 15B/C (5)
**Beheshti M. et al., 2020**	**Iran**	**44**			2	2		2	6	1					7					4			3	6A/B (8); 15A (7); 15B/C (2)
**Esteghamati A. et al., 2021**	**Iran**	**54**		9	2	7		3	3	4		2	1		2			1		3	2	6		16F (2); 31 (2); 34 (3); 35B (1); 35F (1)
**Tabatabaie SR. et al., 2021**	**Iran**	**83**	3		4	1		9	11	1			18	4	4					3			16	7A (1); 15A (3); 6C (2); 23A (1); 34 (1); 35B (1)
**Mosadegh M. et al., 2022**	**Iran**	**53**			4	13		10	7				8		1									6A/6B (3); NVT (7)
**Tabatabaei SR et al., 2022**	**Iran**	**19**				1			1				1	1	1					1			4	3 & 11A (5); 3 & 5A (1); 7C & 14 (1); 6A & 7C (1); 19F & 23B (1)
**Abu-Helalah M. et al., 2023**	**Jordan**	**23**		2		7	2	3		1			2	1	3									28 (1); unspecified serotype (1)
**Mokaddas E.M. et al., 2008***	**Kuwait**	**44**	2	2	4	7		2	5	3	2		1	3	4				2					11C (1); 9A (1); 15A (3); 17F (1); 20 (1)
**Mokaddas E. & Albert M.J., 2012**	**Kuwait**	**129**	The predominant serotypes (identified in >5 isolates) were in descending order 19F, 6A, 8, 9V, 19A, 1 and 14																				9	
**Hanna-Wakim R. et al., 2012****	**Lebanon**	**256**	9			18	8	31	9	16	10	6	18		15	7	6	4	4		4		16	6 (23); 9N (5); 9V/9A (13); 11A/11D (5); 15A (3); 15B/15C (3); 16F (3); 23A (2); 29 (2); 35B (2); 38 (2); 12 other unspecified serotypes
**Moghnieh, et al.,2019**	**Lebanon**	**24**	1					2		1		1	3		3		2						3	9V/9A (1); 10 (2); 15B/15C (1); 18 (1); 16F (2); 29 (1)
**ElMdaghri et al., 2012**	**Morocco**	**23**		3		3	1	4	3	2	1		1		3									NVT (2)
**ElMdaghri et al., 2012**	**Morocco**	**187**	1	11	3	25	3	10	11	15	19	3	4		8			3					10	2 (5), 7 (10), 6 (8), 9 (1), 9A(1), 10 (3), 11 (3), 15 (5), 18 (4), 18F (1), 19 (5), 22 (1), 23 (7), 24 (2), 25 (1), 27 (1), 33 (1), 38 (1), 40 (1)

**Al-Yaqoubi M. et al., 2011**	**Oman**	**34**	2	4		4		4	3	5	2	1		1	2				2	1	1		1	9A (1)
**Mastro T.D. et a;, 1991**	**Pakistan**	**87**		1	9			28		1	2			4	11									9A (1), 15C (4), 16 (12), 18A (1), 31 (13)
**Sadia S. et al., 2014**	**Pakistan**	**111**	2			9		13	5	8	8		1		1			1	2				7	6A/6B/6C (2); 7F/7A (1); 9V/9A (3); 9N/9L (1); 10F/10C/33 (2); 11A/11D (3); 12F/A/44/46 (6); 13 (2); 15B/15C (3); 17 (1); 18A/B/C/F (14); 22A/22F (2); 23A (2); 23B (6); 24A/B/F (2); 33F/A/37 (1); 35B (2); 38/25F/25A (1)
**Riaz A. et al., 2019**	**Pakistan**	**92**				4		7	4	2	3												32	6A/6B/6C/6D (1); 7F/7A (1); 18A/B/C/F (2);<br>36 unspecified serotypes
**Kattan R. et al., 2011**	**Palestinian Territories**	**120**	6		11	16		8	3	14	9	4	4		5		2	4			2		3	6A/B (17), Sg18 (5), 16F (2), 17F (1), 35B (3), 38F (1)
**Al Khal A. et al., 2007**	**Qatar**	**8**		1					1			1			1	1								16F (1); 40 (1); 48 (1)
**El-Shafie S. et al., 2016**	**Qatar**	**134**	4	3	7	16	1	6	7	15	3	1	17	1	12	1	1	3			4		12	6A/6B (2); 6C (1); 7C (2); 9A (1); 15A (1); 18 (2); 18F (2); 23A (1); 24F (1); 31 (1); 34 (2); 35B (3); G (1)
**Al Swailem A. et al., 2004**	**Saudi Arabia**	**89**		13	10	6	4	5	12						16								6	The prevalence of other serotypes (22F, 2, 3, 4, 7F, 8, 10A, 11A, 12F, 20, and nonvaccine serotype from pool sera D and E) ranged from 1 (1.1%) to 3 (3.4%).
**Memish Z. et al., 2004**	**Saudi Arabia**	**50**	4		5	4		6	3	1	1	1	2	2	2			1					2	22F, 23B, 9N, 15B, 19C (10); 7A (3); 17F (3)
**Al-Mazrou A. et al. 2005**	**Saudi Arabia**	**79**	3			6				8	1		3										5	9 (1); 13 (1); 6 (12); 19 (12); 15 (8); 23 (6); 7 (3); 18 (3); 22 (4); 10 (1); 11 (1); 12 (1)
**Shibl A. et al., 2008**	**Saudi Arabia**	**350**	11	36	18	55	20	31	46	5	5		7	6	6			6					51	7 (6), 11 (6), 12 (7), 15 (6), 22 (4), 23A (6), 23B (5), 24 (7)
**Shibl A. et al., 2012**	**Saudi Arabia**	**108**	3	8	6	6		14	21	9	9	9	2	7	8									6 other unspecified serotypes
**Smaoui H. et al., 2009**	**Tunisia**	**58**	8	5	4	29	1	2	9															
**Charfi F. et al., 2012**	**Tunisia**	**186**	16	12	12	55	2	12	18	4	4	2	1		6			4						2, 12, 20, 33 and NT (36); 10 (1); 15B/15C (1)
**Raddaoui A. et al., 2015**	**Tunisia**	**9**		2	1	2						1												23 (1); 31 (1); 35 (1)

**Haddad-Boubaker S. et al., 2020**	**Tunisia**	**34**		4	1	9		8	4					1	2						1			6A/B (2); 18 (2)
**Marzouk M. et al., 2015**	**Tunisia**	**108**	2	2		31	2			25			3	2	2								28	9 (2); 19B (7); 23 (2)
**Ktari S. et al., 2017***	**Tunisia**	**73**	2	4		14	4	12	4	1	1	1	4	5	4					1			3	6C (1); 7C (1); 9V/A (5); 17F (2); 34 (1); 35B (2); 35F (1)
**Ktari S. et al., 2023**	**Tunisia**	**106**	2	8	6	18	6	15	4	1		1	12	8	5									6C (1); 7C (1); 9A (3); 9N (3); 13 (1); 16F (2); 17F (3); 24F (1); 34 (1); 35B (3); 35F (1)
**Percin et al., 2010**	**Turkey**	**332**								95														1,2,3,4,5,6,7,8,9,10,11,12,14,15,18, 20,22,23,NT (175); 19 (62)
**Ceyhan M. et al., 2010**	**Turkey**	**31**		2	1	1		5	3	3	7			2	1			1					4	7 (1)
**Ceyhan M. et al., 2011*****	**Turkey**	**202**	14	16	2	12	5	39	8	8	4	1	10	5	10			5					13	2 (2); 6 (1); 15 (5); 15C (5); 23A (1); 7A (5); 10 (2); 16F (1); 17 (1); 27 unspecified serotypes
**Altun H. et al., 2015**	**Turkey**	**66**		3	5	3	1	3	6	1	4	1		5	2			1					15	20 (5); 15 (3); 7A (4); 11 (3); 17 (1)
**Post-PCV13 introduction**																								
**Diawara I. et al., 2017**	**Morocco**	**80**		5	1	23	1	6	6		1	1		2	18	1							11	7A (2); 23A (1); 24 (1)
**Al-Jardani et al., 2019**	**Oman**	**132**	1	3		1	4	10	2	5	1	5	8	1	8			2	1				6	6C (1); 6D (1); 7F/7A (1); 7B/7C (1); 9A (3); 9N/9L (4); 10B (2); 10F/10C (1); 11A/11D (3); 11F/11B/11C (3); 12 (11); 13 (2); 15 (11); 16F (3); 17F (4); 18A/18B (2); 19 (1); 22 (7); 23A (3); 23B (1); 24 (1); 28 (1); 29 (1); 34 (1); 35B (3); 37 (1); 39 (1)
**Al-Sherikh et al , 2014**	**Saudi Arabia**	**77**	7	11		4	8	9	18	1	1		2	2	3			1			1			24 (1); 15 (2); 23A (2); 23B (1); 7 (1); 22 (1); 11 (1)
**Ozdemir et al., 2017**	**Turkey**	**39**				1		5	4	1	1	4	2	3	2			2					3	10 (1); 15 (1); 15A/15F (1); 15A/15F/15B/15C (1); 18F (1); 12B (1); 17F/17A (1); 21 (1); 33F/A/B/C/D (1); 35A/B/C (1); 35B (1)
**Kittana F.N.A. et al., 2019**	**Turkey**	**107**	1	2		6	1	44	3	4	2	3	3	4	13		2	3				1		35F (3); 16B (1); 6C (1); 15A (2); 6 (1); 6D (1); 7 (1); 11B (1); 6B/D (1); 12 (1); 15 (1); 23B (1)
**Ceyhan M. et al., 2020**	**Turkey**	**167**	4	2	4	4	1	20	7	18	1	0	17	9	7			5	5	3	5	6		15C (3); 15F (3); 20 (3); and others (40)

**Pre- and post-PCV13 introduction**																								
**Mokaddas, E. & Albert M. J., 2016**	**Kuwait**	**212**	3	4	16	14	4	21	10	14	4	3	10	10	13	2		17	3	3	4	9	8	33D (5), 2 (3), 7C (1), 9A (1), 22A (2), 9N (1), 15C (3), 15F (5), 9L (3), 12B (2), 19C (1), 23A (3), 15A (2), 18A (1), 20 (4),17F (2), 33A (1)
**Reslan L. et al., 2022**	**Lebanon**	**549**	16	18	21	39	18	49	21	42	17	11	48	19	29	17	8	6	6	9	12	12	32	2 (6); 6C (2); 7C/7B/7B/40F (2); 9N (12); 10B (2); 10F/10C/33 (3); 13 (4); 15A/15F (4); 16F (9); 17F (2); 20 (2); 21 (2); 23A (8); 23B (5); 24F (12); 28A (1); 29 (2); 31 (5); 34 (4); 35A/35C/42 (2); 35B (2); 35F/47 (3); 38/25 (3); 39 (1); 42 (1)
**Diawara I. et al., 2015**	**Morocco**	**136**		18	3	21	2	10	8	9	9	4	6	3	10	1		3	1				15	11A/11E (1), 15A (1); 18F (3); 10F (2); 2 (3); 7A (1); 24F (1); 23B (1)
**Nzoyikorera N. et al., 2023**	**Morocco**	**239**	3	9	3	4	6	6	8	10	4	5	22	3	15	3		21			7			2 (1); 7A (3); 7C (1); 10 (3); 11 (3); 17F (6); 20 (1); 23A (4); 33 (4); 34 (5); 9N (3); 35F (1); NVT (75)
**Ceyhan M. et al., 2013******	**Turkey**	**55**		2	1	2		3	1	8	7	1	5		1			3					20	7 (1)
**Ceyhan M. et al., 2016**	**Turkey**	**335**	17	20	8	20	6	53	14	11	9	4	20	10	16			8					22	2 (3); 6 (1); 7A (5); 10 (4); 15 (7); 15C (9); 16F (1); 17 (1); 23A (3); Other serotypes (63)

*The results of Mokaddas E.M. et al., 2008 were included in the paper of Mokaddas, E. & Albert M. J., 2016
**The results of Hanna-Wakim R. et al., 2012 were included in the paper of Reslan L. et al., 2022
***The results of Ktari S. et al., 2017 were included in the paper of Ktari S. et al., 2023
****The results of Ceyhan M. et al., 2011 and Ceyhan M. et al., 2013 were included in the paper of Ceyhan M. et al., 2016

Table S5. Serotype Distribution by country and age groups in the MENA region (17 original studies up to January 24, 2024)

Author, Year	Country	Total number of IPD isolates with documented serotypes	Population	Period	Age distribution	Number of isolates /age group	4	6B	9V	14	18C	19F	23F	1	5	7F	3	6A	19A	22F	33F	8	10A	11A	12F	15B	Non-typeable	Other serotypes
**Pre-PCV13 introduction**																												
**Ziane H. et al., 2016**	**Algeria**	**80**	**Pediatric**	**2010-2014 (pre-PCV 7 introduction)**	<1y	44		4		13		2	1	4	5	1	3		6								1	35 F (1), 35 B (1), 24 F (1), 20 (1)
					1y-2y	20		1		6	1	4		1	1				4								1	9N/9L (1)
					3y - 5y	16		1		7		2	2	1					2									6C (1)
**Guirguis, N. et al., 1983**	**Egypt**	**99**	**All age groups**	**1977-1978 (pre-PCV 7 introduction)**	<1 y	24				1				3	1		1											2 (2), 6 (1), 7(2); 9(1); 12(1);

																												18 (1), 20(2); 33(1); 34(2); 35(1); 36(1); 45 (1); 46(2)
					1y - 4 y	16				1				8														6 (2), 7A (1), 9 (1); 12(1); 23(1); 39(1)
					5y - 9y	12	2							7														9(1);19(1);46(1)
					10y - 14y	20								6														6 (2); 9 (1); 10 (1); 15 (1); 18 (1); 19

																												(2); 20(1); 24(1); 36(1); 38(1); 45(2)
					15y - 34y	17	1							7														2 (1); 9(2); 9N (1); 10 (1); 12(1); 20(1); 29(1); 29, 35, 42(1)
					>=35y	10								3									1					7(1); 9N (2); 12(1); 45(1); 29, 42(1)
**Houri H. et**	**Iran**	**53**	**Pedia**	**2013-2016 (Pre-**	0-3 months	9				1		2	3											1			1	15A (1)

**al., 2017**			**tric**	**PCV13 introduction)**	4-24 months	25	1		2	1	1	6	7						2								1	6 (2), 35B (1), 31 (1)
					25-60 months	19	1		2	1		2	3	1		1			2			1		2			1	15A (1), 34 (1)
**Tabatabaei SR et al., 2022**	**Iran**	**19**	**Pediatrics**	**2016-2017 (Pre-PCV introduction)**	≤1y														1								1	3 & 5A (1); 3 & 11A (1)
					2y-4y								1				1							1			2	3 & 11A (2); 6A & 7C (1); 19F & 23B (1)
					5y-10y					1								1									1	3 & 11A (2); 7C & 14 (1)

**Mokaddas E.M. et al., 2012**	**Kuwait**	**129**	**All age groups**	**2006-2011 Post PCV 7 Introduction**	<2y	26	The predominant serotypes in children ≤5 years were 19F, 19A, 6A, 8 and 15B. However, the predominant serotypes in adults >50 years were 14, 3, 1, 19F and 8.																				9	
					2y - 5y	19																						
					6y - 50y	36																						
					51y - 65y	24																						
					>65 y	24																						
**Sadia S. et al., 2014**	**Pakistan**	**111**	**All age groups**	**2005-2013 (Pre PCV 10 introduction)**	0-59 months	85	2			8		7	3	4	7		1					1	2				5	6A/6B/6C (2), 9V/9A (3), 9N/9L (1), 10F/10C (1), 10F/10C/33 (1), 11A/11D (2), 12F/A/44/46 (6), 15B/15C (2), 18A/1

																												8B/18C/18F (13), 22A/22F (1), 23A (2), 23B (6), 33F/A/37 (1), 35B (2), 24A/B/F (1), 17 (1)
					5y - 15y	9						3		1													1	7F/7A (1), 11A/11D (1), 18A/18B/18C/18F (1), 24

																												A/B/F (1)
					18y - 70y	17				1		4	2	3	1												1	13 (2), 15 B/15C (1), 22 A/22F (1), 38/25 F/25A (1)
**El-Shafie S. et al., 2016**	**Qatar**	**134**	**All age groups**	**2005-2009 (Post PCV7/Pre-PCV13 introduction)**	<2y	23		1		5		2		2	1	1	2		4								2	35 B (2), 24 F (1)
					2y - 5y	28		1	1	6	1	2	3	4	1		1		1			1					2	9A (1), 12 F/A/44/46 (1), 18 (1), 35 B (1)
					6y - 64y	58	3	1	4	3		1	4	7	1		11		3	1	1	2					7	6C (1), 7C (1), 12 F/A/44/4

																												6 (2), 18 (1), 18 F (1), 31 (1), 34 (2)
					>64 y	25	1		2	2		1		2			3	1	4								1	G (1), 6A/ 6B (2), 7C (1), 12 F/ A/4 4/4 6 (1), 15 A (1), 18 F (1), 23 A (1)
**Al-Mazrou A. et al., 2005**	**Saudi Arabia**	**71**	**All age groups**	**2000-2001 (pre-PCV7 introduction)**	<15 y	51	2			5				1			2											6 (10), 7 (2), 15 (5), 18 (3), 19 (4), 23 (6)

					≥15 y	20								4			1											6 (1), 15 (3), 19 (6), 22 (2)
					Children (<2 y): 27 isolates included in the 51 isolates for children <15 y	27 isolates included in the 51 isolates for children <15 y	Serotypes 23 and 14 causing 6 (22%) cases.<br>Serogroups/serotypes 3, 4, 6, 14 and 23 were the most common.																					
**Shibl A. et al., 2008**	**Saudi Arabia**	**350**	**Pediatric**	**2000-2004 (Pre PCV 7 introduction)**	<2y	159	5	24	7	35	11	19	31		1		2	1	3								11	7 (1), 11 (1), 15 (2), 22 (1), 23 A (1), 23 B (1), 24 (2)
					2y - <5y	106	4	8	9	18	6	8	13	3	2		1	1	1			1					15	7 (2),

																												11 (4), 12 (1), 22 (3), 23 A (2), 23 B (1), 24 (3)
					5y	85	2	4	2	2	3	4	2	2	2		4	4	2			5					25	7 (3), 11 (1), 12 (6), 15 (4), 23 A (3), 23 B (3), 24 (2)
**Smaoui H. et al., 2009**	**Tunisia**	**58**	**Pediatrics**	**1998-2004 (pre-PCV7 introduction)**	<2y	38	7	4	2	18	1	2	4															
					2y - 16y	20	1	1	2	11	0	0	5															
**Ktari S. et al., 2017**	**Tunisia**	**73**	**All age group**	**2012-2016 (Pre-PCV introduction)**	<2y	25		3		3	3	7	2					2	2								1	9V/A (2)
					2y - 4y	5		1		2							1	1										
					5y - 17y	5				2				1										1			1	

			**ups**		18y-65y	24	1			2	1	4	2		1	1	1	2	1									6C (1); 7C (1); 9V/A (2); 17F (1); 34 (1); 35B (1); 35F (1)
					>65y	14	1			5		1					2		1								1	9V/A (1); 17F (1); 35B (1)
**Ceyhan M. et al., 2010**	**Turkey**	**27**	**Pediatric**	**2005-2007 (Pre-PCV13 introduction)**	<2y	14		2	1	1		4	2	1	2			1										
					>2y	13						1	1	2	5			1	1			1						18 (1)
**Abu-Helalah M. et al., 2023**	**Jordan**	**23**	**Pediatrics**	**2021-2022 (Pre-PCV introduction)**	≤6m	12		1		4	2	1		1			1	1										28 (1)
					7m-12m	4		1		2							1											
					13m-24m	2												1										Other unspecified

																												serotype (1)
					25m - 53m	5				1		2						1	1									
**Pre- and post-PCV13 introduction**																												
**Mokaddas, E. & Albert M. J., 2016**	**kuwait**	**212**	**All age groups**	**Pre-PCV7 vaccination 2003-2006**	<2y	9				2		2	1			1							1				1	33D (1)
					2y - 5y	7		1				2	1	1					1							1		
					6y - 50y	13	1		1			3			1				1			2	1				1	2 (2)
					51y - 65y	25	1		3	3		2	4	3				1	2			1					1	7C (1), 9A (1), 22A (2)
					>65y	9			2	1	1	2		1										1				9N (1)
				**Post-PCV7 vaccination 2006-2010**	<2y	18		1	1		2	3			1			3	1			2				2		15C (1), 15F (1)
					2y - 5y	14			4			3	1	1				1	2							1		15C (1)
					6y - 50y	31			3	1	1		1	3		1	4	1	1			3		2		1		2 (1), 9L (1), 12B (1),

																												15C (1), 15F (2), 19C (1), 23A (2)
					51y - 65y	21	1			4		3	1	4			2					2					2	15A (1), 18A (1)
					>65 y	23		2	1	3				1	2		3	1		1		4			1		1	15F (1), 20 (1), 33D (1)
				**Post-PCV13 vaccination 2010-2013**	<2y	4							1						1							1	1	
					2y - 5y	6													2							1	1	12B (1), 20 (1)
					6y - 50y	12												3								2		9L (2), 17F (1), 20 (1), 33D (3)
					51y - 65y	9						1				1			1			2			3			15F (1)

					>65y	11			1								1		1	1		1	1					15A (1), 17F (1), 20 (1), 23A (1), 33A (1)
**Reslan L. et al., 2022**	**Lebanon**	**543**	**All age groups**	**2005-2009 (PCV7 era)**	≤5y	68	1	8	1	15	2	9	2	1	4		4	1	3		2			1	1	1	6	9N (1); 16F (1); 24F (1); 28A (1); 31 (1); 42 (1)
					6y-60y	48	1	2	2	1	1	9	2	5	1	1	1	2	2	3		2	3			1	2	9N (2); 15A/A5F (2); 16F (1); 29 (1); 38/25 (1)

					>60 y	56	2	4	5		1	5	2	4		3	9		2	5	2	1	1	1	1	2	1	9N (2); 16 F (1); 29 (1); 34 (1)
				**2010-2015 (Post-PCV 7/pre-PCV 13 era)**	≤5y	76	2	1	2	11	3	9	6	8	3	2	2	3	6		1			2	2	1	2	2 (3); 9N (1); 10 F/10C/33 C (2); 16 F (1); 23 A (1); 24 F (1); 35 F/47 (1)
					6y-60y	64	1	2	1	3	2	8	4	6	4	1	6	5	2			1			1	2		2 (1); 6C (1); 9N (1); 10 B (1); 13 (2); 15 A/1

																												5F (1); 16 F (1); 17 F (1); 21 (2); 23 A (2); 35 B (1); 38/ 25 (1)
					>60 y	61	3		6	3	4	4	1	3	2	1	7	1	6	3	2			1	1		3	2 (1); 6C (1); 9N (1); 10 B (1); 16 F (2); 20 (1); 23 A (1); 31 (1); 38/ 25 (1); 39 (1)
				**2016-2020**	≤5y	63	3	1	2	4		1	2	7	2		6	2	3	1	1	1		1	2	1	9	9N (2);

				**(PCV 13 era)**																								16F (1); 23A (2); 23B (1); 24F (5); 34 (1); 35B (1); 35F/47 (1)
					6y-60y	52	1		2		1	1	1	6	1		3	3	2	1		1	1	1	3	2	2	7C/7B/40F (2); 9N (1); 13 (1); 16F (1); 17F (1); 23A (1); 23B (2); 24F (4);

																												31 (2); 34 (2); 35A/35C/42 (2); 35F/47 (1)
					>60y	55	2			2	4	3	1	2		3	10	1	2	4			1	2	1	2	6	2 (1); 9N (1); 10F/10C/33C (1); 15A/15F (1); 23A (1); 23B (2); 24F (1); 31 (1)
**Diawara I. et al., 2015**	**Morocco**	**136**	**Pediatrics**	**Pre-PCV13 introduction (2007**	≤2y	79		11	1	13	2	7	7	4	6	3	4		9	1		1					6	11A/11E (1), 18F (1),

				**- 2010)**																								10 F (1), 24 F (1)
					>2y - 5y	12		1	1			3	1	2	1			1	1			1						
				**Post-PCV 13 and PCV 10 introduction (2011 - 2014)**	≤2y	32		6	1	4				2		1	1	1				1	1				6	2 (3); 15 A (1), 18 F (2), 10 F (1), 7A (1)
					>2y - 5y	13				4				1	2		1	1									3	23 B (1)
**Nzoyikorera N. et al., 2023**	**Morocco**	**239**	**Adults**	**2007-2010 (Pre-vaccine period)**	15y-59y	71		1	1	1	6	2	3	3	1	2	2	2	9			7			3			2 (1); 7A (1); 7C (1); 10 (2); 23 A (2); 34 (3); NVT (18)
					≥ 60 years	15	2						1		2		4			1		1			1			9N (1)| ; 20

																												(1); NVT (1)
				**2011-2014 (Early post-vaccine period)**	15y-59y	57	1	5	1	1		1	4	4		3	5			1		4			2			7A (2); 10 (1); 11 (2); 17F (2); 33 (1); 34 (1); 35F (1); NVT (15)
					≥ 60 years	26		1		1		3		2			6		1			4			1			17F (1); NVT (6)
				**2015-2019 (Mature post-vaccine period)**	15y-59y	58		1	1					1	1		5	1	5	1		4						9N (1); 11 (1); 17F (3); 23A (1); 33 (3); 34 (1); NV

																												T (28)
					≥ 60 years	12		1		1												1						9N (1); 23A (1); NVT (7)
**Ceyhan M. et al., 2016**	**Turkey**	**335**	**Pediatric**	**2008-2010 (Pre-PCV7 introduction)**	≤5y	146	11	14	1	10	4	36	8	6	4	1	6	3	9			2					6	2 (2), 7A (1), 10 (1), 15 (2), 15C (2), 16F (1), 23A (1), 15 other serotypes
					≥5y - ≤18y	56	3	2	1	2	1	3		2			4	2	1			3					7	6 (1), 7A (4), 10 (1), 15 (3), 15C (3),

																												17 (1), 12 other serotypes
				**2011-2012 (Post-PCV7/During PCV13 introduction)**	≤5y	31	1	3	2	2		3	4		1		3		3									15C (1), 23A (1), 7 other serotypes
					≥5y-≤18y	36		0	2		1	4		1	1		2	4	1			1					2	10 (1), 15 (2), 15C (2), 23A (1), 11 other serotypes
				**2013-2014 (Post-PCV13 intro**	≤5y	38	2	1	1	1		7	2	1	1	1	5		1			1					4	2 (1), 9 other serotypes

				**duction)**	≥5y - ≤18 y	28		0	1	5				1	2	2		1	1			1					3	10 (1), 15C (1), 9 other serotypes

Table S6. Antimicrobial susceptibility among IPD cases in the MENA region by country (36 original studies up to January 24, 2024)

Author, Year	Country	Total Number of tested invasive isolates	Specific classification	Tetracycline			Vancomycin			Cefotaxime			Carbapenem			Other ATB (N)		
				S	I	R	S	I	R	S	I	R	S	I	R	S	I	R
**Pre-PCV13 introduction**																		
**Hecini-Hannachi A. et al., 2013**	**Algeria**	**100**	Blood (N=22)			20	100					8	100			Levofloxacin (100), Chloramphenicol (100)		Amoxicillin (8)
			CSF (N=75)															
			Pleural fluid (N=3)															
			<17y (N=46)															
			≥18y (N=54)															
**Ramdani et al., 2015**	**Algeria**	**97**								82	13	2						
**Ziane H. et al., 2016**	**Algeria**	**80**	Meningitis (N=39)									16			17			
			Non-Meningitis (N=25)								2						Amoxicillin (3)	Amoxicillin (1)
**Al-Musawi M., 2012**	**Bahrain**	**371**	Blood, CSF, and other invasive body fluids			27												Gentamycin (174)
**Ostroff et al., 1996**	**Egypt**	**52**														Chloramphenicol (37)		Chloramphenicol (15)
**Azimian A. et al., 2020**	**Iran**	**51**	Blood (PNSP isolates)				51											
**Houri H. et al., 2017**	**Iran**	**53**	Meningitis (N=32)				53			30		2				Linezolid (53), Levofloxacin (52)		
			Non-meningitis (N=21)	6	2	13				19	1	1					Levofloxacin (1)	
**Alam A.N. et al., 2017**	**Iran**	**4**	BAL (N=2)									2			2	Ofloxacin (2)		
			Blood (N=1)									1		1		Ofloxacin (1)		

			CSF (N=1)									1		1		Ofloxacin (1)		
**Beheshti M. et al., 2020**	**Iran**	**44**	Blood			29										Linezolid (44)		Chloramphenicol (6) Levofloxacin (1)
**Saadi A. et al., 2017**	**Iraq**	**18**	CSF (N=18)				18/18 (100%)									Cephalothin 4/14(29%), Augmentin 8/16 (50%), Gentamycin 4/17 (24%)		Cephalothin 10/14(71%), Augmentin 8/16 (50%), Gentamycin 13/17 (76%)
**Mokaddas E. et al., 2008**	**Kuwait**	**122**	Bronchiol aspirates (N=3)			1											Chloramphenicol (1)	Cefuroxime (1)
			Pleural Fluid (N=2)		1	1											Cefuroxime (1)	Cefuroxime (1)
			Tracheal secretions (N=46)		2	25					1			3	3		Ampicillin (12), Cefuroxime (5), Chloramphenicol (4)	Ampicillin (1), Cefuroxime (19)
			Blood (N=68)			12								6			Ampicillin (15), Cefuroxime (7), Chloramphenicol (4)	Ampicillin (1), Cefuroxime (6)
			CSF (N=3)															
**Moghnieh, et al.,2019**	**Lebanon**	**37**																Levofloxacin (1)
**ElMdaghri N. et al., 2012**	**Morocco**	**24**																Amoxicillin (1)
**ElMdaghri N. et al., 2012**	**Morocco**	**187**	1994-2001 (Pre-PCV7 introduction)		25												Chloramphenicol (4)	
			2006-2010 (Pre-PCV13 introduction)		34												Chloramphenicol (11), Amoxicillin (4)	

**Mastro T.D. et al., 1991**	**Pakistan**	**87**				70												Chloramphenicol (34)
**Kattan R. et al., 2011**	**Palestinian Territories**	**120**	Blood				120			120						Ofloxacin (118)		Ofloxacin (2)
**El-Shafie S. et al., 2016**	**Qatar**	**118**		68	5	45	118			97	19	2				Amoxicillin (93), Chloramphenicol (104)	Amoxicillin (3)	Amoxicillin (1), Chloramphenicol (14)
**Chowdhury M. et al., 1995**	**Saudi Arabia**	**27**	Blood (N=22)		5													
			CSF (N=3)		1													
			Joint Fluid (N= 2)		0													
**Kambal A. et al., 1997**	**Saudi Arabia**	**49**				13												Combined Resistance: Penicillin+Erythromycin (2), Penicillin+Tetracycline (2), Penicillin+Clindamycin (2), Erythrocyclin+Tetracycline (3), Penicillin+Erythrocyclin+Tetracycline (3)
**Al-Aqeeli A. et al., 2002**	**Saudi Arabia**	**172**					172											
**Al Tawfiq et al., 2004**	**Saudi Arabia**	**62**	Blood (N=58)	44		14												
			CSF (N=4)															
**Fouda S. I., et al., 2004**	**Saudi Arabia**	**107**	Blood and CSF													Roxithromycin (7) Azithromycin (5) Clarithromycin (4)	Roxithromycin (3) Azithromycin (3) Clarithromycin (4)	Roxithromycin (11) Azithromycin (11) Clarithromycin (12), Amox-Clav (3), Ampicillin (12), Cefprozil (8), Cefaclor (16), Cefdinir (14), Cefixime (15), Cefuroxime (5),
**Al-Tawfiq J. A., 2006**	**Saudi Arabia**	**50**	Blood															
**Shibl A. et al., 2008**	**Saudi Arabia**	**350**	Blood (N=287)									23						
			CSF and other (N=63)															

**Smaoui H. et al., 2009**	**Tunisia**	**106**	Blood (N=40)									LR:4, HR:0					Amoxicillin: LR: 6, HR:0	
			CSF (N=52)									LR:1, HR:0					Amoxicillin: LR: 4, HR:1	
			Other invasive samples (joints, pleural and intraabdominal) (N=14)									LR:1, HR:0					Amoxicillin: LR: 1, HR:0	
**Raddaoui, 2015**	**Tunisia**	**9**	Blood													Levofloxacin (9)		Chloramphenicol (2)
**Ktari S. et al., 2023**	**Tunisia**	**106**	Meningococcal isolates (n=31)			9						5				Levofloxacin (31)		Amoxicillin (13); Cotrimoxazole (3); Chloramphenicol (1)
			Non-meningococcal isolates (n=75)									12						Amoxicillin (34)
**Post-PCV13 introduction**																		
**Ikken Y. et al., 2020**	**Morocco**	**65**	CSF (N=65)	48	3	9			65	62	2	1				Oxacillin (50) Chloramphenicol (59) Levofloxacin (62) Rifampin (63) Amoxicillin (60)		Oxacillin (15) Chloramphenicol (6) Levofloxacin (2) Rifampin (2) Amoxicillin (5)
**Al-Jardani et al., 2019**	**Oman**	**132**	Meningitis (N=23)				132			22	1	0	130		2	Amoxicillin (129), Levofloxacin (131), Chloramphenicol (130)	Amoxicillin (2)	Amoxicillin (0), Levofloxacin (1), Chloramphenicol (2)
			Non-meningitis (N=109)							108	1	0						
**Al-Sherikh et al, 2014**	**Saudi Arabia**	**78**	Meningitis (N=32)							0	0	32						
			Non-meningitis (N=46)							0	46	0						
			All cases				78						78			Linezolid (78), Levofloxacin (78)		

**Kittana F.N.A. et al., 2019**	**Turkey**	**110**		18	3	89										Chloramphenicol (91) Kanamycin (2)	Kanamycin (2)	Chloramphenicol (19) Kanamycin (106)
**Pre- and post-PCV13 introduction**																		
**Reslan L. et al., 2022**	**Lebanon**	**537**	**2005-2009 (PCV7 era)**															
			≤5y	39	2	21	63									Chloramphenicol (52); Levofloxacin (48)		Chloramphenicol (4); Levofloxacin (2)
			6y-60y	32	6	12	50									Chloramphenicol (36); Levofloxacin (37)		Chloramphenicol (6); Levofloxacin (3)
			>60y	36	9	10	56									Chloramphenicol (40); Levofloxacin (37)		Chloramphenicol (9); Levofloxacin (2)
			**2010-2015 (Post-PCV7/pre-PCV13 era)**															
			≤5y	44	4	25	77		1							Chloramphenicol (53); Levofloxacin (72)		Chloramphenicol (6); Levofloxacin (1)
			6y-60y	43	2	14	62		1							Chloramphenicol (41); Levofloxacin (56)		Chloramphenicol (7); Levofloxacin (3)
			>60y	37	3	18	63		0							Chloramphenicol (42); Levofloxacin (55)	Levofloxacin (1)	Chloramphenicol (2)
			**2016-2020 (PCV13 era)**															
			≤5y	46	2	9	56		1							Chloramphenicol	Levofloxacin (1)	Chloramphenicol (4); Levofloxacin (1)

																(52); Levofloxacin (50)		
			6y-60y	38	1	9	46									Chloramphenicol (43); Levofloxacin (45)	Levofloxacin (1)	Chloramphenicol (3); Levofloxacin (2)
			>60y	42		8	51									Chloramphenicol (50); Levofloxacin (48)		Levofloxacin (1)
**Diawara I. et al., 2015**	**Morocco**	**136**	**Period 1 (pre-PCV13 implementation): ≤2y (N=79)**		25												Chloramphenicol (8)	
			**Period 1 (pre-PCV13 implementation): 2y - <5y (N=12)**		6												Chloramphenicol (1)	
			**period 2 (post-PCV13 implentation): ≤2y (N=32)**		7												Chloramphenicol (1)	
			**Period 2 (post-PCV13 implementation):2y - <5y (N=13)**		3												Chloramphenicol (1)	
**Nzoyikorera N. et al., 2023**	**Morocco**	**239**	**2007-2010 (Pre-vaccine period)**															
			15y-59y		22												Cotrimoxazole (21); Chloramphenicol (3)	
			≥ 60 years		4												Cotrimoxazole (2); Chloramphenicol (3)	
			**2011-2014 (Early post-vaccine period)**															
			15y-59y		11												Cotrimoxazole (5); Chloramphenicol (3)	
			≥ 60 years		6												Cotrimoxazole (2); Chloramphenicol (1)	

			**2015-2019 (Mature post-vaccine period)**															
			15y-59y		23												Cotrimoxazole (5); Chloramphenicol (3)	
			≥ 60 years		3												Chloramphenicol (1)	
**Al-Waili B.R. et al., 2013**	**Saudi Arabia**	**208**	**Period 1 (2006-2008) (N=76)** (Before the revision of breakpoints for meningitis, non-meningitis intravenous and oral administration)						0						4/76			Parenteral Cefuroxime (8/76) Cefotaxime/ceftriaxone non-Meningeal (0/76) Cefotaxime/ceftriaxone Meningeal (16/76)
			**Period 2 (2008-2012) (N=132)** (After the revision of breakpoints for meningitis, non-meningitis intravenous and oral administration)						0						5/132			Oral Cefuroxime (48/132) Parenteral Cefuroxime (64/132) Cefotaxime/ceftriaxone non-Meningeal (4/132) Cefotaxime/ceftriaxone Meningeal (23/132)
**Unspecified period**																		
**Haifa Al-Muhtaresh A. & Bindayna K.M., 2020**	**Kingdom of Bahrain**	**22**	**Blood (N=21)**			9												
			**CSF (N=1)**			1												

Table S7. Antimicrobial susceptibility among IPD cases in the MENA region by country and detected serotypes (13 original studies up to January 24, 2024)

Author, Year	Country	Total Number of tested IPD cases	ATB	Antimicrobial Sensitivity	4	6B	9V	14	18C	19F	23F	1	5	7F	3	6A	19A	22F	33F	8	10A	11A	12F	15B	Non-typeable	Other serotypes (N)
**Pre-PCV13 introduction**																										
**Hecini-Hannachi A. et al., 2013**	**Algeria**	**100**	**Penicillin**	**R (N=43)**		4		15	3	8	5	1		2	3		2				2					
			**Erythromycin**	**R (N=16)**				5		7				1			2				1					
**Wasfy M. et al., 2005**	**Egypt**	**205**	**Penicillin**	**I (N=97)**		21		7	2	4	9		1			11	14			1	1			1	10	9A (1), 10B (1), 15A (4), 16F (1), 20 (2), 22A (2), 23B (1), 35 (2), Pool C (2), Pool E (1), Pool G (1), Pool I (2)
				**R (N=3)**						1	2															
			**Erythromycin**	**I and R (N=26)**		6		2		2	2	1				4	1								1	2 (1), 18A (1), 20 (1), Pool C (2), Pool H (1), Pool I (1)
			**TMP/SMX**	**I and R (N=149)**	1	20		6	1	8	13	10	5	1	1	10	14			2	1			1	8	2 (2), 7A (1), 7B (1), 7C (1), 9A (2), 10B (1), 10F (1), 11C (1), 15A (3), 16F (5), 17F (1), 18A (1), 20 (2), 22A (1), 23B (4), 33C (1), 35 (2), 37 (1), Pool C (2), Pool D (2), Pool E (2), Pool F (1), Pool G (1), Pool H (1), Pool I (7)

			**Ceftriaxone**	**I (N=10)**				1		1	6									1						
				**R (N=3)**						1	2															
			**Chloramphenicol**	**I and R (N=21)**		1					7	1	1			1									2	2 (2), 7A (1), 15A (1), 31 (2), Pool I (2)
			**Tetracycline**	**I and R (N=109)**	1	6		8	2	5	12	15	2	1	1	5	12			1	2	1		1	5	2 (3), 7A (1), 10B (1), 12A (1), 15A (1), 15C (1), 15F (1), 17F (1), 18A (1), 20 (3), 23B (4), 31 (1), Pool C (3), Pool D (1), Pool E (2), Pool H (1), Pool I (3)
**Mosadegh M. et al., 2022**	**Iran**	**53 (All PNSP)**	**Ceftriaxone**	**I (N=19)**			1	7		5	3															6A/6B (1); NVT (2)
				**R (N=14)**			2	2		3	2				3		1									NVT (1)
			**Cefotaxime**	**I (N=13)**			2	3		3	2				1											NVT (2)
				**R (N=16)**			2	3		4	2				3		1									NVT (1)
**Mokaddas et al., 2008**	**Kuwait**	**43**	**Penicillin**	**S (n=16)**	2	0	0	0		0	1	3	2		1	1	2				1					11C (1), 16F (1), 17F (1)
				**I (N=22)**	0	2	2	5		2	3	0	0		0	1	2				1					9A (1), 15A (3)
				**R (N=5)**	0	0	2	2		0	1	0	0		0	0	0				0					
**Mokaddas E.M et al., 2012**	**Kuwait**	**129**	**Penicillin**	**R (N=2)**							1						1									
**ElMdaghri N. et al., 2012**	**Morocco**	**187**	**Penicillin**	**I and R (N=66)**		7		24		4	6		2	1			7			1					3	2 (1), 7 (2), 9 (1), 19 (3), 23 (4)

**Al-Yaqoubi and Elhag, 2011**	**Oman**	**34**	**Penicillin**	**R (N=11)**		2		2		1	2					1						1			1	9A (1)
**Mastro et al., 1991**	**Pakistan**	**87**	**Chloramphenicol**	**R (N=14)**			9						1			4										
			**Tetracyclin**	**R (N=29)**		1				17		1														Serogroup 16 (10)
			**MDR**							7 (tetracycline and penicillin)/4 (Chloramphenicol and tetracyclin)			1 (co-trimoxazole and tetracyclin)				1 (co-trimoxazole and penicillin and tertracycline)/9 (chloramphenicol and tetracycline)									serotype 31: 1 MDR to Chloramphenicol and tetracyclin/12 MDR to co-trimoxazole, chloromphenicol and tetracycline;<br>15C (4 MDR to chloramphenicol and tetracycline);<br>Serogroup 16 (2 MDR-chloramphenicol and tetracyclin)
**Kattan R. et al., 2011**	**Palestinian Territories**	**120**	**Penicillin**	**S (N=120)**	6		11	16		8	3	14	9	4			5		2	4			2		3	6A/B (17), 16F (2), Sg18 (5), 17F (1), 35B (3), 38F (1)
			**Erythromycin**	**S (N=82)**	4		10	4		5	3	13	8	2	1		3		1	3			2		3	6A/B (8), 16F (2), Sg18 (5), 17F (1), 35B (3), 38F (1)
				**I (N=5)**	1							1			1		1									6A/B (1)
				**R (N=33)**	1		1	12		3			1	2	2		1		1	1						6A/B (8)
			**TMP/SMX**	**S (N=46)**	5		3	3		3		1	1		2		2			1			1		3	6A/B (9), 16F (2), Sg18 (5), 17F (1), 35B (3), 38F (1)

				**I (N=21)**			2	1		1		5	6				1			2			1			6A/B (2)
				**R (N=53)**	1		6	12		4	3	8	2	4	2		2		2	1						6A/B (6)
			**Vancomycin**	**S (N=120)**	6		11	16		8	3	14	9	4			5		2	4			2		3	6A/B (17), 16F (2), Sg18 (5), 17F (1),35B (3), 38F (1)
			**Cefotaxime**	**S (N=120)**	6		11	16		8	3	14	9	4			5		2	4			2		3	6A/B (17), 16F (2), Sg18 (5), 17F (1),35B (3), 38F (1)
			**Ofloxacin**	**S (N=118)**	6		11	14		8	3	14	9	4			5		2	4			2		3	6A/B (17), 16F (2), Sg18 (5), 17F (1),35B (3), 38F (1)
				**I (N=2)**				2																		
**Shibl, 2008**	**Saudi Arabia**	**350**	**Penicillin**	**S (N=161)**	6	15	6	20	7	4	19	3	4		5	1	3			6					25	Serogroup 7 (6), serogroup 11 (6), serogroup 12 (7), serogroup 15 (3), serogroup 22 (4), 23A (4), 23B (2), 24 (5)
				**I (N=147)**	4	14	9	31	12	19	21	0	0		0	3	1			0					1	serogroup 15 (2), 23A (1), 23B (2), 24 (2)
				**R (N=42)**	1	7	3	4	1	8	6	1	1		2	2	2			0					1	serogroup 15 (1), 23A (1), 23B (1)
			**Erythromycin**	**R (N=91)**	3	16	3	13	5	11	19	2	1		2	3	2			0					5	serogroup 15 (3), 23B (1), 24 (2)
			**Cefotaxime**	**R (N=23)**	2	4	0	4	0	3	3	1	1		0	2	0			0					0	23A (1)
**Ktari S. et al., 2023**	**Tunisia**	**106**	**Penicillin**	**S (N=35)**	2	2	2		5			1		1	12	1										7C (1); 9N (1), 13 (1); 16F (2); 17F (2); 24F (1); 34 (1)
				**I (N=59)**		6	3	13	1	11	4					7	4									6C (1); 9A (2); 9N (2); 17F (1); 35B (3); 35F (1)
				**R (N=12)**			1	5		4							1									9A (1)

			**Cefotaxime**	**S (N=89)**	2	8	6	12	6	8	2	1		1	12	8	4									6C (1); 7C (1); 9A (3); 9N (3); 13 (1); 16F (2); 17F (3); 24F (1); 34 (1); 35B (2); 35F (1)
				**I (N=17)**				6		7	2						1									35B (1)
			**Tetracycline**	**S (N=66)**	2	2	4	14	5	4	3	1		1	10	6	1									7C (1); 9N (1); 13 (1); 16F (2); 17F (3); 34 (1); 35B (3); 35F (1)
				**I (N=5)**				1		2					1		1									
				**R (N=35)**		6	2	3	1	9	1				1	2	3									6C (1); 9A (3); 9N (2); 24F (1)
			**Erythromycin**	**S (N=39)**	2	1	4	4	4	1		1		1	9	2										7C (1); 9N (1); 13 (1); 16F (2); 17F (2); 34 (1); 35B (1); 35F (1)
				**I (N=1)**													1									
				**R (N=66)**		7	2	14	2	14	4				3	6	4									6C (1); 9A (3); 9N (2); 17F (1); 24F (1); 35B (2)
**Pre- and post-PCV13 introduction**																										
**Reslan L. et al., 2022**	**Lebanon**	**542**	**Penicillin**	**S (N=387)**	12	11	18	20	12	21	11	33	16	9	38	12	16	13	8	5	5	6	7	8	19	2 (4); 6C (2); 7C/7B/40F (2); 9N (7); 10B (2); 10F/10C/33C (2); 13 (3); 15A/15F (4); 16F (7); 17F (2); 20 (2); 21 (2); 23A (5); 23B (5); 23F (11); 24F (9); 28A (1); 29 (2); 31 (5); 34 (4); 35A/35C/42 (1); 35F/47 (2); 38/25 (2); 42 (1);
				**I (N=20)**			2	4		11												1				2 (1); 9N (1)
				**R (N=43)**		5	1	6		9	3					3	1					2	2			16F (1); 23F (3); 24F (1); 35B (2); 35F/47 (1); 38/25 (1); 9N (2);
			**Ceftriaxone**	**S (N=384)**	11	16	14	24	12	22	12	33	15	8	37	13	21	13	6	5	5	8	8	9	18	2 (3); 6C (1); 7C/7B/40F (1); 9N (8); 10B (2); 13 (3); 15A/15F (4); 16F (7); 17F (1); 20 (2); 21 (2); 23A (5); 23B (5); 24F (9); 28A (1); 29 (2); 31 (5);

																										34 (4); 35A/35C/42 (1); 35B (2); 35F/47 (3); 38/25 (3)
				**I (N=23)**			3	6		7	1				1		1					1			2	9N (1)
				**R (N=13)**			1			11													1			
			**Tetracycline**	**S (N=349)**	14	14	14	15	13	17	7	34	13	8	38	15	12	14	4	4	4	8	5	10	19	2 (5); 6C (2); 7C/7B/40F (2); 9N (7); 10B (1); 10F/10C/33C (2); 13 (3); 15A/15F (3); 16F (8); 17F (1); 20 (1); 21 (2); 23A (3); 23B (5); 24F (5); 28A (1); 29 (2); 31 (5); 34 (2); 35B (2); 35F/47 (3); 38/25 (1); 42 (1)
				**I (N=22)**		2	1		1	2	1	3	1	1	1	1	2		2	1	1					9N (2)
				**R (N=125)**		2	6	20	2	26	10	2	1	2	3	3	14	1				1	7	1	6	2 (1); 10F/10C/33C (1); 15A/15F (1); 20 (1); 23A (2); 24F (6); 34 (2); 35A/35C/42 (1); 38/25 (1); 9N (2)
			**Erythromycin**	**S (N=358)**	14	16	15	13	14	13	8	34	16	9	44	13	9	16	5	5	5	9	4	11	16	2 (4); 6C (1); 7C/7B/40F (2); 9N (10); 10B (2); 10F/10C/33C (1); 13 (4); 15A/15F (3); 16F (7); 17F (1); 20 (2); 21 (2); 23A (3); 23B (5); 24F (3); 28A (1); 29 (2); 31 (5); 34 (3); 35B (2); 35F/47 (3); 38/25 (2); 42 (1)
				**I (N=8)**							1				1	1	1								1	2 (1); 24F (1); 35A/35C/42 (1)
				**R (N=153)**		2	6	25	2	34	11	6		2	2	5	19		3				8	1	10	10F/10C/33C (2); 15A/15F (1); 16F (2); 2 (1); 23A (1); 24F (7); 38/25 (1); 6C (1); 9N (1)
			**Chloramphenicol**	**S (N=409)**	10	17	15	29	14	35	15	27	15	11	38	16	20	9	6	3	5	9	10	10	22	2 (5); 6C (1); 7C/7B/40F (2); 9N (9); 10B (2); 10F/10C/33C (2); 13 (4); 15A/15F (3); 16F (7); 17F (1); 20 (1); 21 (2); 23A (4); 23B (4); 24F (10); 29 (2); 31 (5); 34 (1); 35A/35C/42 (1); 35B (2); 35F/47 (3); 38/25 (2)
				**R (N=40)**	1		3	3		4	2	3			1	1	5	3	1				1		1	2 (1); 6C (1); 16F (2); 23A (1); 23B (1); 34 (3); 9N (2)

			**Clindamycin**	**S (N=212)**	5	6	8	8	6	11	8	24	6	3	23	7	8	6	3	2	1	4	2	5	16	2 (4); 6C (1); 7C/7B/40F (2); 9N (6); 10B (2); 10F/10C/33C (1); 13 (3); 15A/15F (1); 16F (6); 17F (1); 20 (1); 21 (2); 23A (3); 23B (4); 24F (3); 31 (4); 34 (1); 35B (2); 35F/47 (2); 38/25 (1)
				**I (N=5)**				1		1							1									23B (1); 34 (1)
				**R (N=64)**			1	10	2	6	2	2	3	1	3	3	6						6	1	4	2 (2); 10F/10C/33C (1); 15A/15F (1); 23A (1); 24F (7); 34 (1); 35A/35C/42 (1)
			**TMP/SMX**	**S (N=201)**	4	5	2	8	11	8	3	13	1	5	36	8	14	11	3	2	1	1	3	5	14	2 (4); 7C/7B/40F (2); 9N (2); 10B (1); 10F/10C/33C (2); 13 (2); 15A/15F (3); 16F (2); 17F (1); 23A (2); 23B (2); 24F (3); 28A (1); 29 (2); 31 (4); 34 (1); 35A/35C/42 (1); 35B (2); 35F/47 (3); 38/25 (3)
				**I (N=43)**		2		3	2	2	2	5		3	4	4	2	4	2	1	1			2		24F (2); 34 (1); 9N (1)
				**R (N=242)**	8	10	18	26	2	33	12	20	13	3	3	7	12	1		1	3	5	8	5	10	2 (2); 6C (2); 9N (8); 10B (1); 10F/10C/33C (1); 13 (2); 15A/15F (1); 16F (5); 20 (2); 21 (2); 23A (3); 23B (3); 24F (6); 31 (1); 34 (2); 42 (1)
			**Levofloxacin**	**S (N=448)**	11	15	20	33	13	41	16	37	13	10	39	17	27	12	6	4	5	9	9	10	24	2 (5); 6C (2); 7C/7B/40F (2); 9N (8); 10B (2); 10F/10C/33C (3); 13 (3); 15A/15F (4); 16F (7); 17F (1); 20 (1); 21 (2); 23A (4); 23B (5); 24F (10); 28A (1); 29 (1); 31 (5); 34 (3); 35A/35C/42 (1); 35B (2); 35F/47 (3); 38/25 (2)
				**I (N=3)**			1																			16F (2)
				**R (N=14)**				1	1	1		1	2		1	1				1			1	1	1	23A (1); 24F (1)
			**Vancomycin**	**S (N=504)**	13	18	20	37	16	46	19	39	16	11	46	18	28	16	8	5	5	7	10	11	24	2 (6); 6C (2); 7C/7B/40F (2); 9N (11); 10B (2); 10F/10C/33C (3); 13 (4); 15A/15F (4); 16F

																										(9); 17F (1); 20 (2); 21 (2); 23A (5); 23B (5); 24F (11); 28A (1); 29 (2); 31 (5); 34 (4); 35A/35/42 (1); 35B (2); 35F/47 (3); 38/25 (3); 42 (1)
				**R (N=3)**						1		1				1										
**Unspecified period**																										
**Haifa Al-Muhtaresh A. & Bindayna K.M., 2020**	**Kingdom of Bahrain**	**17**	**Penicillin**	**S (N=5)**				0							2											6 (2), 19 (1)
				**I (N=7)**				1							0											6 (5), 19 (1)
				**R (N=4)**				0							0											6 (1), 19 (2), 23 (1)
			**Erythromycin**	**S (N=8)**				0							2											6 (4), 19 (2)
				**R (N=8)**				1							0											6 (4), 19 (2), 23 (1)
			**Tetracycline**	**S (N=10)**				0							2											6 (6), 19 (2)
				**R (N=7)**				1							0											6 (3), 19 (2), 23 (1)
